# Supplementary material for: Instructing children to construct ideas into products alters children’s creative idea selection in a randomized field experiment
Source: PLoS One. 2022 Aug 4;17(8):e0271621. doi: 10.1371/journal.pone.0271621 (PMC9352014; doi:10.1371/journal.pone.0271621)
Supplement: S1 File — (DOCX) [file pone.0271621.s001.docx]

**Supporting Information for:**

Instructing children to construct ideas into products alters children’s creative idea selection in a randomized field experiment

**Authors:** Kim van Broekhoven, Barbara Belfi, and Lex Borghans

Corresponding author: Kim van Broekhoven

Email: [kim.vanbroekhoven@ru.nl](mailto:kim.vanbroekhoven@ru.nl)

**This PDF file includes:**

Supplementary Tables

S1 Table p. 2

S2 Table p. 3

S3 Table p. 5

Supplementary Figures

S1 Figure p. 8

Appendices

S1 Appendix p. 9

S2 Appendix p. 35

S3 Appendix p. 54

S4 Appendix p. 55

Supplementary Files

S1 File. Dataset for this study (<https://osf.io/thnyu/>)

S2 File. Syntax for this study (<https://osf.io/thnyu/>)

**Tables**

**S1 Table.** Tests of between-condition differences on all available demographic variables and measured psychological variables

|  | **Treatment** | | | |  |  | **Control** | | | | **Test of between-condition difference** |
| --- | --- | --- | --- | --- | --- | --- | --- | --- | --- | --- | --- |
|  | *n* |  | % or *M* (*SD*) | |  |  | *n* |  | % or *M* (*SD*) | |  |
| **Demographic variables** |  |  |  |  |  |  |  |  |  |  |  |
| Gender | 109 |  | 54% | Girls |  |  | 92 |  | 46% | Girls | χ2(1) = 3.04, *p* = 0.081 |
|  | 92 |  | 46% | Boys |  |  | 110 |  | 54% | Boys |  |
| Age (in years) | 175 |  | 11.58 | (0.50) |  |  | 180 |  | 11.61 | (0.49) | *t*(1,401) = -1.75, *p* = 0.082 |
| Ethnicity | 190 |  | 97% | Dutch |  |  | 190 |  | 97% | Dutch | χ2(1) = 0.09, *p* = 0.766 |
|  | 5 |  | 3% | Other |  |  | 6 |  | 3% | Other |  |
| Socioeconomic status | 26 |  | 15% | Low |  |  | 18 |  | 10% | Low | χ2(2) = 2.11, *p* = 0.349 |
|  | 135 |  | 76% | Middle |  |  | 135 |  | 78% | Middle |  |
|  | 16 |  | 9% | High |  |  | 21 |  | 12% | High |  |
| Report grade (1-10) | 182 |  | 8.02 | (0.98) |  |  | 185 |  | 7.87 | (0.98) | *t*(1,365) = -1.54, *p* = 0.124 |
| School | 4 |  | 2% | Site #1 |  |  | 9 |  | 5% | Site #1 | χ2(12) = 10.65, *p* = 0.559 |
|  | 10 |  | 5% | Site #2 |  |  | 8 |  | 4% | Site #2 |  |
|  | 5 |  | 3% | Site #3 |  |  | 9 |  | 5% | Site #3 |  |
|  | 17 |  | 9% | Site #4 |  |  | 18 |  | 9% | Site #4 |  |
|  | 5 |  | 3% | Site #5 |  |  | 10 |  | 5% | Site #5 |  |
|  | 25 |  | 12% | Site #6 |  |  | 25 |  | 12% | Site #6 |  |
|  | 19 |  | 10% | Site #7 |  |  | 15 |  | 7% | Site #7 |  |
|  | 23 |  | 11% | Site #8 |  |  | 31 |  | 15% | Site #8 |  |
|  | 17 |  | 9% | Site #9 |  |  | 15 |  | 7% | Site #9 |  |
|  | 33 |  | 16% | Site #10 |  |  | 21 |  | 10% | Site #10 |  |
|  | 17 |  | 9% | Site #11 |  |  | 12 |  | 6% | Site #11 |  |
|  | 5 |  | 3% | Site #12 |  |  | 7 |  | 4% | Site #12 |  |
|  | 21 |  | 11% | Site #13 |  |  | 22 |  | 11% | Site #13 |  |
| **Psychological variables** |  |  |  |  |  |  |  |  |  |  |  |
| Risk preference | 178 |  | 2.90 | (0.78) |  |  | 173 |  | 2.92 | (0.69) | *t*(1,349) = 0.22, *p* = 0.827 |
| Personality traits |  |  |  |  |  |  |  |  |  |  |  |
| *Openness to experience* | 180 |  | 3.51 | (0.61) |  |  | 173 |  | 3.40 | (0.66) | *t*(1,351) = -1.66, *p* = 0.098 |
| *Conscientiousness* | 180 |  | 3.51 | (0.68) |  |  | 173 |  | 3.52 | (0.67) | *t*(1,351) = 0.17, *p* = 0.869 |
| *Extraversion* | 180 |  | 3.25 | (0.62) |  |  | 173 |  | 3.21 | (0.62) | *t*(1,351) = -0.73, *p* = 0.464 |
| *Agreeableness* | 180 |  | 3.94 | (0.58) |  |  | 173 |  | 3.92 | (0.58) | *t*(1,351) = -0.33, *p* = 0.743 |
| *Emotional stability* | 180 |  | 2.90 | (0.74) |  |  | 173 |  | 2.94 | (0.71) | *t*(1,351) = 0.55, *p* = 0.585 |
|  |  |  |  |  |  |  |  |  |  |  |  |
| Manipulation check | 115 |  | 1.07 | (0.26) |  |  | 98 |  | 1.90 | (0.30) | *t*(1,211) = 21.60, *p* = 0.000 |

**S2 Table.** Regression estimated effect of expected implementation on children’s feasibility and originality ratings

|  | **Feasibility ratings** | | | | | |  |  | **Originality ratings** | | | | | |
| --- | --- | --- | --- | --- | --- | --- | --- | --- | --- | --- | --- | --- | --- | --- |
|  | M1 | |  |  | M2 | |  |  | M1 | |  |  | M2 | |
|  | Main effect | |  |  | Incl. interaction-analyses | |  |  | Main effect | |  |  | Incl.  interaction-analyses | |
|  | B |  |  |  | B |  |  |  | B |  |  |  | B |  |
|  | *(SE)* | *d* |  |  | *(SE)* | *d* |  |  | *(SE)* | *d* |  |  | *(SE)* | *d* |
| Intercept | 2.121*** |  |  |  | 2.645 |  |  |  | 3.795*** |  |  |  | 5.277* |  |
|  | (0.080) |  |  |  | (2.090) |  |  |  | (0.081) |  |  |  | (2.254) |  |
| Expected implementation | 1.162*** | 1.028 |  |  | 3.479* | 3.225 |  |  | -0.434*** | 0.377 |  |  | -0.068 | 1.268 |
|  | (0.113) |  |  |  | (1.486) |  |  |  | (0.115) |  |  |  | (-1.602) |  |
| **Demographic variables** |  |  |  |  |  |  |  |  |  |  |  |  |  |  |
| Gender (ref.= boys) |  |  |  |  | 0.097 |  |  |  |  |  |  |  | 0.140 |  |
|  |  |  |  |  | (0.158) |  |  |  |  |  |  |  | (0.170) |  |
| Age (in years) |  |  |  |  | -0.156 |  |  |  |  |  |  |  | -0.071 |  |
|  |  |  |  |  | (0.157) |  |  |  |  |  |  |  | (0.170) |  |
| Ethnicity (ref. = Dutch) |  |  |  |  | 0.414 |  |  |  |  |  |  |  | -0.038 |  |
|  |  |  |  |  | (0.478) |  |  |  |  |  |  |  | (0.515) |  |
| Socio-economic status  (ref. = middle) |  |  |  |  |  |  |  |  |  |  |  |  |  |  |
| Low |  |  |  |  | -0.331 |  |  |  |  |  |  |  | -0.438 |  |
|  |  |  |  |  | (0.534) |  |  |  |  |  |  |  | (0.575) |  |
| High |  |  |  |  | 0.195 |  |  |  |  |  |  |  | 0.338 |  |
|  |  |  |  |  | (0.332) |  |  |  |  |  |  |  | (0.359) |  |
| Report grade |  |  |  |  | 0.095 |  |  |  |  |  |  |  | 0.000 |  |
|  |  |  |  |  | (0.080) |  |  |  |  |  |  |  | (0.086) |  |
| School  (ref. = school site # 10) |  |  |  |  |  |  |  |  |  |  |  |  |  |  |
| Site #1 |  |  |  |  | 0.496 |  |  |  |  |  |  |  | 0.366 |  |
|  |  |  |  |  | (1.105) |  |  |  |  |  |  |  | -1.192 |  |
| Site #2 |  |  |  |  | 0.485 |  |  |  |  |  |  |  | -0.781 |  |
|  |  |  |  |  | (0.814) |  |  |  |  |  |  |  | (0.877) |  |
| Site #3 |  |  |  |  | -0.207 |  |  |  |  |  |  |  | -.005 |  |
|  |  |  |  |  | (0.347) |  |  |  |  |  |  |  | (0.374) |  |
| Site #4 |  |  |  |  | -0.227 |  |  |  |  |  |  |  | -0.299 |  |
|  |  |  |  |  | (0.267) |  |  |  |  |  |  |  | (0.287) |  |
| Site #5 |  |  |  |  | 0.035 |  |  |  |  |  |  |  | -0.124 |  |
|  |  |  |  |  | (0.355) |  |  |  |  |  |  |  | (0.383) |  |
| Site #6 |  |  |  |  | -0.105 |  |  |  |  |  |  |  | -0.264 |  |
|  |  |  |  |  | (0.241) |  |  |  |  |  |  |  | (0.260) |  |
| Site #7 |  |  |  |  | 0.111 |  |  |  |  |  |  |  | -0.409 |  |
|  |  |  |  |  | (0.727) |  |  |  |  |  |  |  | (0.784) |  |
| Site #8 |  |  |  |  | -0.262 |  |  |  |  |  |  |  | -0.406 |  |
|  |  |  |  |  | (0.339) |  |  |  |  |  |  |  | (0.366) |  |
| Site #9 |  |  |  |  | 0.108 |  |  |  |  |  |  |  | -1.158** |  |
|  |  |  |  |  | (0.396) |  |  |  |  |  |  |  | (0.427) |  |
| Site #11 |  |  |  |  | 0.554* |  |  |  |  |  |  |  | -0.687 |  |
|  |  |  |  |  | (0.263) |  |  |  |  |  |  |  | (0.284) |  |
| Site #12 |  |  |  |  | -0.406 |  |  |  |  |  |  |  | -0.462 |  |
|  |  |  |  |  | (0.390) |  |  |  |  |  |  |  | (0.420) |  |
| Site #13 |  |  |  |  | 0.337 |  |  |  |  |  |  |  | -0.579 |  |
|  |  |  |  |  | (0.384) |  |  |  |  |  |  |  | (0.414) |  |
| **Psychological variables** |  |  |  |  |  |  |  |  |  |  |  |  |  |  |
| Risk preference |  |  |  |  | 0.076 |  |  |  |  |  |  |  | 0.084 |  |
|  |  |  |  |  | (0.162) |  |  |  |  |  |  |  | (0.175) |  |
| Personality traits |  |  |  |  |  |  |  |  |  |  |  |  |  |  |
| Openness to experience |  |  |  |  | 0.034 |  |  |  |  |  |  |  | -0.013 |  |
|  |  |  |  |  | (0.165) |  |  |  |  |  |  |  | (0.178) |  |
| Conscientiousness |  |  |  |  | 0.397* |  |  |  |  |  |  |  | -0.337 |  |
|  |  |  |  |  | (0.169) |  |  |  |  |  |  |  | (0.183) |  |
| Extraversion |  |  |  |  | -0.152 |  |  |  |  |  |  |  | 0.057 |  |
|  |  |  |  |  | (0.178) |  |  |  |  |  |  |  | (0.192) |  |
| Agreeableness |  |  |  |  | -0.129 |  |  |  |  |  |  |  | 0.071 |  |
|  |  |  |  |  | (0.200) |  |  |  |  |  |  |  | (0.216) |  |
| Emotional stability |  |  |  |  | -0.120 |  |  |  |  |  |  |  | 0.020 |  |
|  |  |  |  |  | (0.145) |  |  |  |  |  |  |  | (0.156) |  |
| **Interaction with  psychological variables** |  |  |  |  |  |  |  |  |  |  |  |  |  |  |
| Implementation*  risk preference |  |  |  |  | -0.267 |  |  |  |  |  |  |  | 0.052 |  |
|  |  |  |  |  | (0.226) |  |  |  |  |  |  |  | (0.244) |  |
| Implementation*  openness |  |  |  |  | -0.109 |  |  |  |  |  |  |  | 0.164 |  |
|  |  |  |  |  | (0.255) |  |  |  |  |  |  |  | (0.274) |  |
| Implementation*  conscientiousness |  |  |  |  | -0.608* |  |  |  |  |  |  |  | 0.455 |  |
|  |  |  |  |  | (0.234) |  |  |  |  |  |  |  | (0.252) |  |
| Implementation*  extraversion |  |  |  |  | 0.289 |  |  |  |  |  |  |  | -0.286 |  |
|  |  |  |  |  | (0.250) |  |  |  |  |  |  |  | (0.269) |  |
| Implementation*  agreeableness |  |  |  |  | -0.022 |  |  |  |  |  |  |  | -0.483 |  |
|  |  |  |  |  | (0.289) |  |  |  |  |  |  |  | (0.312) |  |
| Implementation*  emotional stability |  |  |  |  | 0.053 |  |  |  |  |  |  |  | 0.035 |  |
|  |  |  |  |  | (0.204) |  |  |  |  |  |  |  | (0.220) |  |
| *N* | 403 | |  |  | 260 | |  |  | 403 | |  |  | 260 | |
| *R^2^* | 0.21 | |  |  | 0.35 | |  |  | 0.03 | |  |  | 0.16 | |
| *Notes.* This table shows ordinary least squares (OLS) regression results predicting children's own-rated feasibility and originality of their selected ideas. For ethnicity, socio-economic status, and school site, the omitted category is the one with the most participants (Dutch, middle socio-economic status, school site #10). To test for heterogeneity of the treatment effect, interaction-analyses with demographic variables have been conducted as well. We found no heterogenous effects for demographics variables. Standard errors in parentheses. * *p* < 0.05, ** *p* < 0.01, *** *p* < 0.001. | | | | | | | | | | | | | | |

**S3 Table.** Regression estimated effect of expected implementation on feasibility and originality ratings (according to experts)

|  | **Feasibility ratings** | | | | | | | | | |  | |  | | **Originality ratings** | | | | | | | | | |
| --- | --- | --- | --- | --- | --- | --- | --- | --- | --- | --- | --- | --- | --- | --- | --- | --- | --- | --- | --- | --- | --- | --- | --- | --- |
|  | M1 | | |  | |  | | M2 | | |  | |  | | M1 | | |  | |  | | M2 | | |
|  | Main effect | | |  | |  | | Incl. interaction-analyses | | |  | |  | | Main effect | | |  | |  | | Incl.  interaction-analyses | | |
|  | B |  |  | |  | | B | |  |  | |  | | B | |  |  | |  | | B | |  |  |
|  | *(SE)* | *d* |  | |  | | *(SE)* | | *d* |  | |  | | *(SE)* | | *d* |  | |  | | *(SE)* | | *d* |  |
| Intercept | 2.570*** |  |  | |  | | 0.927 | |  |  | |  | | 4.134*** | |  |  | |  | | 5.882*** | |  |  |
|  | (0.076) |  |  | |  | | (1.498) | |  |  | |  | | (0.050) | |  |  | |  | | (1.364) | |  |  |
| Expected implementation | 0.584*** | 0.768 |  | |  | | 1.465 | | 2.389 |  | |  | | -0.207** | | 0.289 |  | |  | | -0.875 | | 0.984 |  |
|  | (0.076) |  |  | |  | | (1.065) | |  |  | |  | | (0.071) | |  |  | |  | | (0.969) | |  |  |
| **Demographic variables** |  |  |  | |  | |  | |  |  | |  | |  | |  |  | |  | |  | |  |  |
| Gender (ref.= boys) |  |  |  | |  | | 0.047 | |  |  | |  | |  | |  |  | |  | | -0.144 | |  |  |
|  |  |  |  | |  | | (0.113) | |  |  | |  | |  | |  |  | |  | | (0.103) | |  |  |
| Age (in years) |  |  |  | |  | | 0.044 | |  |  | |  | |  | |  |  | |  | | -0.101 | |  |  |
|  |  |  |  | |  | | (0.113) | |  |  | |  | |  | |  |  | |  | | (0.103) | |  |  |
| Ethnicity (ref. = Dutch) |  |  |  | |  | | -0.076 | |  |  | |  | |  | |  |  | |  | | -0.184 | |  |  |
|  |  |  |  | |  | | (0.343) | |  |  | |  | |  | |  |  | |  | | (0.312) | |  |  |
| Socio-economic status  (ref. = middle) |  |  |  | |  | |  | |  |  | |  | |  | |  |  | |  | |  | |  |  |
| Low |  |  |  | |  | | -0.021 | |  |  | |  | |  | |  |  | |  | | -0.139 | |  |  |
|  |  |  |  | |  | | (0.383) | |  |  | |  | |  | |  |  | |  | | (0.348) | |  |  |
| High |  |  |  | |  | | -0.082 | |  |  | |  | |  | |  |  | |  | | 0.350 | |  |  |
|  |  |  |  | |  | | (0.238) | |  |  | |  | |  | |  |  | |  | | (0.217) | |  |  |
| Report grade |  |  |  | |  | | 0.085 | |  |  | |  | |  | |  |  | |  | | 0.037 | |  |  |
|  |  |  |  | |  | | (0.057) | |  |  | |  | |  | |  |  | |  | | (0.052) | |  |  |
| School  (ref. = school site # 10) |  |  |  | |  | |  | |  |  | |  | |  | |  |  | |  | |  | |  |  |
| Site #1 |  |  |  | |  | | -0.721 | |  |  | |  | |  | |  |  | |  | | 0.372 | |  |  |
|  |  |  |  | |  | | (0.792) | |  |  | |  | |  | |  |  | |  | | (0.721) | |  |  |
| Site #2 |  |  |  | |  | | 1.095 | |  |  | |  | |  | |  |  | |  | | -0.579 | |  |  |
|  |  |  |  | |  | | (0.583) | |  |  | |  | |  | |  |  | |  | | (0.531) | |  |  |
| Site #3 |  |  |  | |  | | 0.079 | |  |  | |  | |  | |  |  | |  | | -0.202 | |  |  |
|  |  |  |  | |  | | (0.249) | |  |  | |  | |  | |  |  | |  | | (0.226) | |  |  |
| Site #4 |  |  |  | |  | | -0.131 | |  |  | |  | |  | |  |  | |  | | -0.146 | |  |  |
|  |  |  |  | |  | | (0.191) | |  |  | |  | |  | |  |  | |  | | (0.174) | |  |  |
| Site #5 |  |  |  | |  | | 0.062 | |  |  | |  | |  | |  |  | |  | | -0.109 | |  |  |
|  |  |  |  | |  | | (0.254) | |  |  | |  | |  | |  |  | |  | | (0.231) | |  |  |
| Site #6 |  |  |  | |  | | 0.050 | |  |  | |  | |  | |  |  | |  | | -0.161 | |  |  |
|  |  |  |  | |  | | (0.173) | |  |  | |  | |  | |  |  | |  | | (0.157) | |  |  |
| Site #7 |  |  |  | |  | | 0.373 | |  |  | |  | |  | |  |  | |  | | -0.080 | |  |  |
|  |  |  |  | |  | | (0.521) | |  |  | |  | |  | |  |  | |  | | (0.475) | |  |  |
| Site #8 |  |  |  | |  | | 0.217 | |  |  | |  | |  | |  |  | |  | | -0.364 | |  |  |
|  |  |  |  | |  | | (0.243) | |  |  | |  | |  | |  |  | |  | | (0.221) | |  |  |
| Site #9 |  |  |  | |  | | 0.636 | |  |  | |  | |  | |  |  | |  | | -0.710 | |  |  |
|  |  |  |  | |  | | (0.284) | |  |  | |  | |  | |  |  | |  | | (0.258) | |  |  |
| Site #11 |  |  |  | |  | | 0.431* | |  |  | |  | |  | |  |  | |  | | -0.242 | |  |  |
|  |  |  |  | |  | | (0.189) | |  |  | |  | |  | |  |  | |  | | (0.172) | |  |  |
| Site #12 |  |  |  | |  | | 0.268 | |  |  | |  | |  | |  |  | |  | | -0.323 | |  |  |
|  |  |  |  | |  | | (0.279) | |  |  | |  | |  | |  |  | |  | | (0.254) | |  |  |
| Site #13 |  |  |  | |  | | 0.148 | |  |  | |  | |  | |  |  | |  | | -0.024 | |  |  |
|  |  |  |  | |  | | (0.275) | |  |  | |  | |  | |  |  | |  | | (0.251) | |  |  |
| **Psychological variables** |  |  |  | |  | |  | |  |  | |  | |  | |  |  | |  | |  | |  |  |
| Risk preference |  |  |  | |  | | 0.083 | |  |  | |  | |  | |  |  | |  | | -0.175 | |  |  |
|  |  |  |  | |  | | (0.116) | |  |  | |  | |  | |  |  | |  | | (0.106) | |  |  |
| Personality traits |  |  |  | |  | |  | |  |  | |  | |  | |  |  | |  | |  | |  |  |
| Openness to experience |  |  |  | |  | | -0.089 | |  |  | |  | |  | |  |  | |  | | 0.069 | |  |  |
|  |  |  |  | |  | | (0.118) | |  |  | |  | |  | |  |  | |  | | (0.108) | |  |  |
| Conscientiousness |  |  |  | |  | | 0.243 | |  |  | |  | |  | |  |  | |  | | -0.280 | |  |  |
|  |  |  |  | |  | | (0.121) | |  |  | |  | |  | |  |  | |  | | (0.111) | |  |  |
| Extraversion |  |  |  | |  | | -0.046 | |  |  | |  | |  | |  |  | |  | | 0.152 | |  |  |
|  |  |  |  | |  | | (0.127) | |  |  | |  | |  | |  |  | |  | | (0.116) | |  |  |
| Agreeableness |  |  |  | |  | | -0.047 | |  |  | |  | |  | |  |  | |  | | 0.027 | |  |  |
|  |  |  |  | |  | | (0.143) | |  |  | |  | |  | |  |  | |  | | (0.131) | |  |  |
| Emotional stability |  |  |  | |  | | -0.084 | |  |  | |  | |  | |  |  | |  | | 0.031 | |  |  |
|  |  |  |  | |  | | (0.104) | |  |  | |  | |  | |  |  | |  | | (0.095) | |  |  |
| **Interaction with psychological variables** |  |  |  | |  | |  | |  |  | |  | |  | |  |  | |  | |  | |  |  |
| Implementation*  risk preference |  |  |  | |  | | -0.180 | |  |  | |  | |  | |  |  | |  | | 0.278 | |  |  |
|  |  |  |  | |  | | (0.162) | |  |  | |  | |  | |  |  | |  | | (0.148) | |  |  |
| Implementation*  openness |  |  |  | |  | | -0.053 | |  |  | |  | |  | |  |  | |  | | 0.163 | |  |  |
|  |  |  |  | |  | | (0.182) | |  |  | |  | |  | |  |  | |  | | (0.166) | |  |  |
| Implementation*  conscientiousness |  |  |  | |  | | -0.317 | |  |  | |  | |  | |  |  | |  | | 0.355 | |  |  |
|  |  |  |  | |  | | (0.168) | |  |  | |  | |  | |  |  | |  | | (0.152) | |  |  |
| Implementation*  extraversion |  |  |  | |  | | 0.232 | |  |  | |  | |  | |  |  | |  | | -0.507 | |  |  |
|  |  |  |  | |  | | (0.179) | |  |  | |  | |  | |  |  | |  | | (0.163) | |  |  |
| Implementation*  agreeableness |  |  |  | |  | | -0.046 | |  |  | |  | |  | |  |  | |  | | -0.082 | |  |  |
|  |  |  |  | |  | | (0.208) | |  |  | |  | |  | |  |  | |  | | (0.189) | |  |  |
| Implementation*  emotional stability |  |  |  | |  | | 0.143 | |  |  | |  | |  | |  |  | |  | | -0.029 | |  |  |
|  |  |  |  | |  | | (0.146) | |  |  | |  | |  | |  |  | |  | | (0.133) | |  |  |
| *N* | 403 | | |  | |  | | 260 | | |  | |  | | 403 | | |  | |  | | 260 | | |
| *R^2^* | 0.13 | | |  | |  | | 0.27 | | |  | |  | | 0.02 | | |  | |  | | 0.21 | | |
| *Notes.* This table shows ordinary least squares (OLS) regression results predicting children's own-rated feasibility and originality of their selected ideas. For ethnicity, socio-economic status, and school site, the omitted category is the one with the most participants (Dutch, middle socio-economic status, school site #10). Standard errors in parentheses. * *p* < 0.05, ** *p* < 0.01, *** *p* < 0.001. | | | | | | | | | | | | | | | | | | | | | | | | |

**Figures**


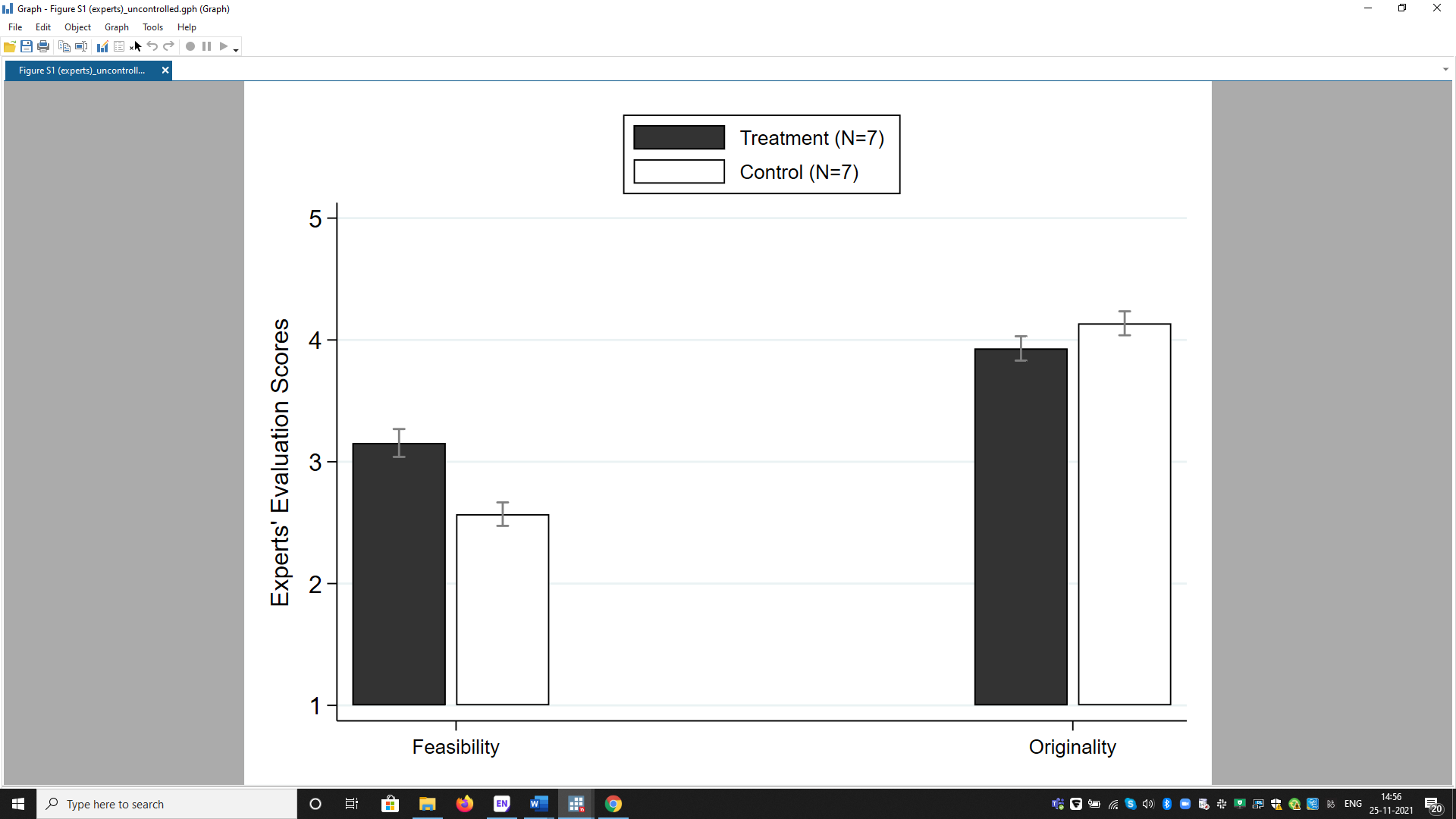


**S1 Figure***.* Estimated effects of expected implementation on experts’ feasibility and originality ratings. Error bars represent 1 SE.

*Notes*. This figure summarizes the intervention’s effect on children’s feasibility and originality ratings according to experts. Error bars reflect 95% confidence intervals.

**S1 Appendix.** Questionnaire with randomized field experiment

This appendix contains screenshots of each page of the second questionnaire, in which the randomized field experiment was conducted. Some pages, as noted, were displayed only to participants in the treatment or control condition.

**Intro (presented to all participants)**


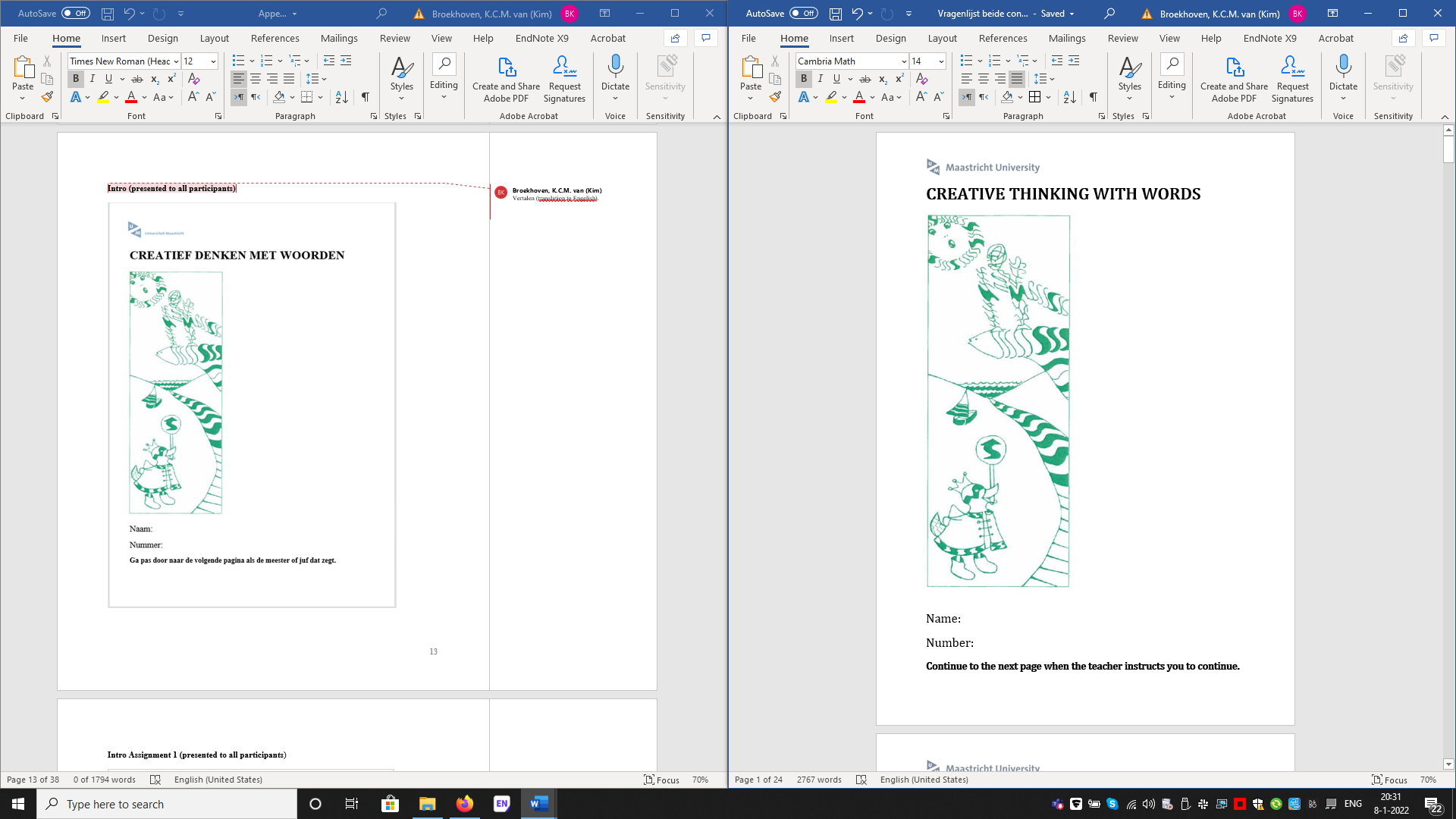


**Intro Assignment 1 (presented to all participants**)


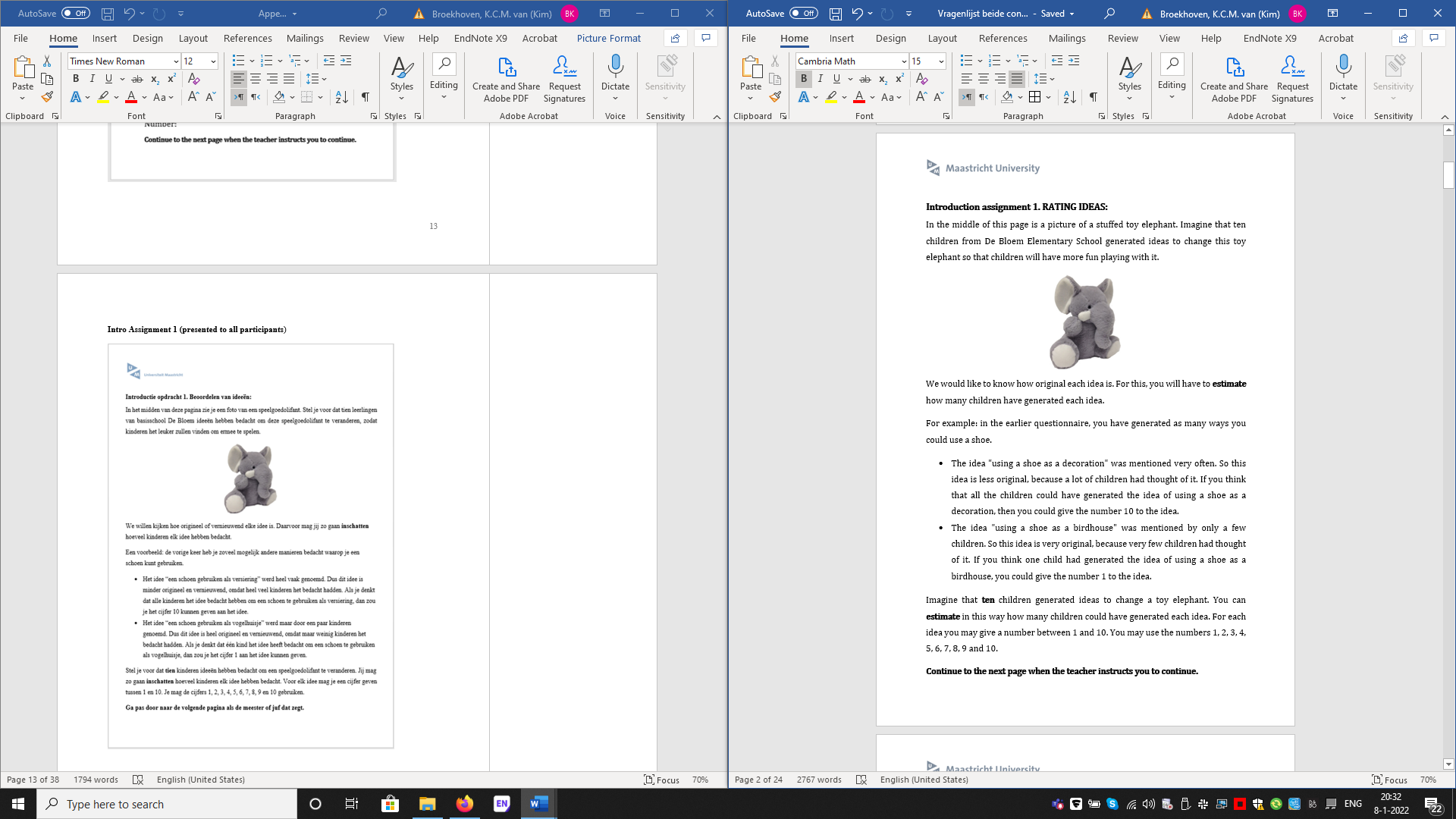


**Practice Assignment (presented to all participants**)


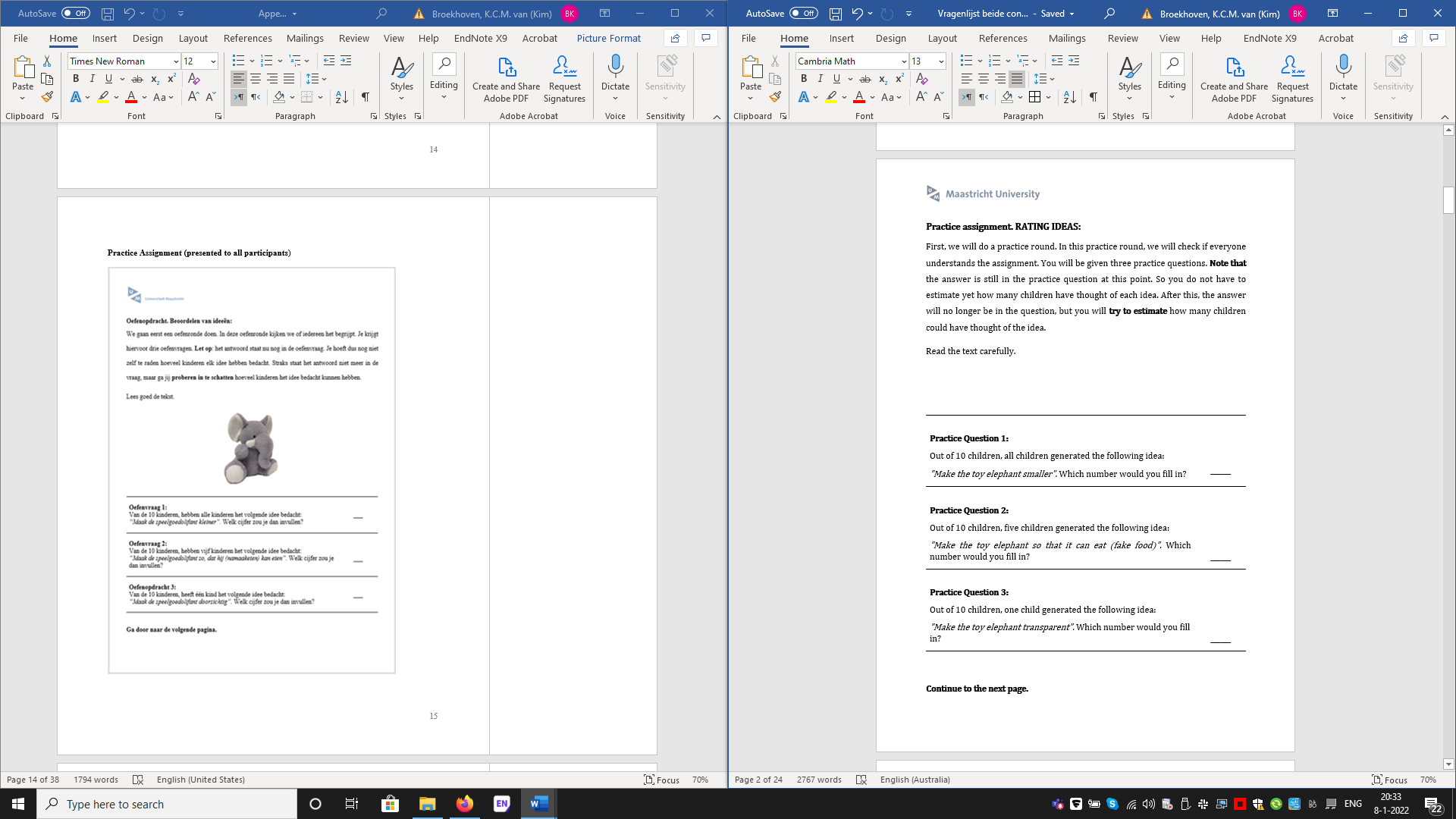


**Reading Assignment (presented to all participants**)


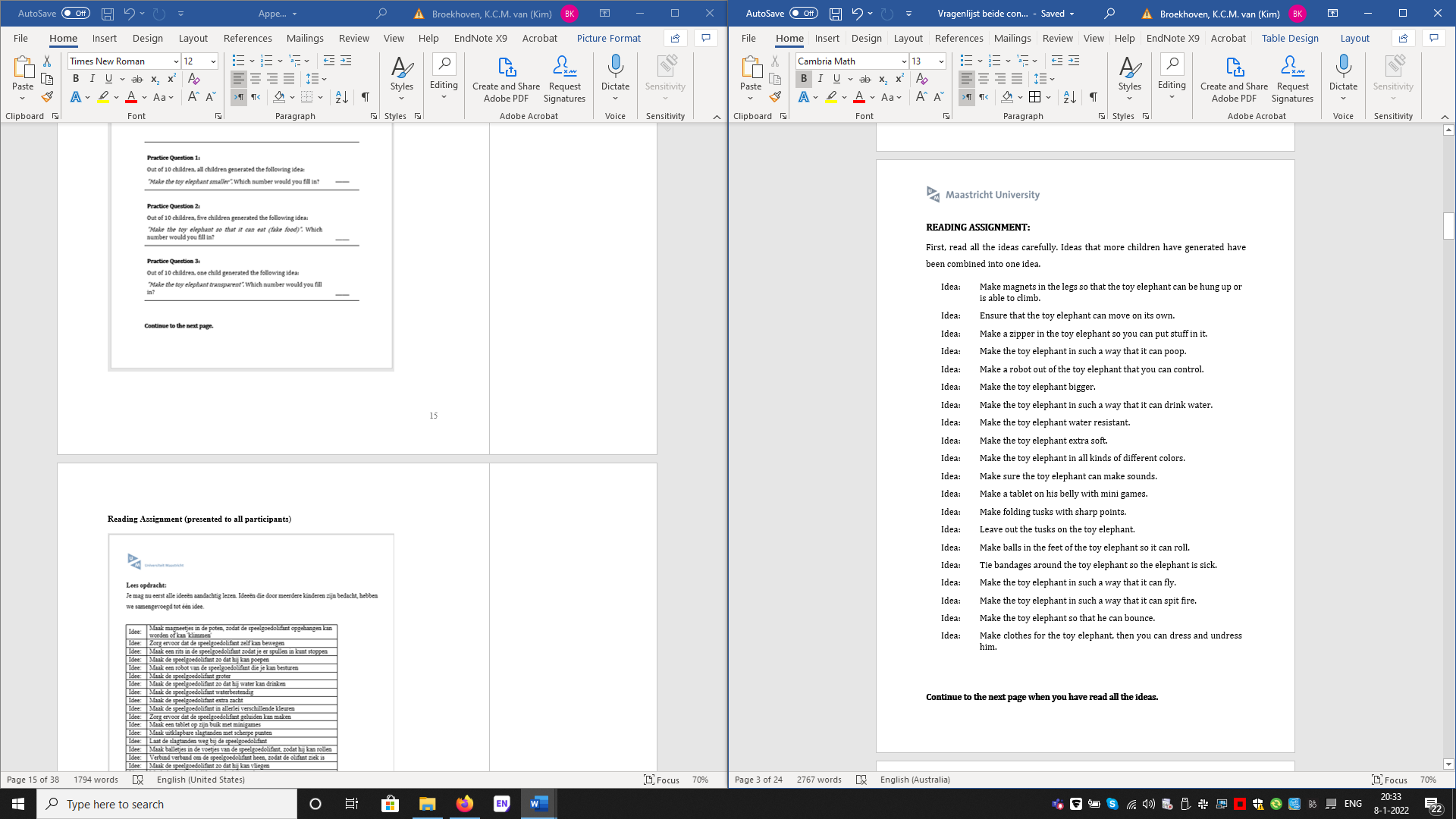


**Assignment 1 (presented to all participants**)


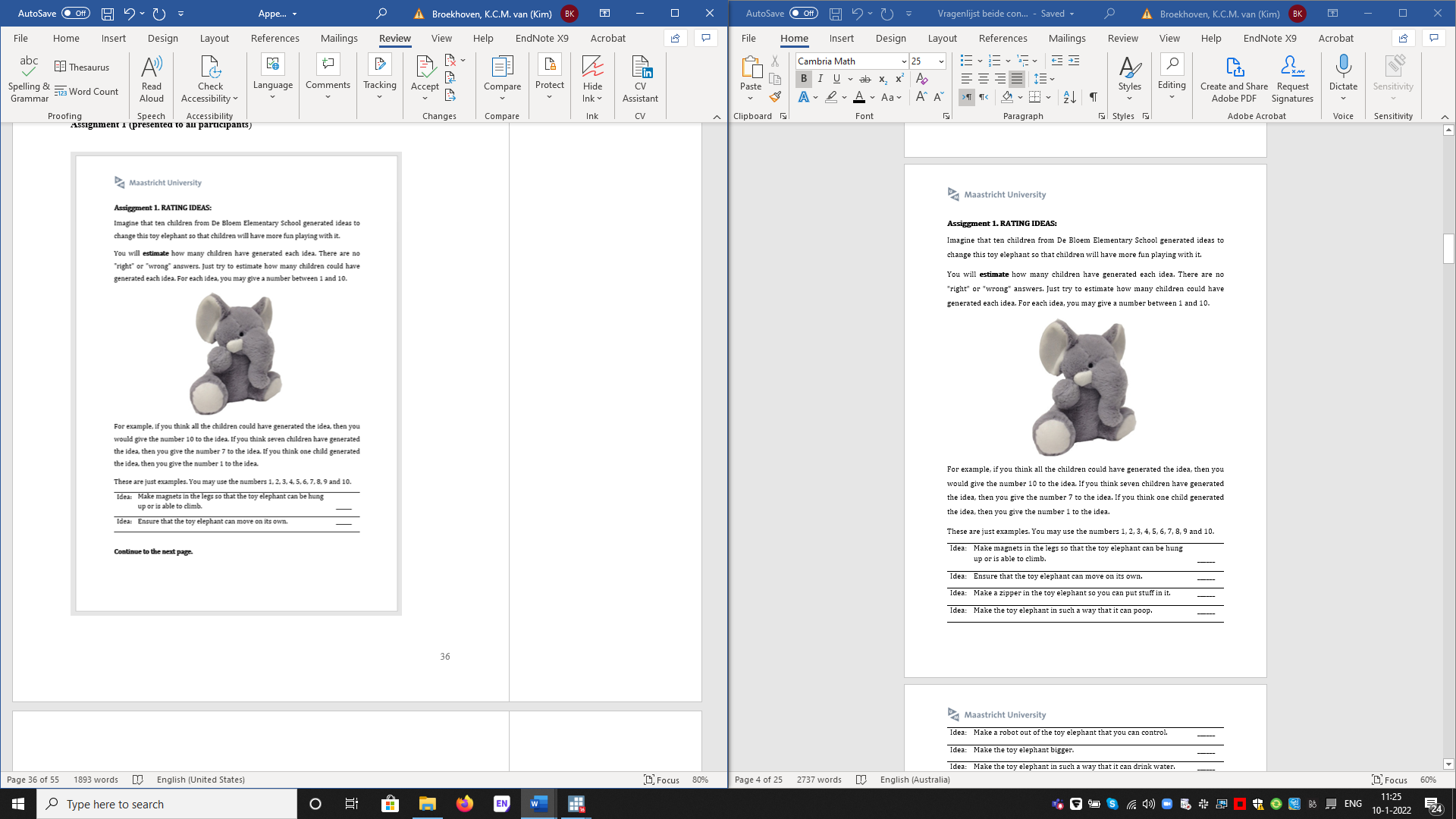


**Continuation assignment 1 (presented to all participants**)


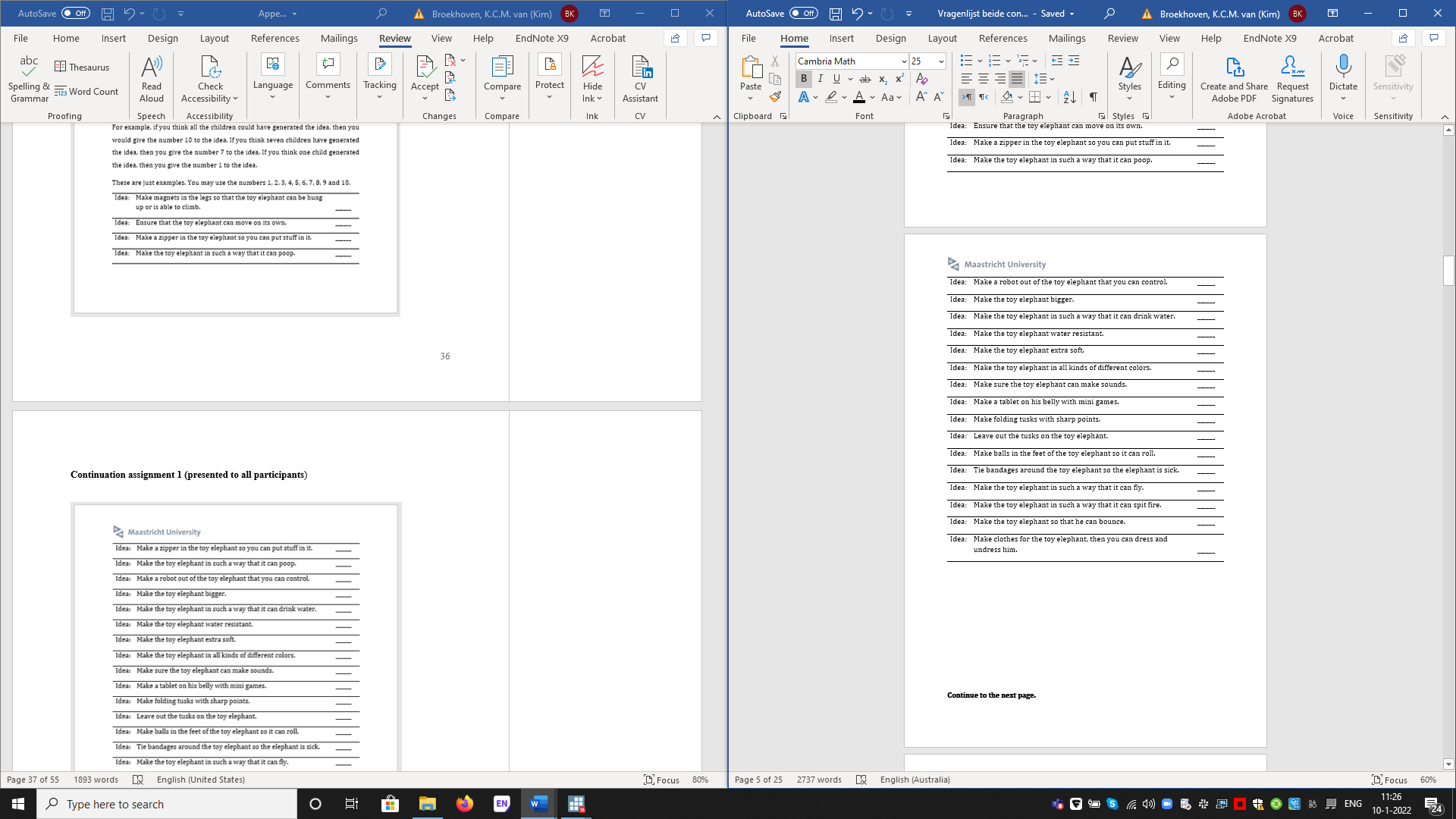


**Assignment 2 (presented to all participants**)


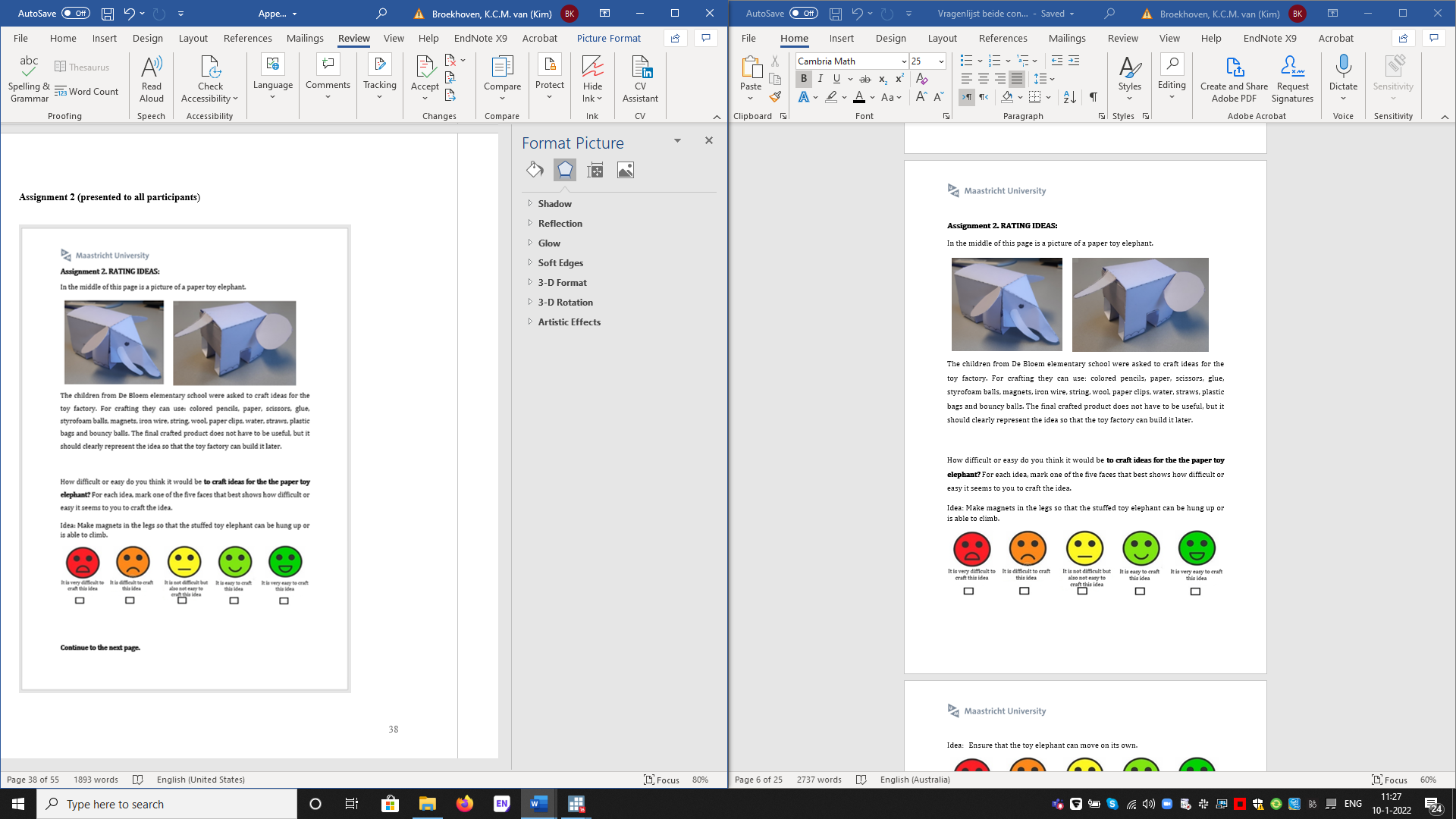


**Continuation assignment 2 (presented to all participants**)


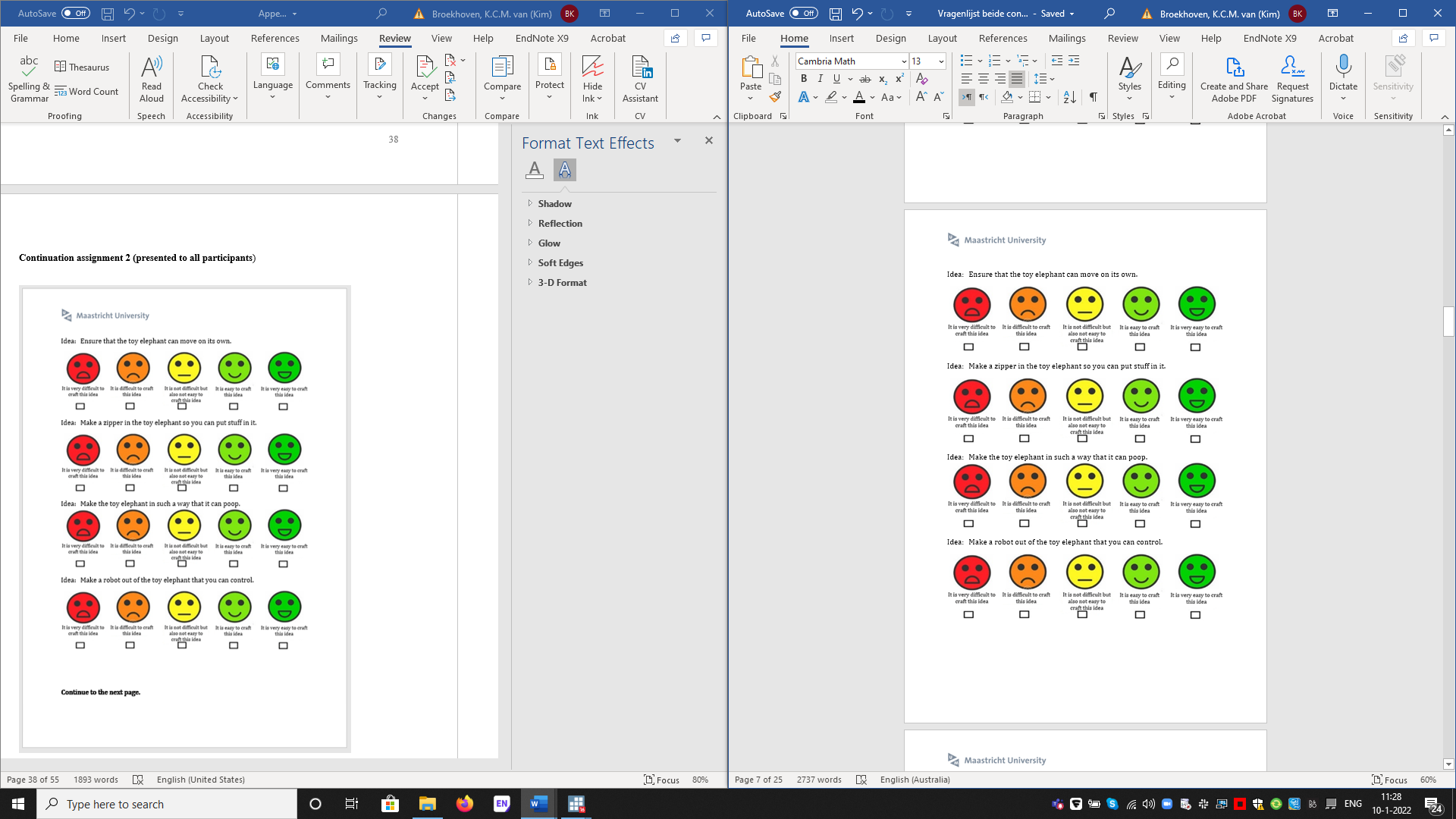


**Continuation assignment 2 (presented to all participants**)


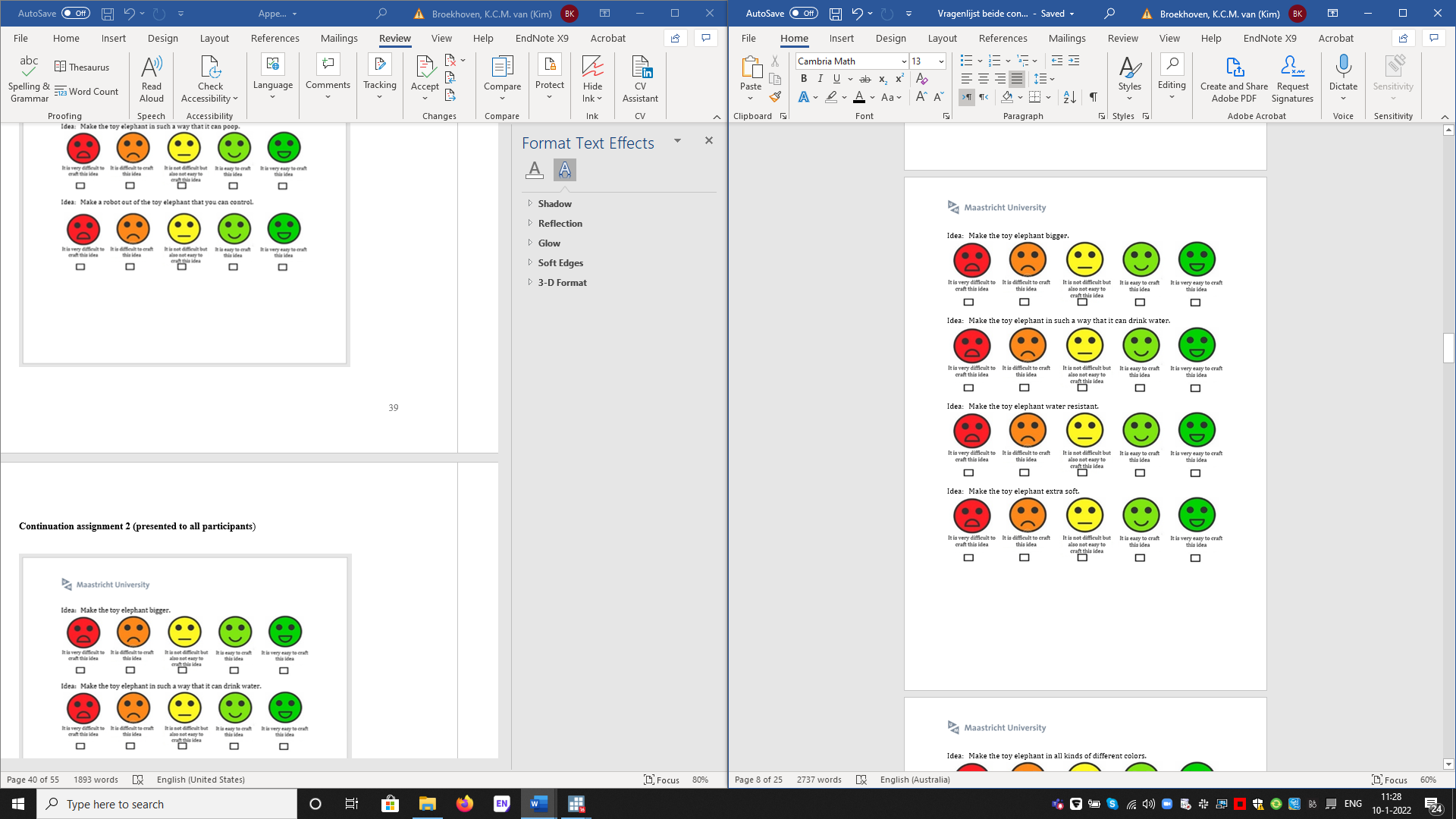


**Continuation assignment 2 (presented to all participants**)


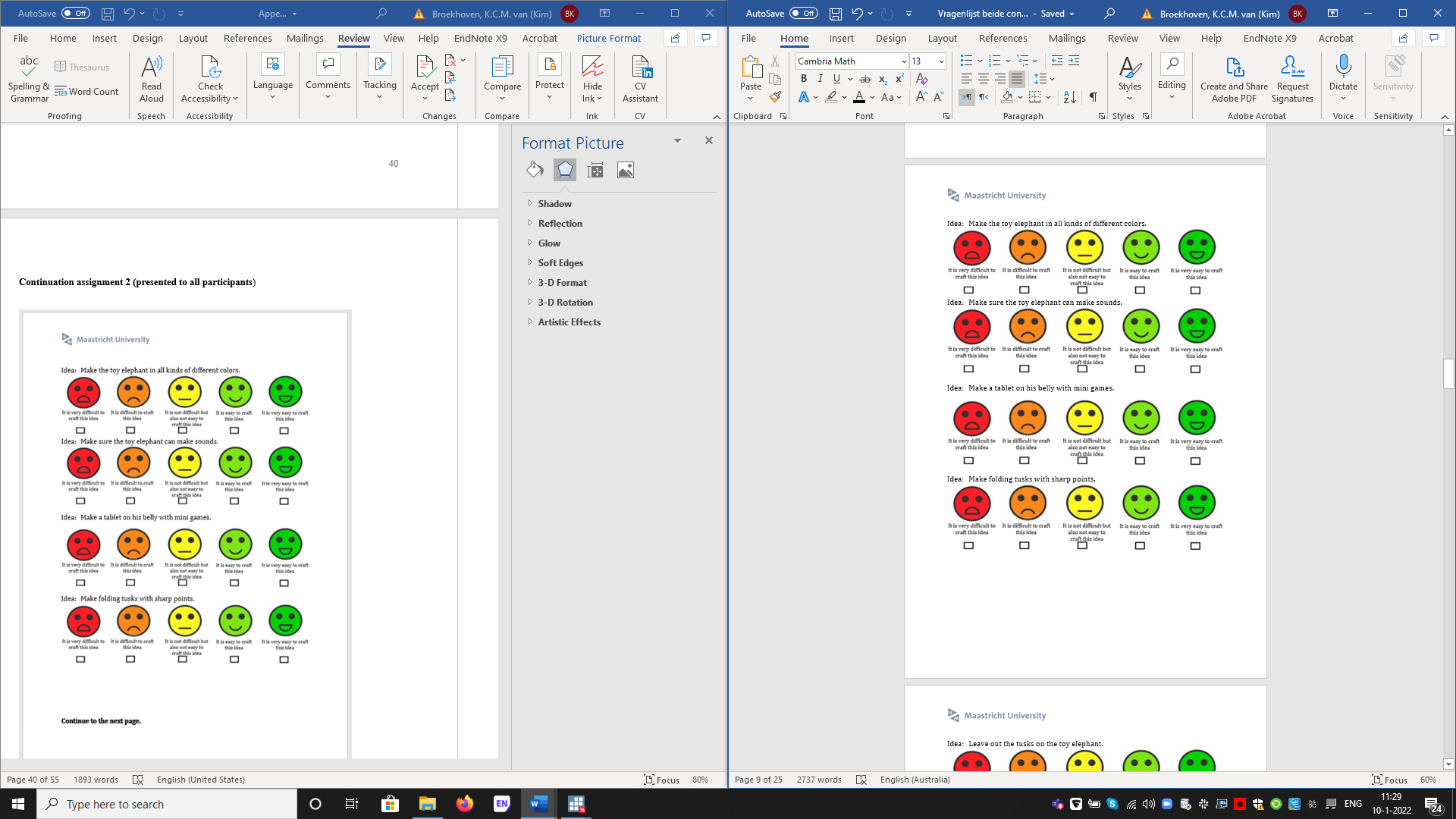


**Continuation assignment 2 (presented to all participants**)


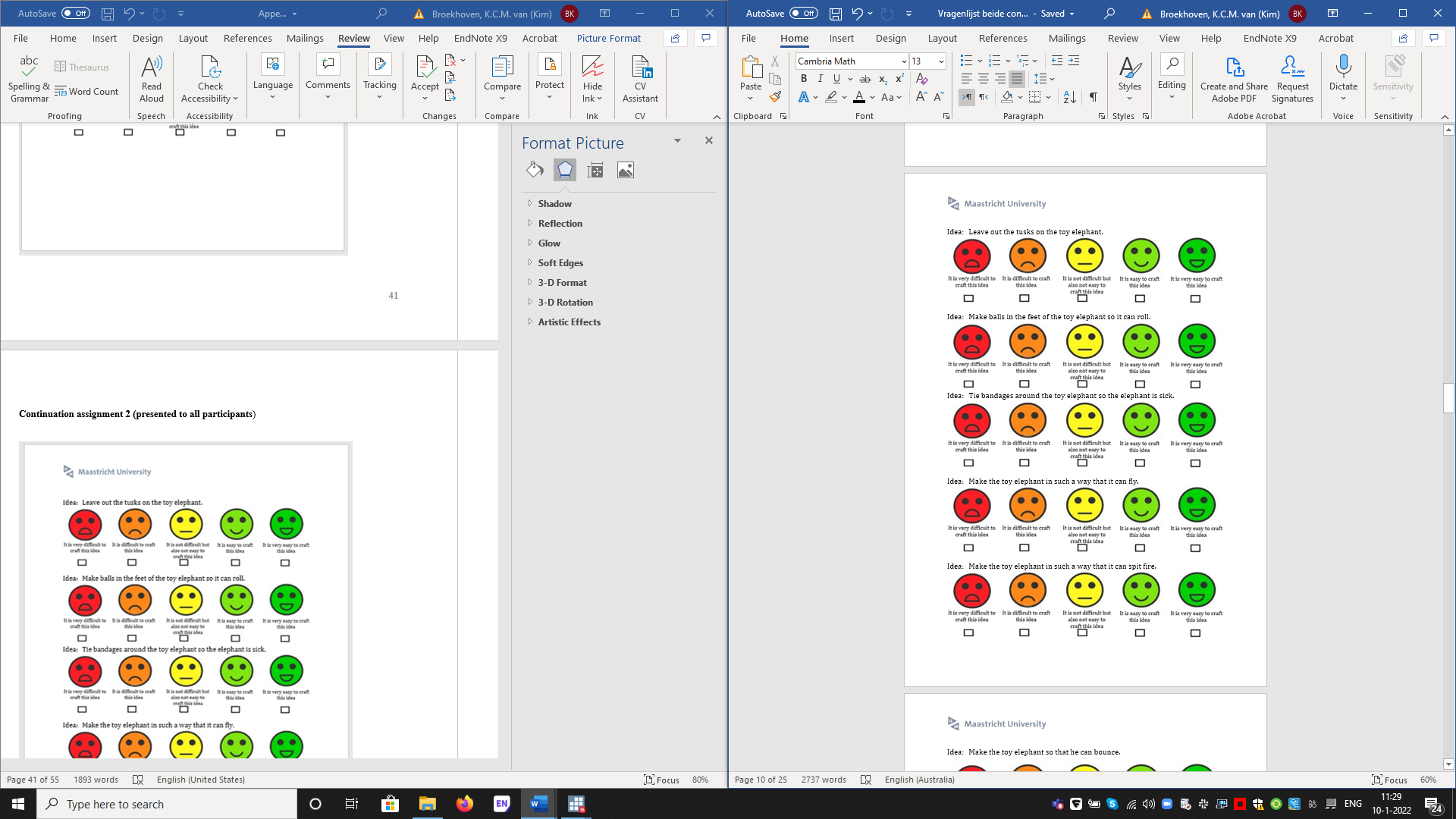


**Continuation assignment 2 (presented to all participants**)


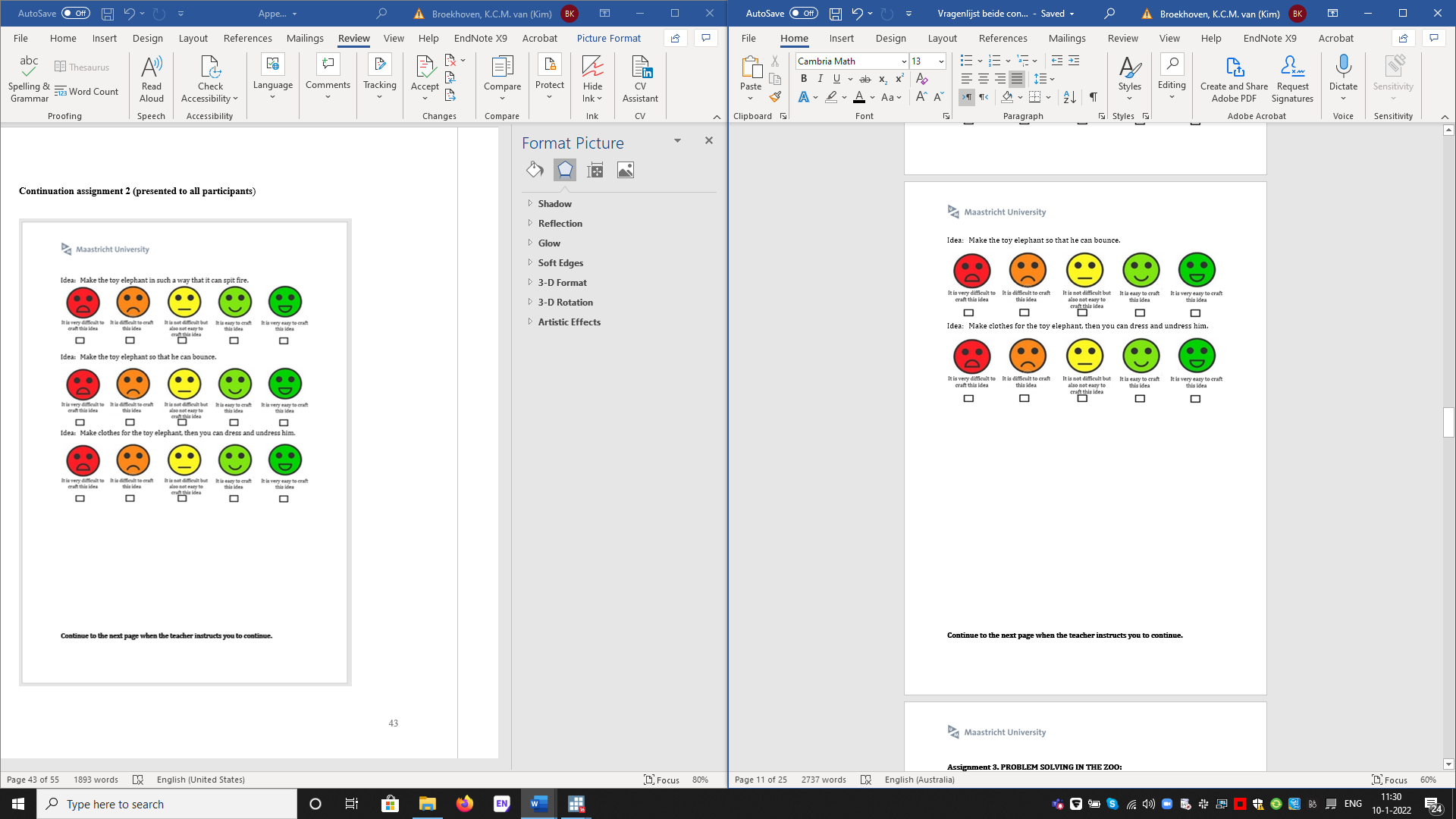


**Assignment 3 (presented to all participants**)


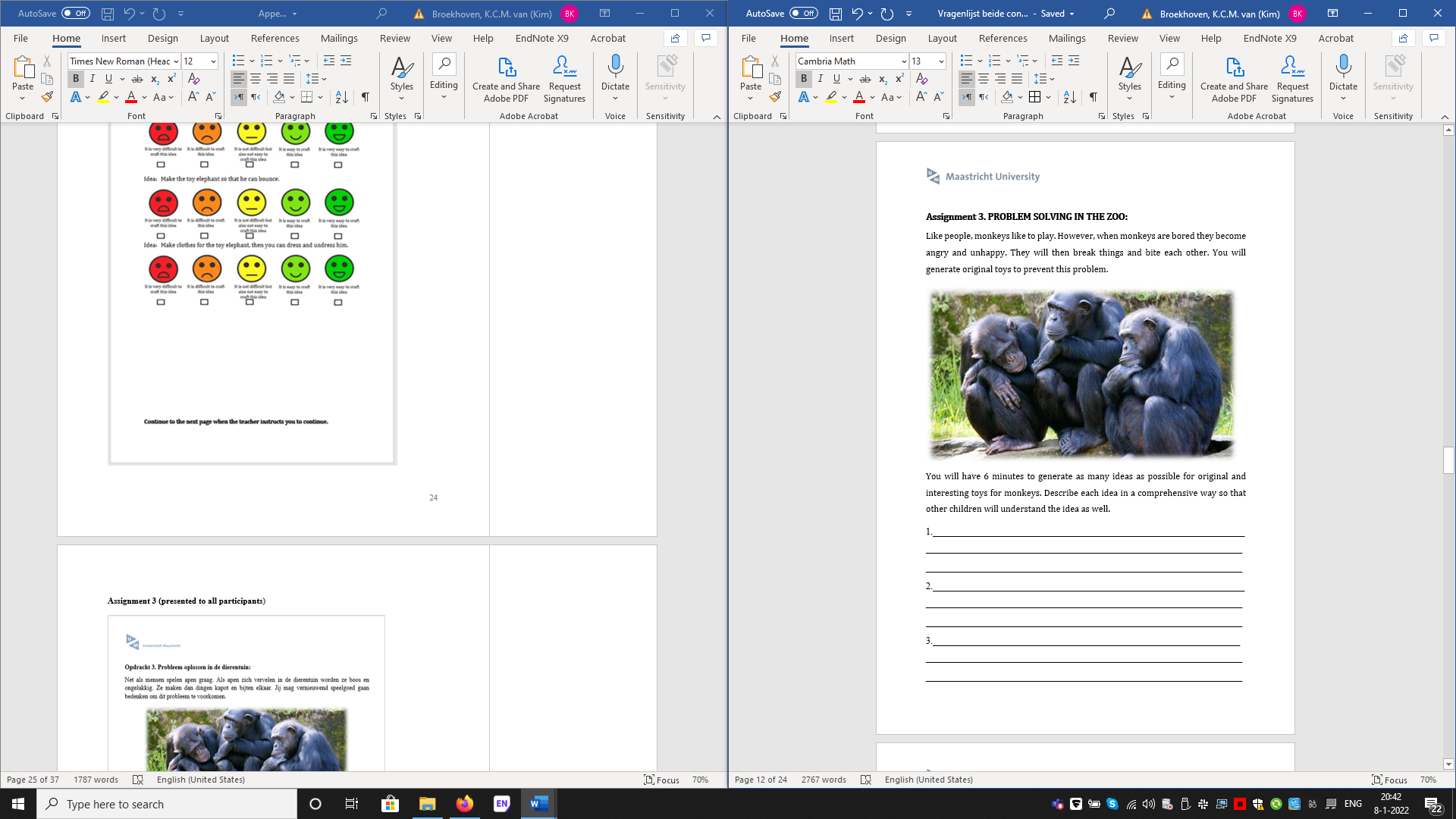


**Continuation assignment 3 (presented to all participants**)


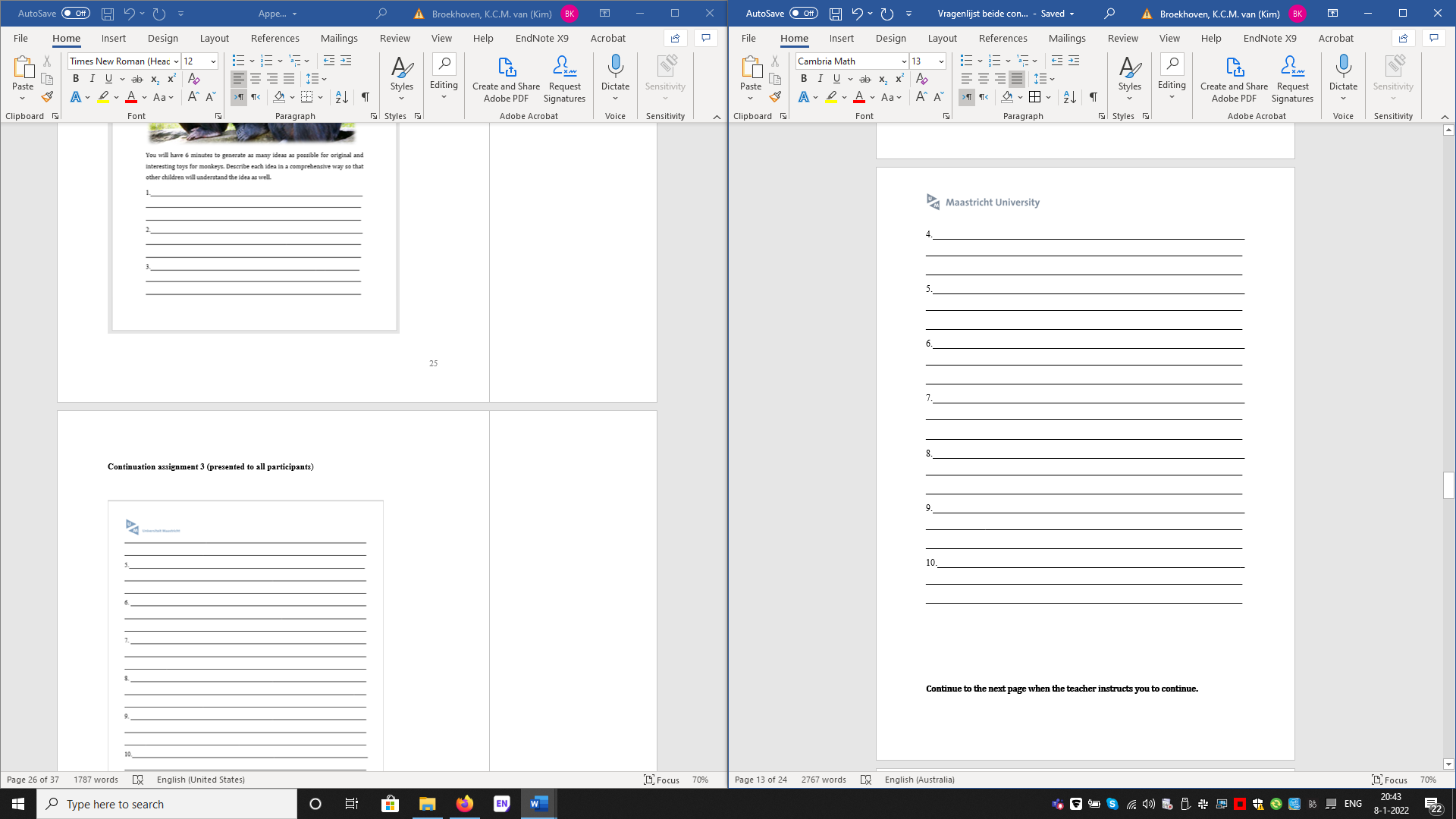


**Expected Implementation Treatment Condition** (Screenshot of p. 1)


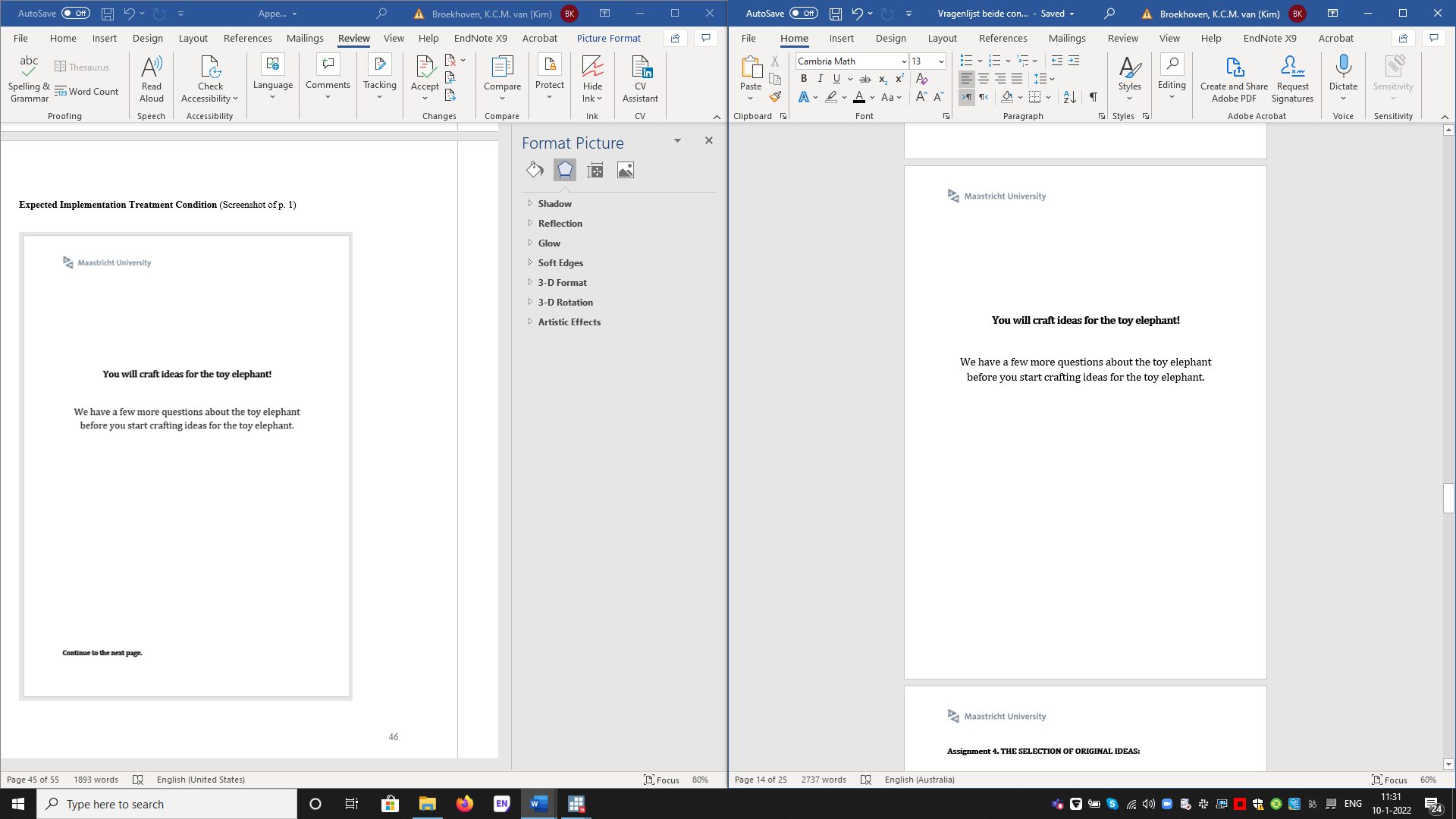


**Expected Implementation Treatment Condition** (Screenshot of p. 2)


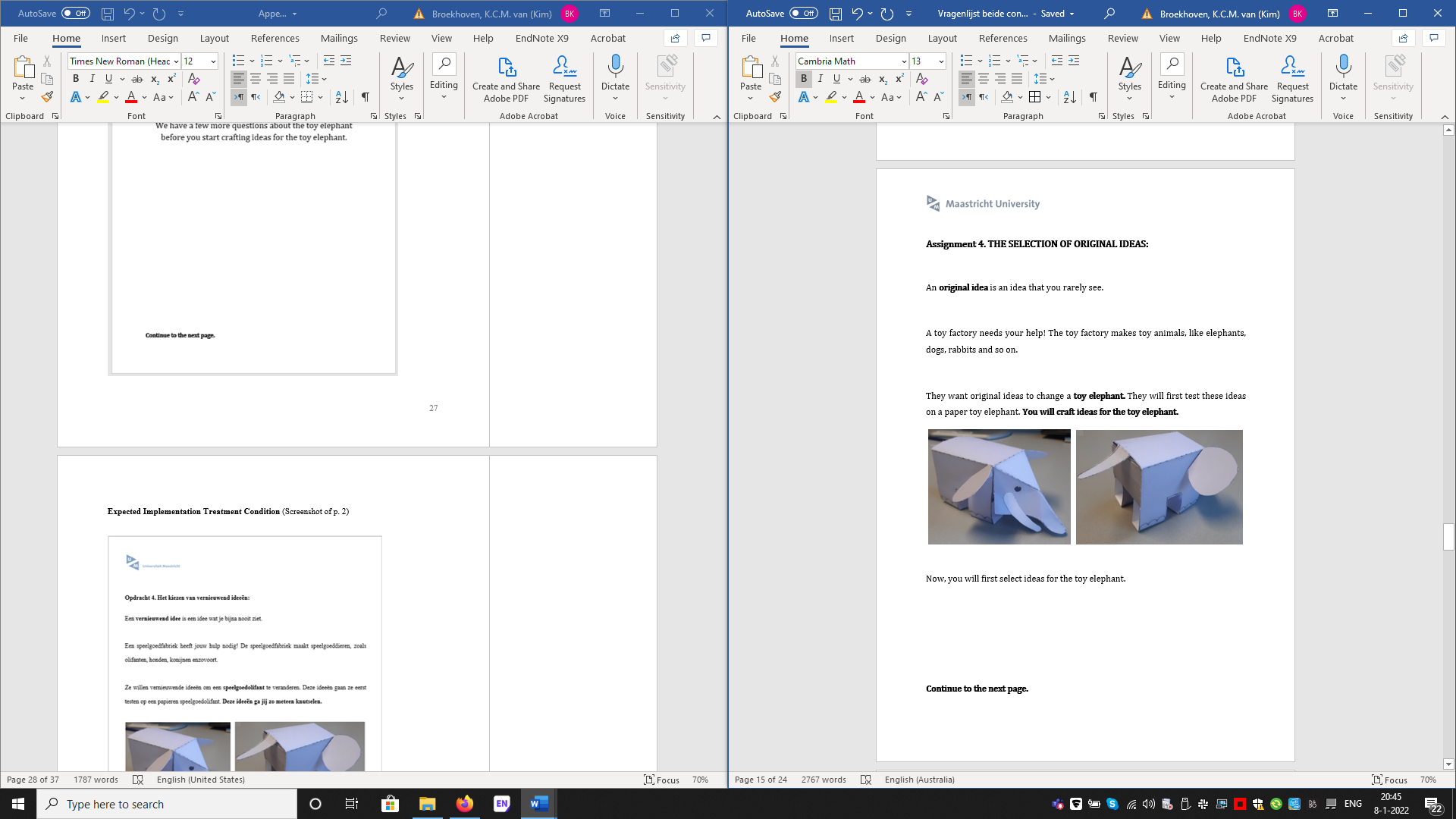


**Expected Implementation Treatment Condition** (Screenshot of p. 3)


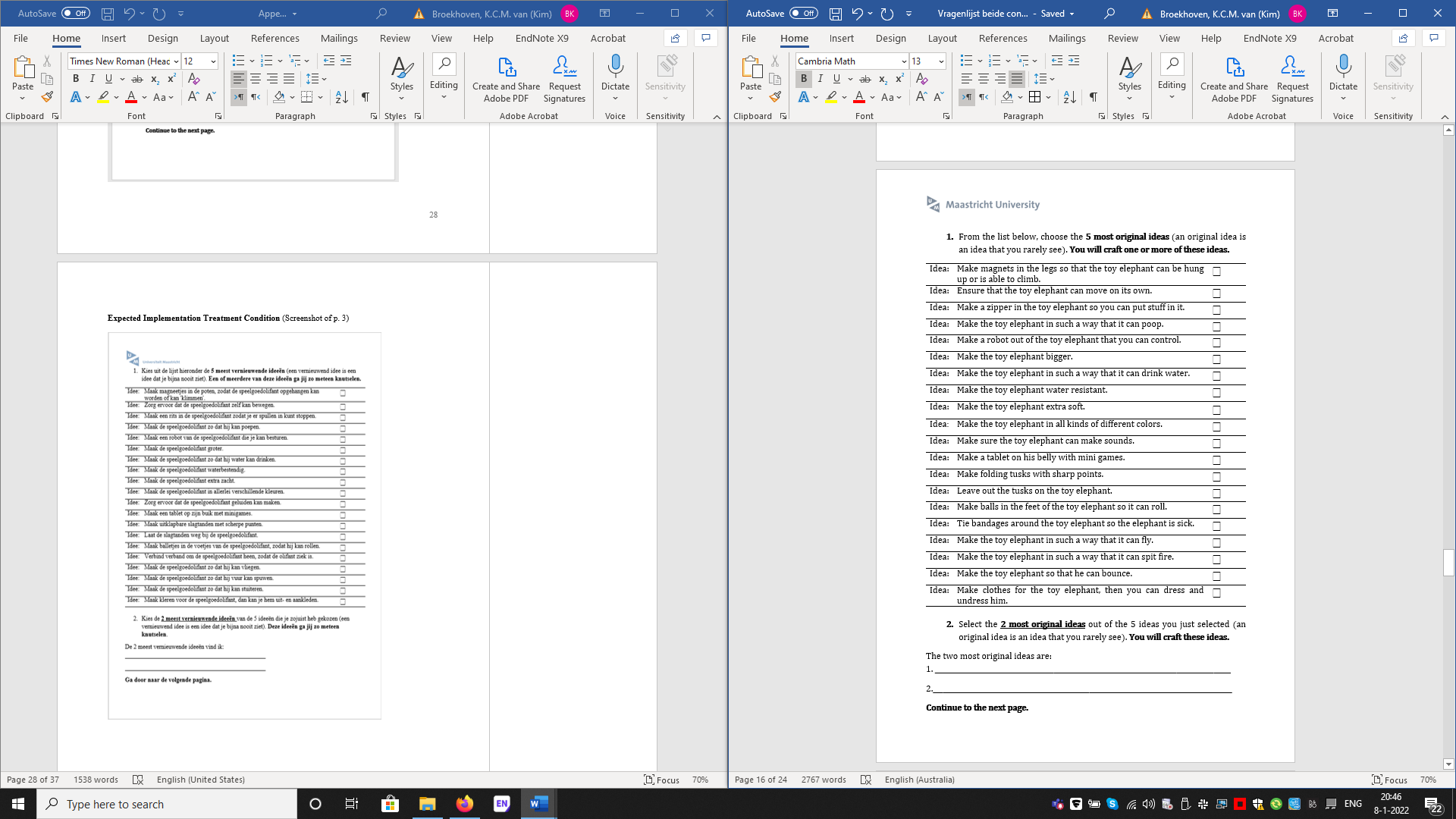


**Non-Expected Implementation Control Condition** (Screenshot of p. 1)


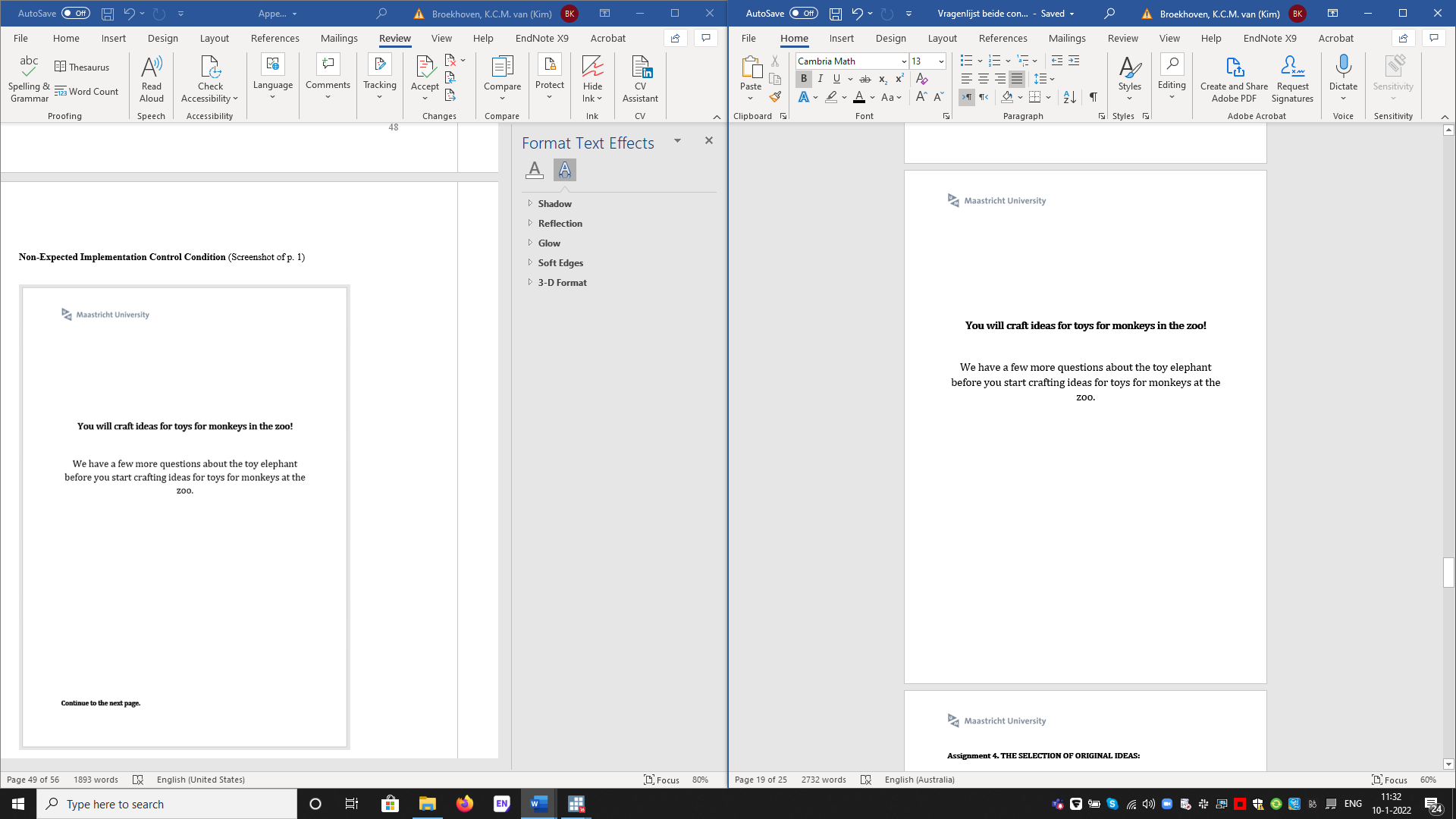


**Non-Expected Implementation Control Condition** (Screenshot of p. 2)


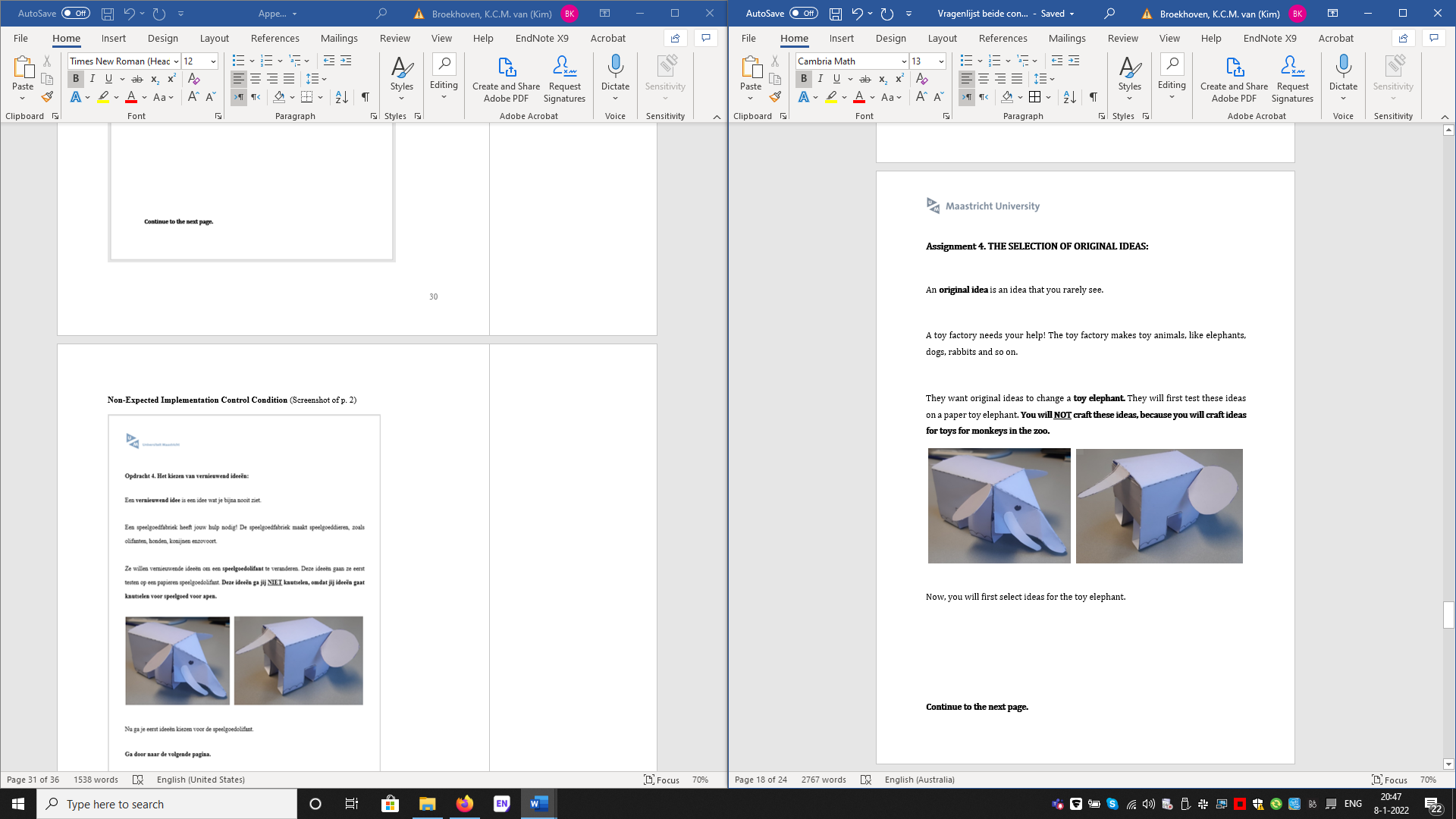


**Non-Expected Implementation Control Condition** (Screenshot of p. 3)


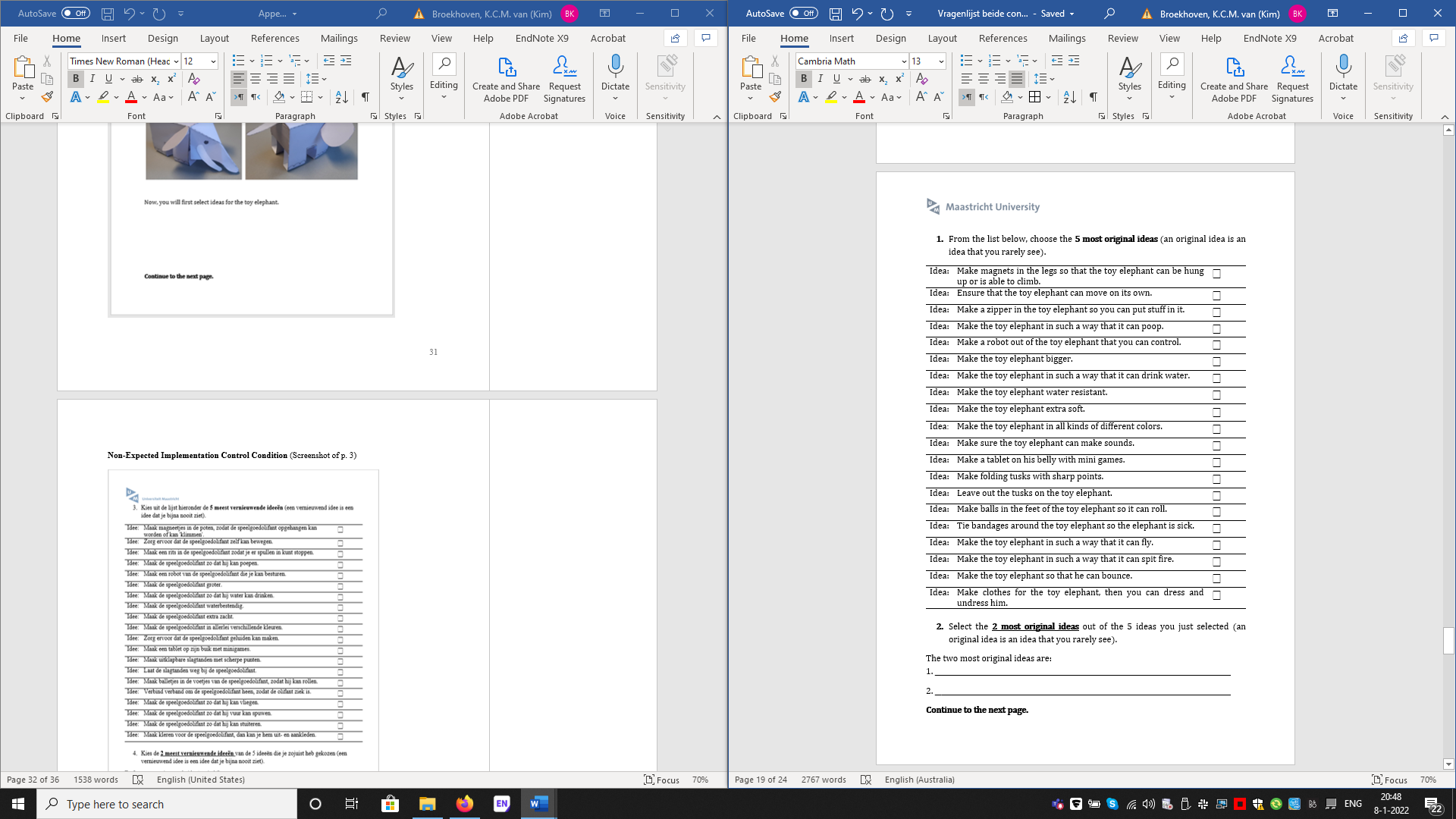


**Manipulation Check (presented to all participants;** Screenshot of p. 4 for **Expected Implementation Treatment condition;** screenshot of p. 4 for **Non-Expected Implementation** **Control Condition**)


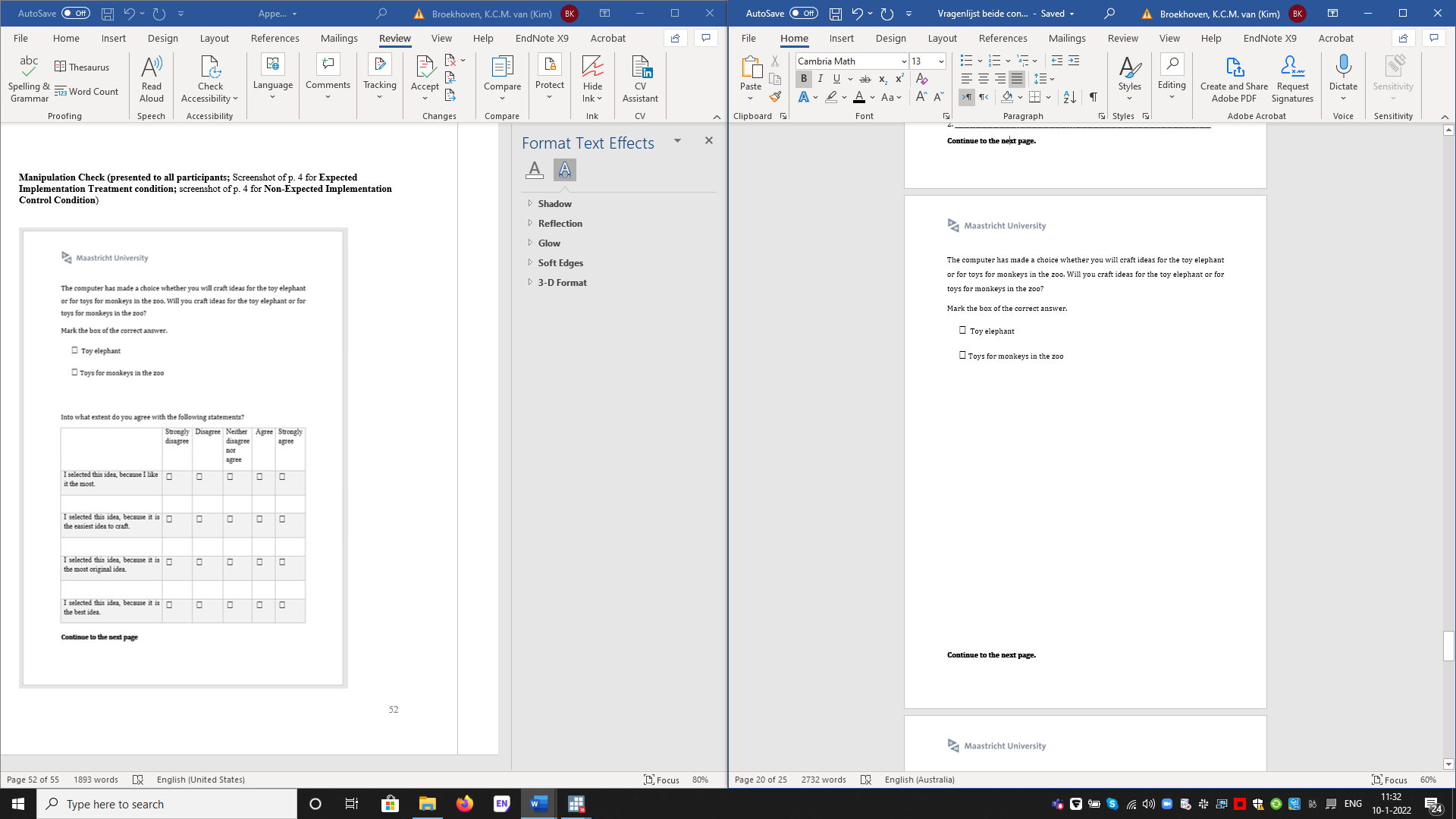


**Additional assignment Non-Expected Control Condition** (Screenshot of p. 5)


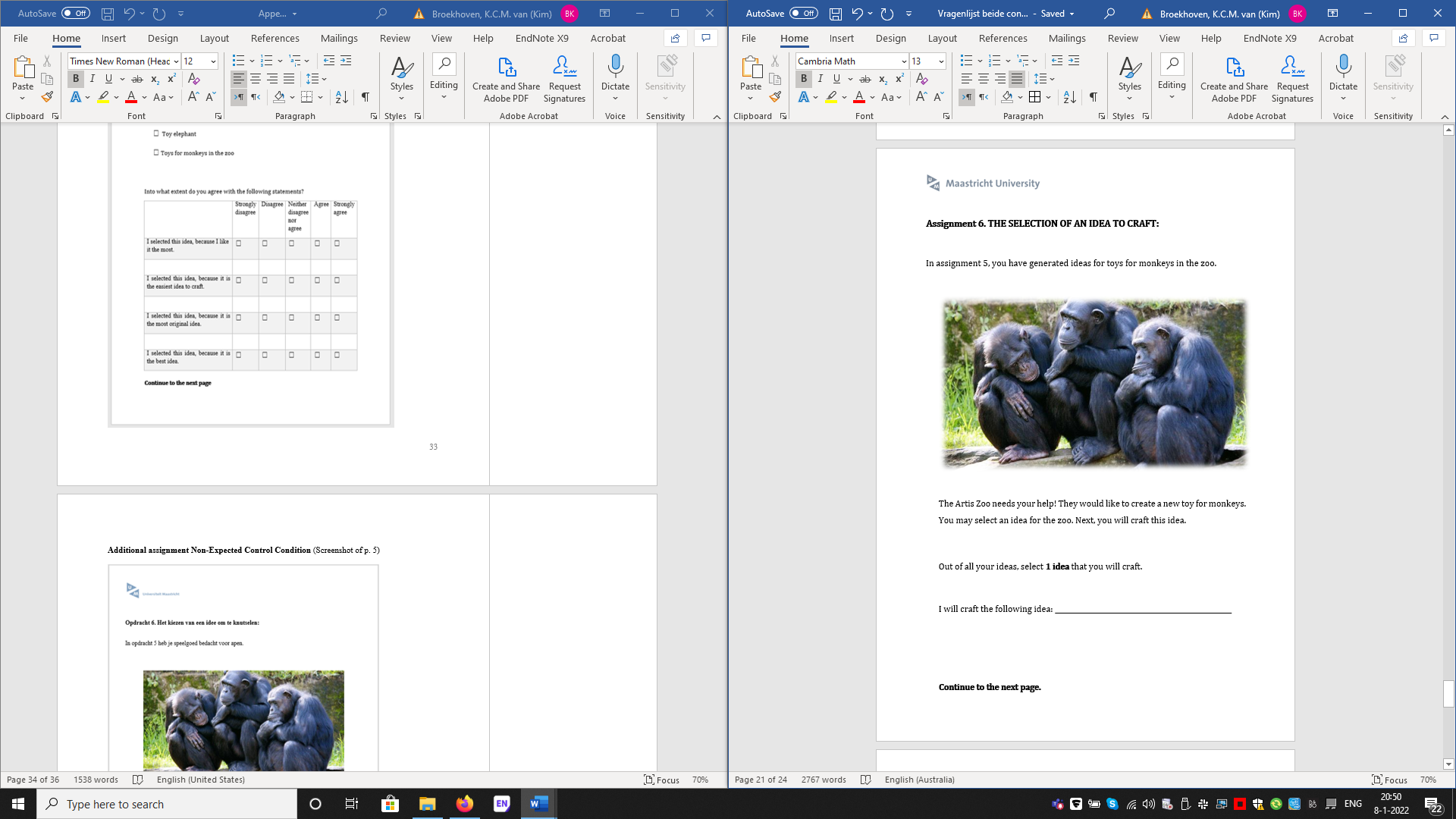


**End Expected Implementation Treatment Condition** (Screenshot of p. 5)


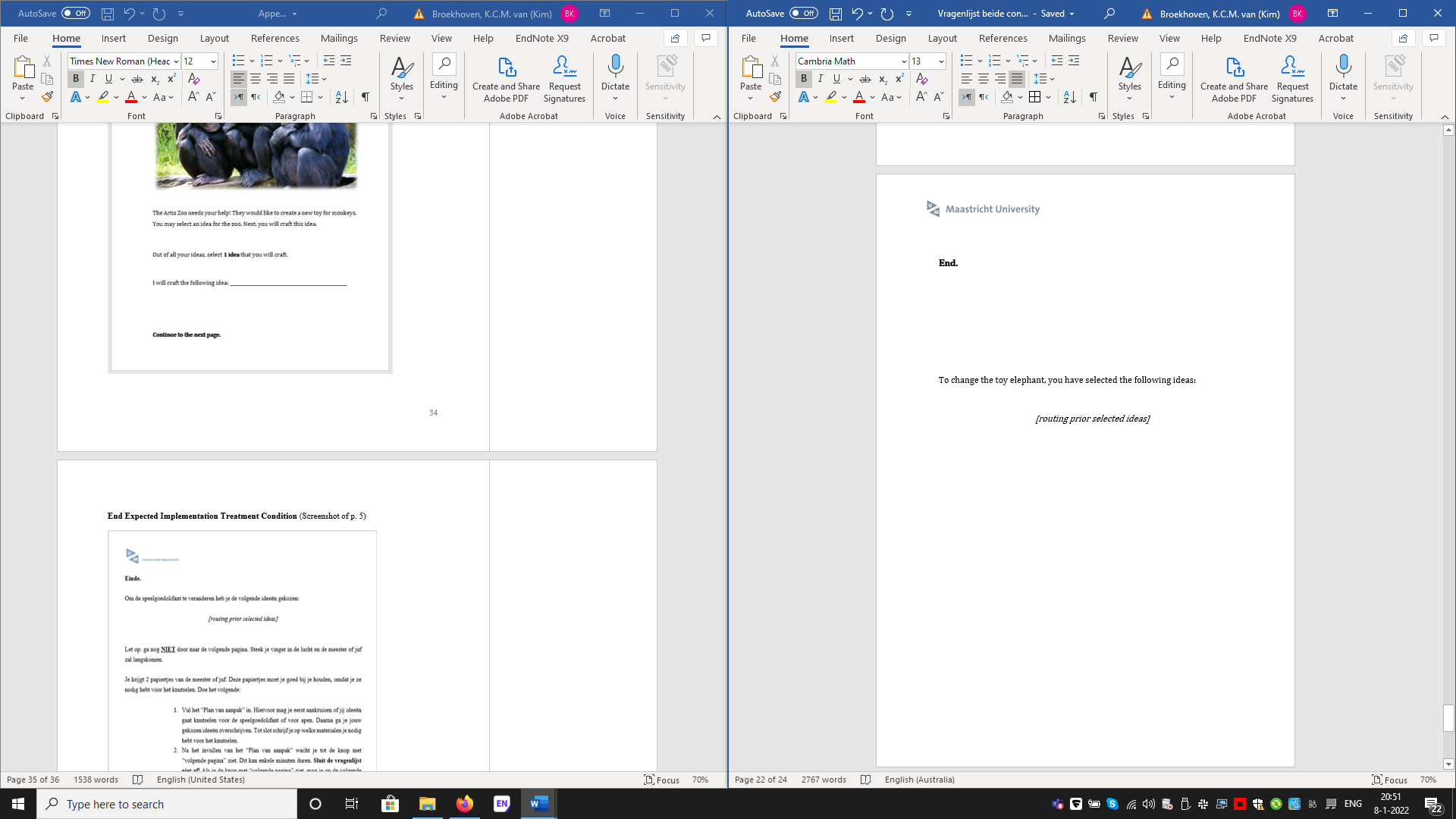


**End Non-Expected Implementation Control Condition** (Screenshot of p. 6)


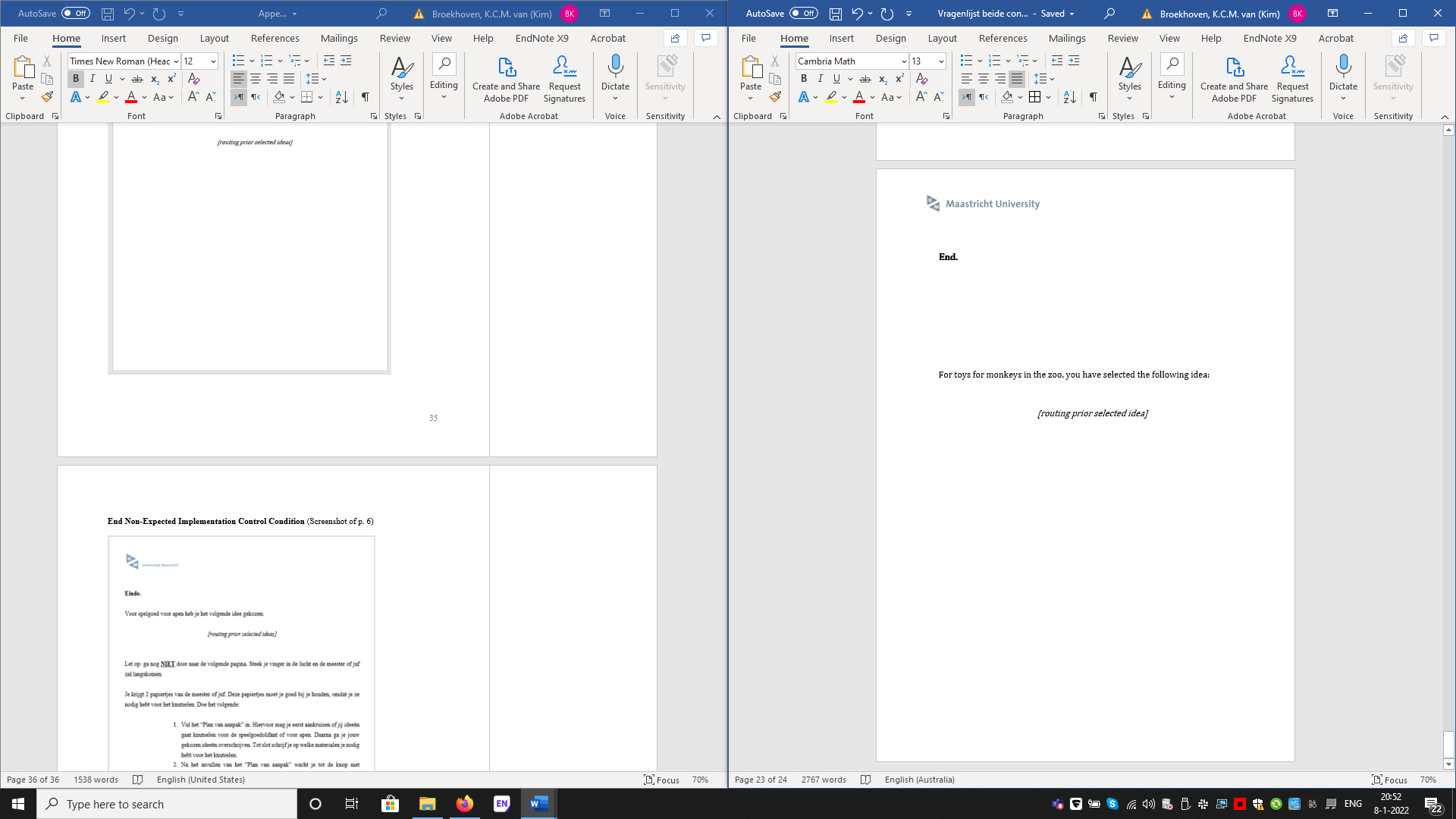


**S2 Appendix..** List of pre-defined ideas

This appendix contains the list of twenty pre-defined ideas to improve a stuffed toy elephant

| 1 | Make magnets in the legs so that the toy elephant can be hung up or is able to climb. |
| --- | --- |
| 2 | Ensure that the toy elephant can move on its own. |
| 3 | Make a zipper in the toy elephant so you can put stuff in it. |
| 4 | Make the toy elephant in such a way that it can poop. |
| 5 | Make a robot out of the toy elephant that you can control. |
| 6 | Make the toy elephant bigger. |
| 7 | Make the toy elephant in such a way that it can drink water. |
| 8 | Make the toy elephant water resistant. |
| 9 | Make the toy elephant extra soft. |
| 10 | Make the toy elephant in all kinds of different colors. |
| 11 | Make sure the toy elephant can make sounds. |
| 12 | Make a tablet on his belly with mini games. |
| 13 | Make folding tusks with sharp points. |
| 14 | Leave out the tusks on the toy elephant. |
| 15 | Make balls in the feet of the toy elephant so it can roll. |
| 16 | Tie bandages around the toy elephant so the elephant is sick. |
| 17 | Make the toy elephant in such a way that it can fly. |
| 18 | Make the toy elephant in such a way that it can spit fire. |
| 19 | Make the toy elephant so that he can bounce. |
| 20 | Make clothes for the toy elephant, then you can dress and undress him. |

**Idea (originality low/high) x 2 (feasibility low/high) Matrix**

|  | **Low feasibility** | **High feasibility** |
| --- | --- | --- |
| **High originality** | *Make the toy elephant in such a way that it can drink water*  *Make the toy elephant in such a way that it can poop*  *Make a robot out of the toy elephant that you can control*  *Make the toy elephant in such a way that it can fly*  *Make the toy elephant in such a way that it can spit fire*  *Ensure that the toy elephant can move on its own*  *Make the toy elephant water resistant*  *Make balls in the feet of the toy elephant so it can roll* | *Make magnets in the legs so that the toy elephant can be hung up or is able to climb*  *Make a zipper in the toy elephant so you can put stuff in it* |
| **Low originality** | *Make sure the toy elephant can make sounds*  *Make the toy elephant so that he can bounce* | *Make the toy elephant in all kinds of different colors*  *Make the toy elephant bigger*  *Make clothes for the toy elephant, then you can dress and undress him*  *Make the toy elephant extra soft*  *Tie bandages around the toy elephant so the elephant is sick*  *Make a tablet on his belly with mini games*  *Leave out the tusks on the toy elephant*  *Make folding tusks with sharp points* |

**S3 Appendix.** Questionnaire with idea generation tasks and psychological variables

This appendix contains screenshots of each page of the first questionnaire where idea generation tasks were conducted and psychological variables (i.e., risk preference and personality traits) were measured.

.

**Intro (presented to all participants)**


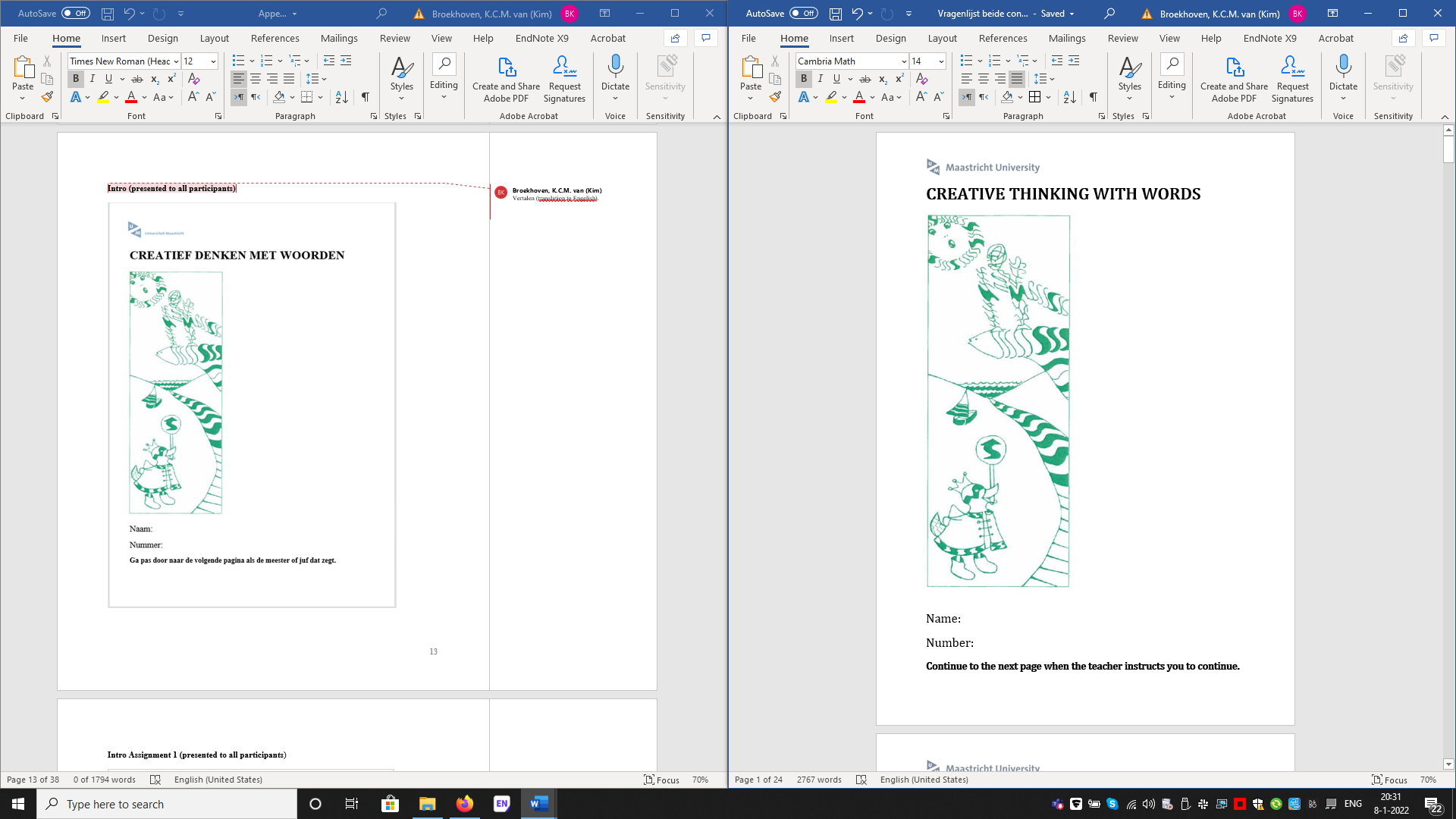


**Continuation Intro (presented to all participants**)


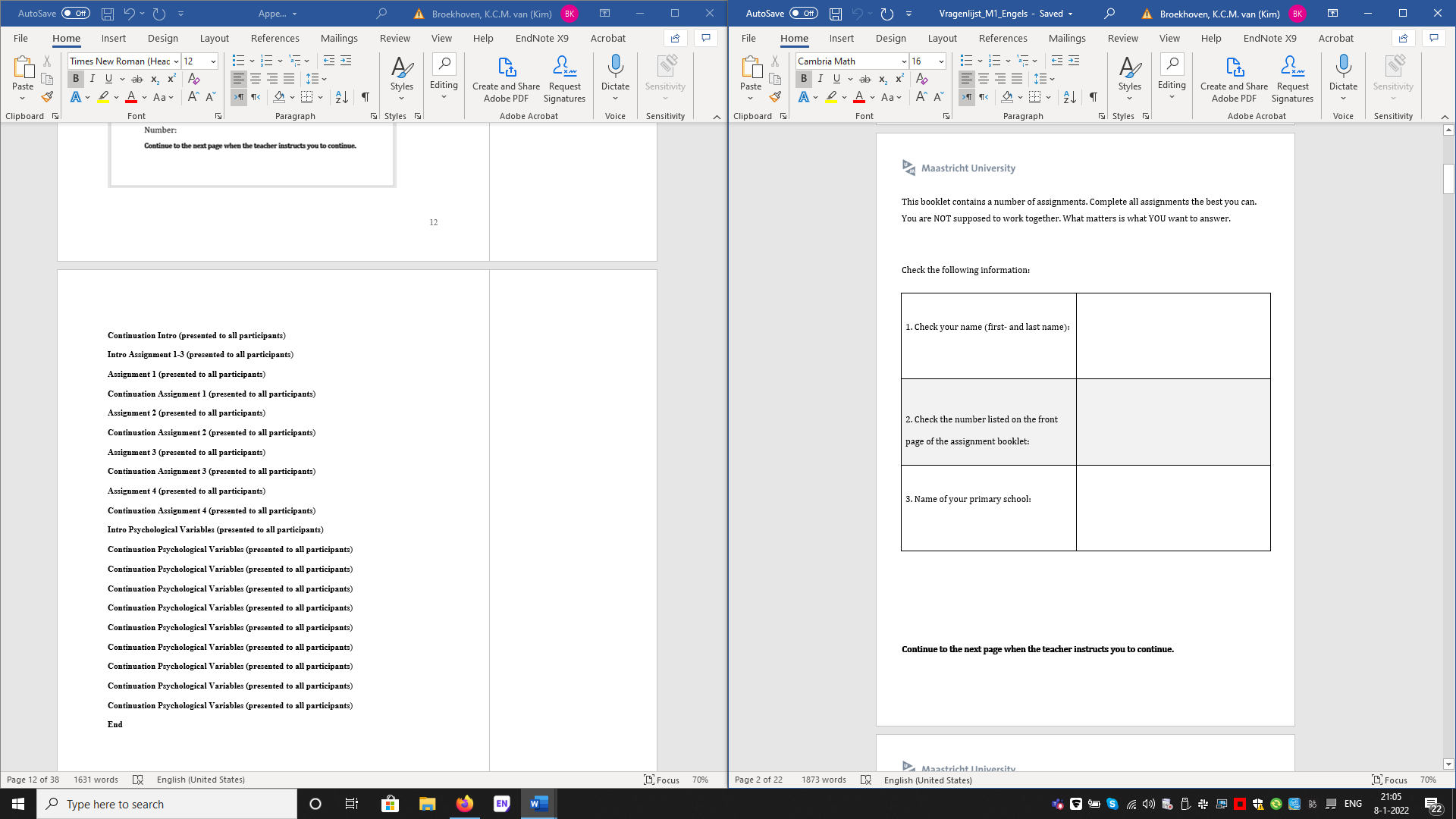


**Intro Assignment 1-3 (presented to all participants**)


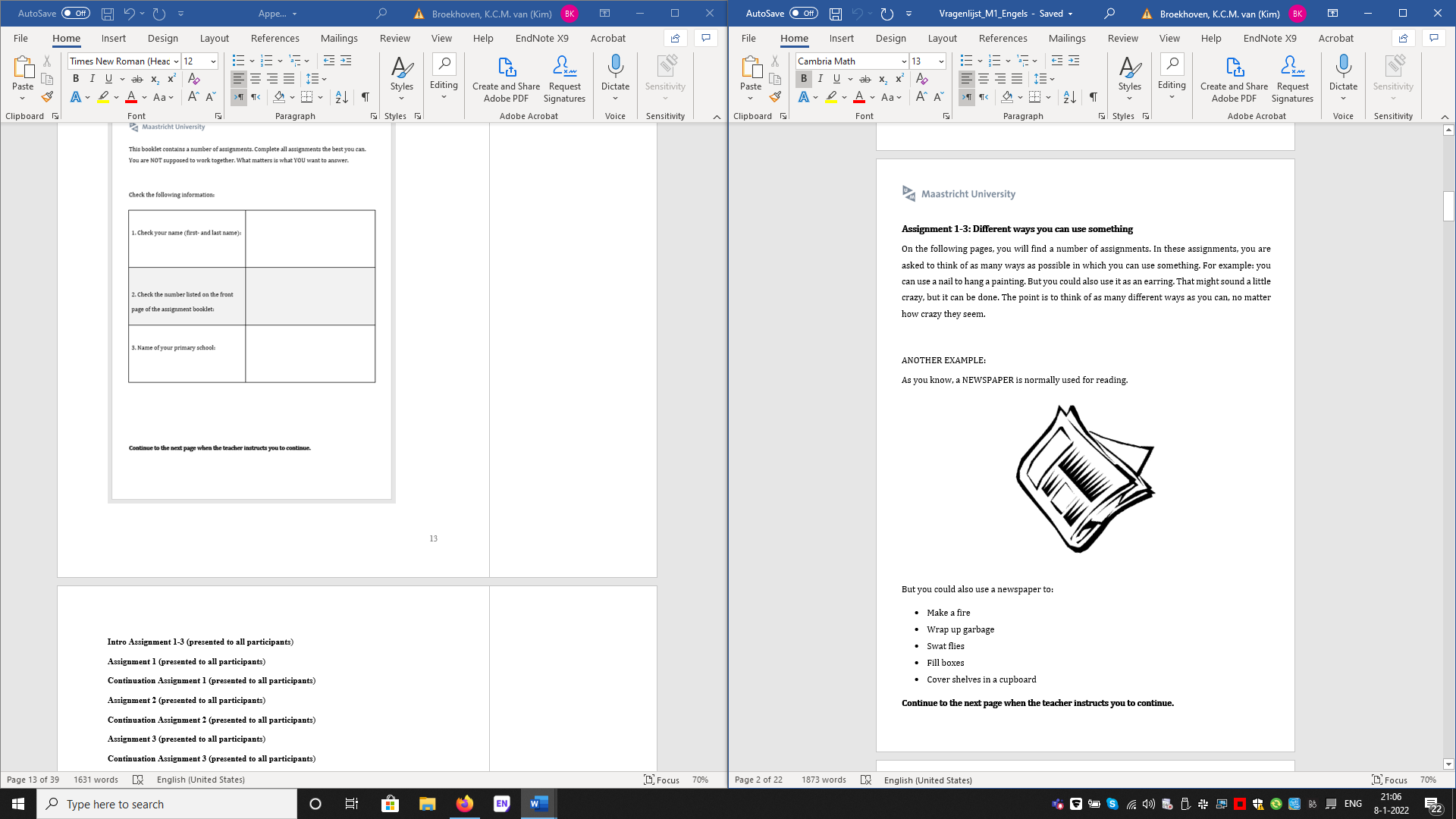


**Assignment 1 (presented to all participants**)


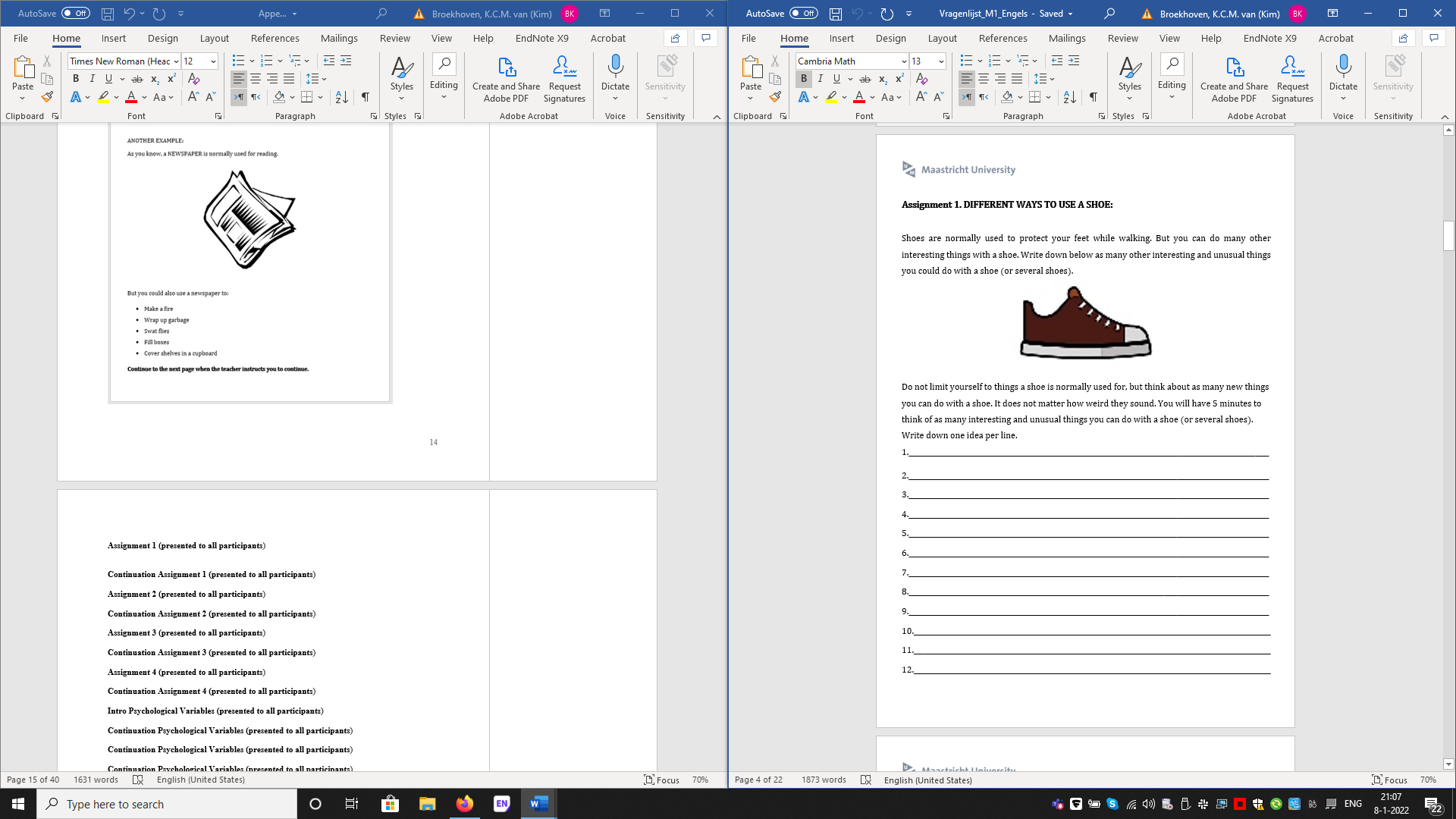


**Continuation Assignment 1 (presented to all participants**)


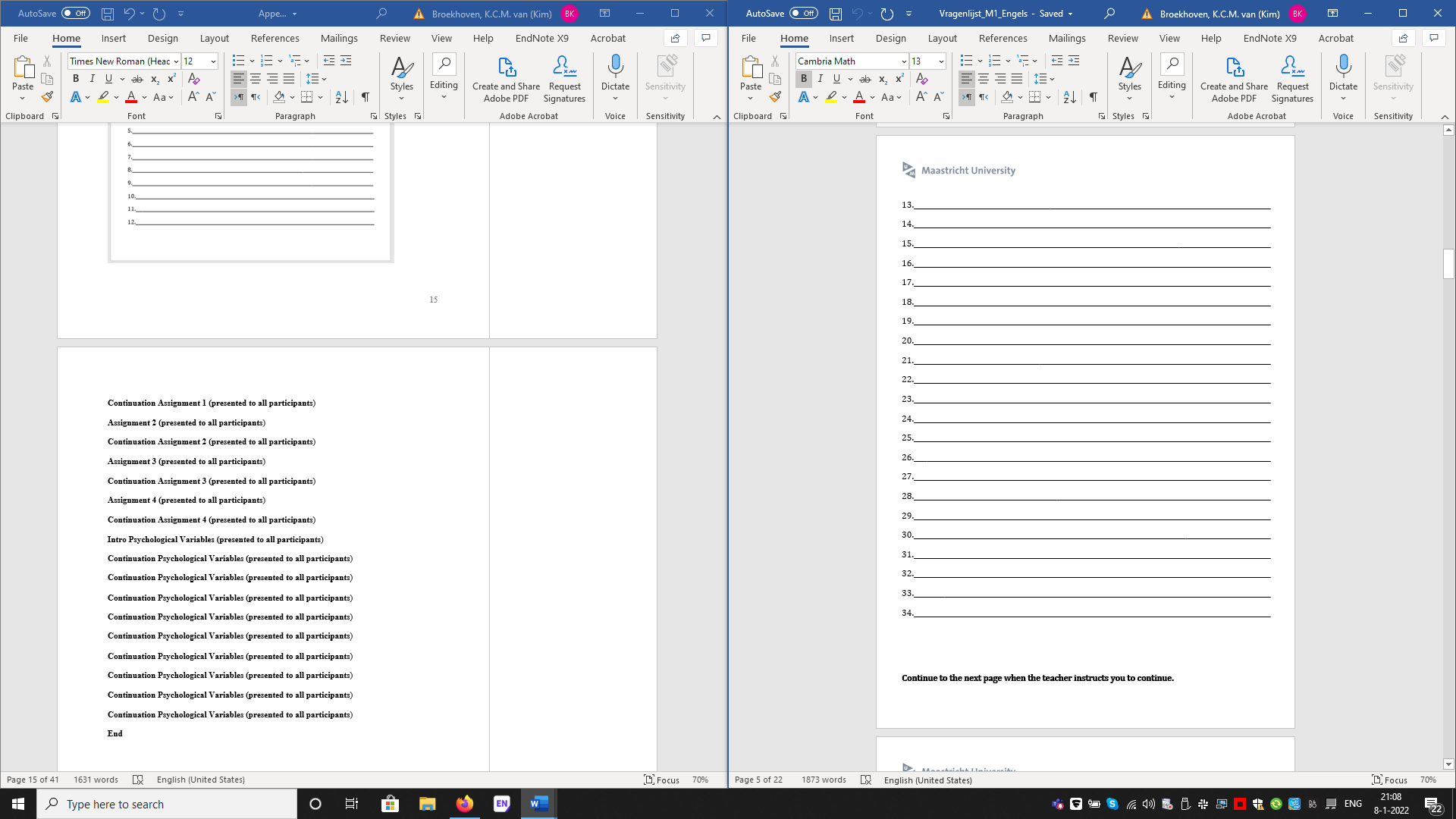


**Assignment 2 (presented to all participants**)


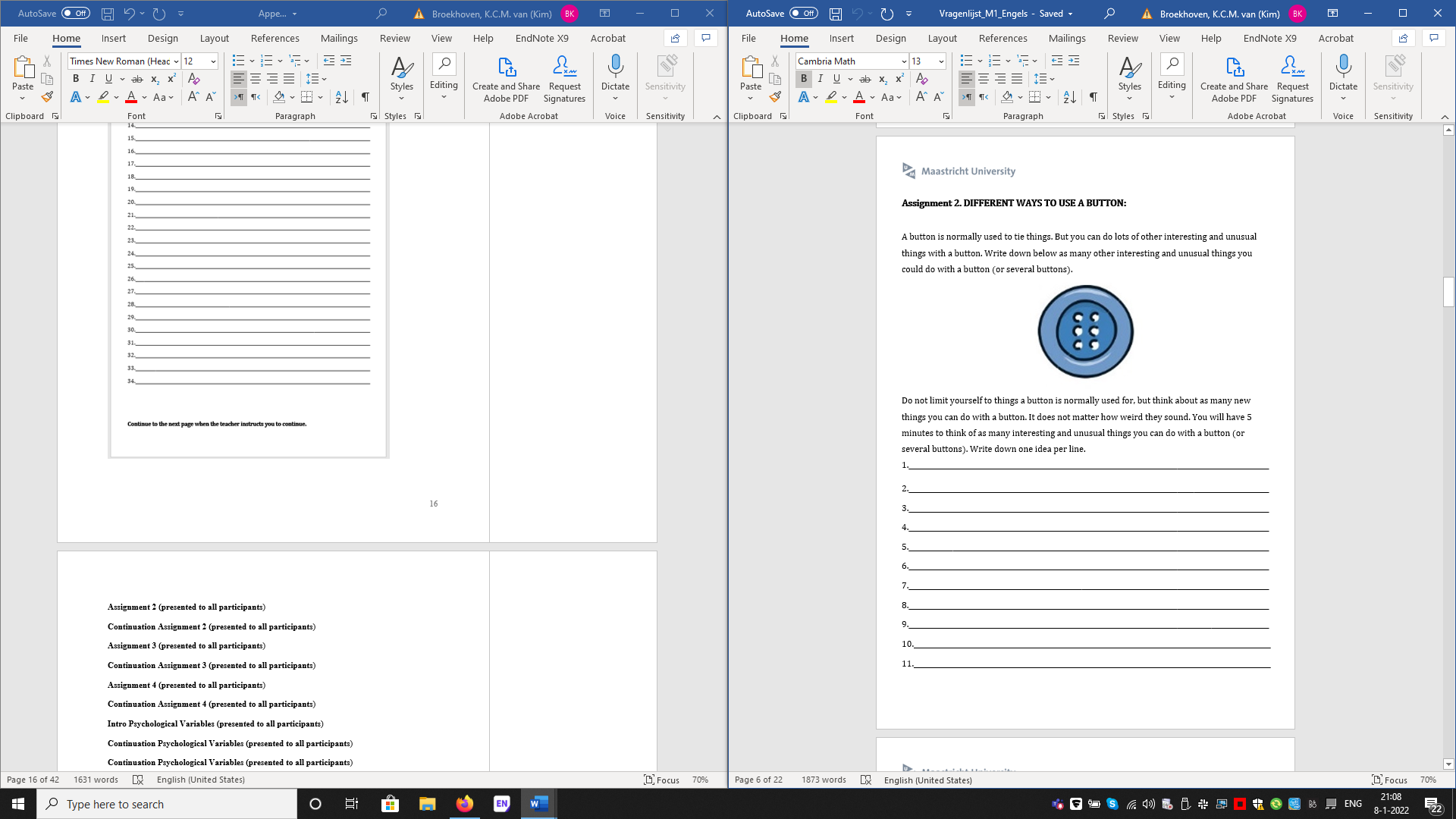


**Continuation Assignment 2 (presented to all participants**)


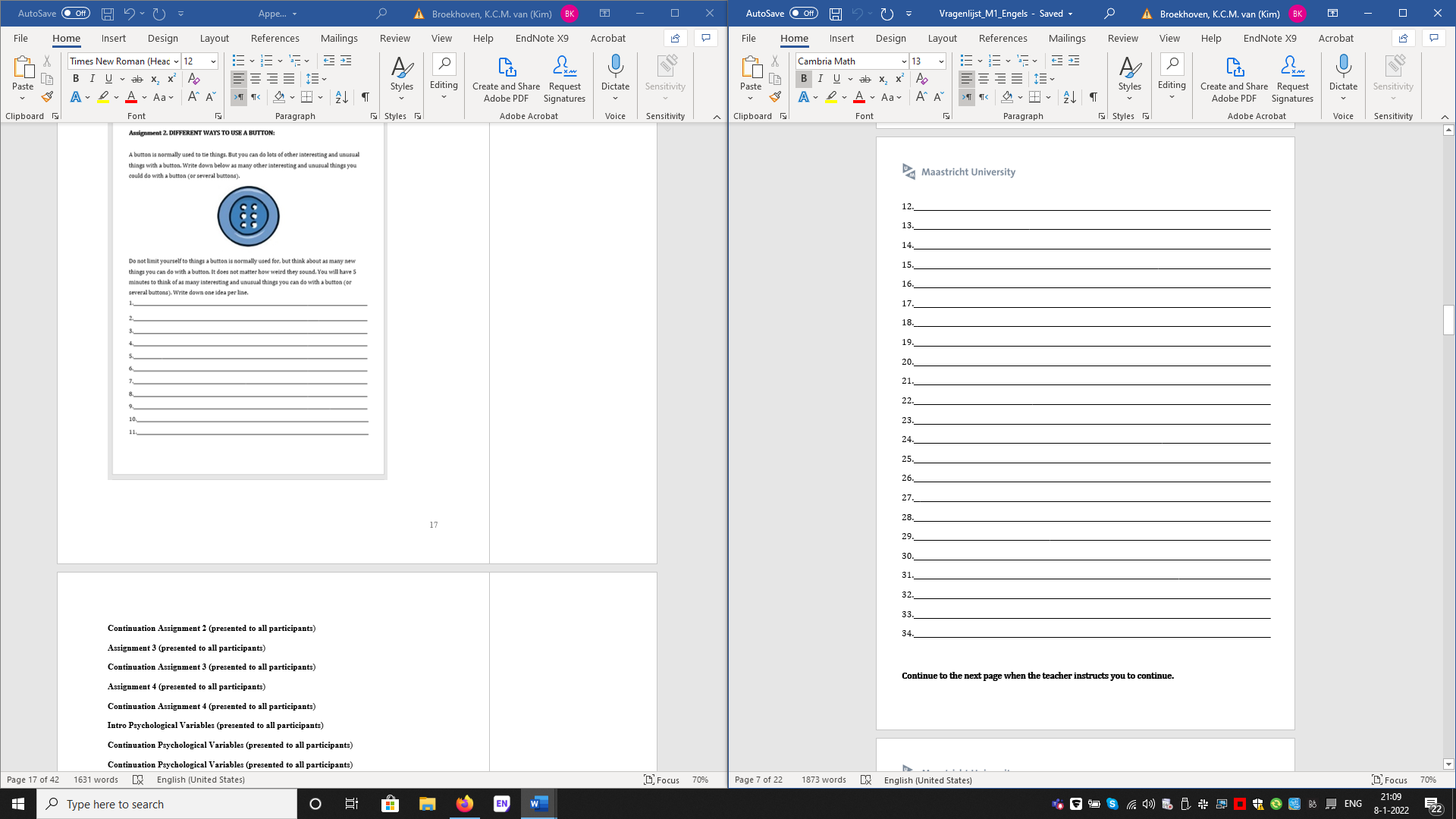


**Assignment 3 (presented to all participants**)


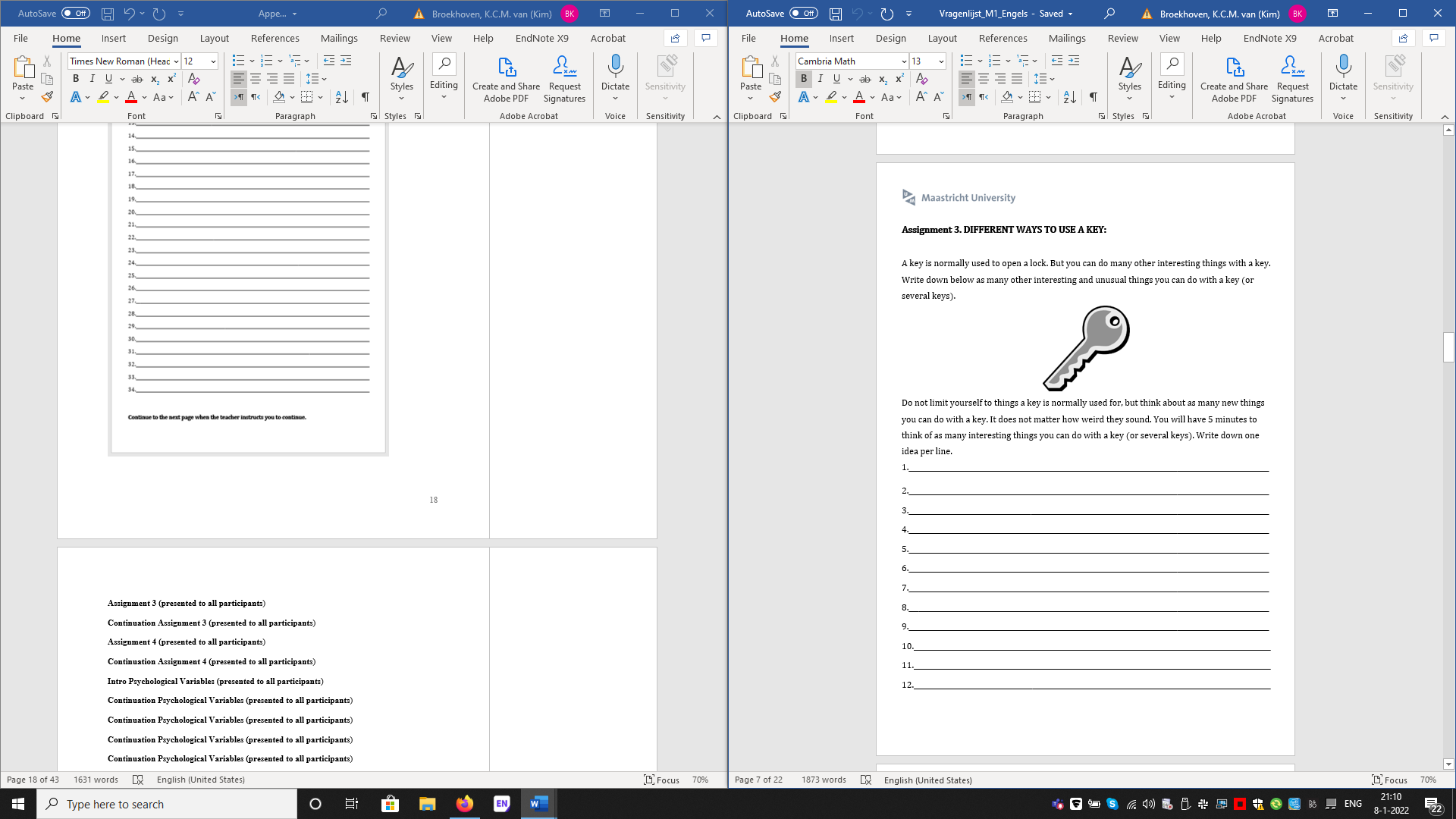


**Continuation Assignment 3 (presented to all participants**)


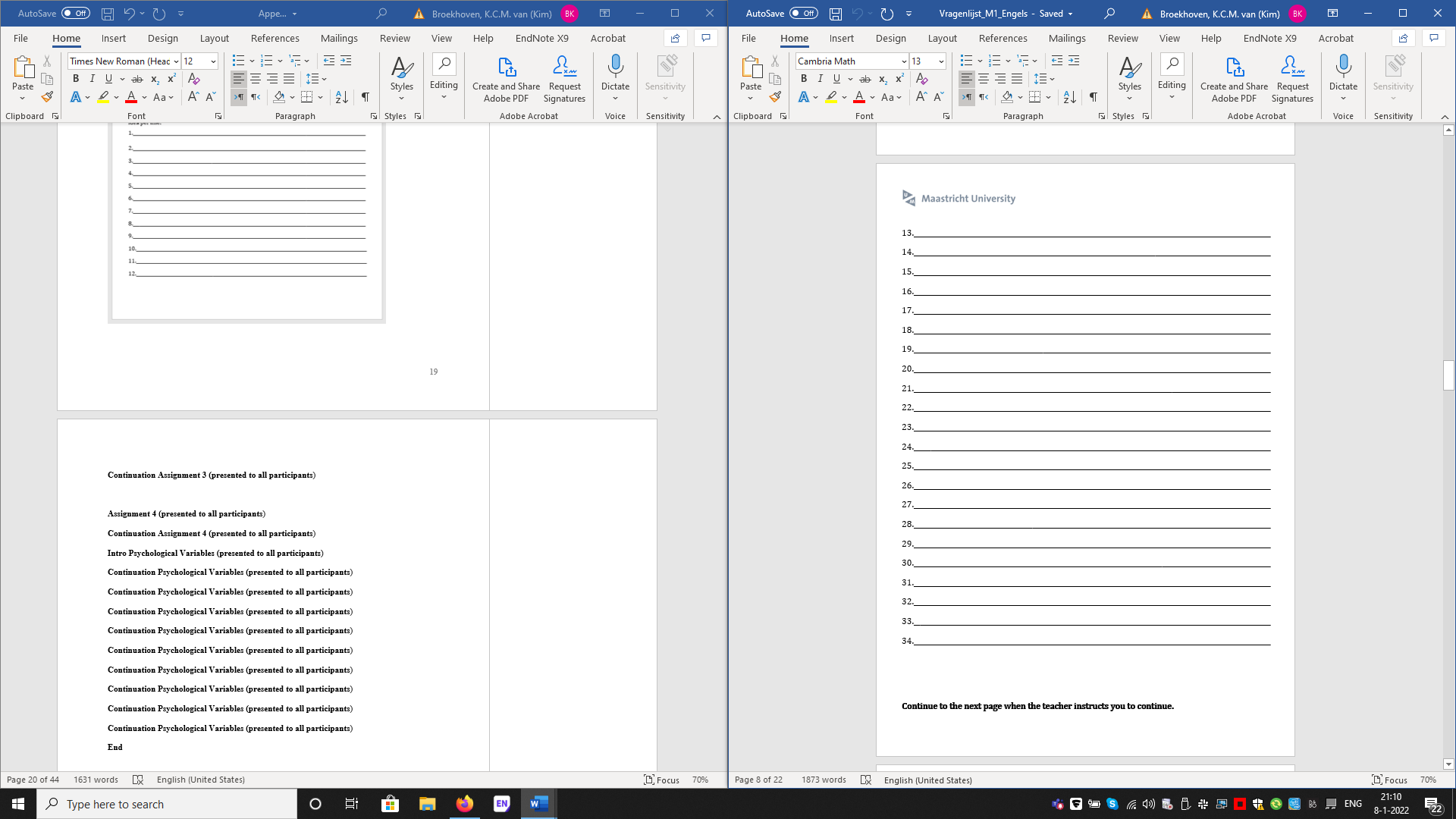


**Assignment 4 (presented to random half of participants**)


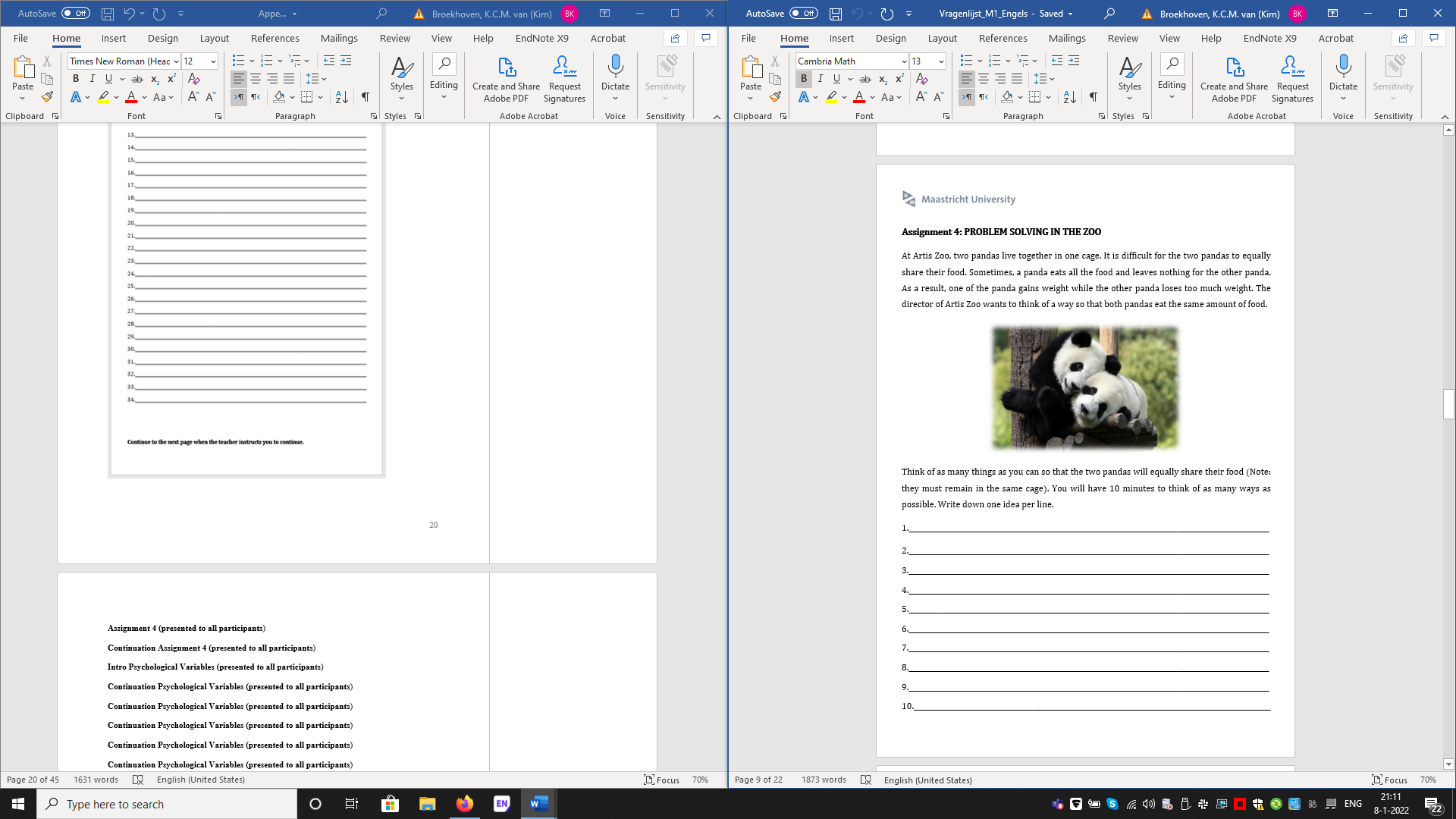


**Continuation Assignment 4 (presented to random half participants**)


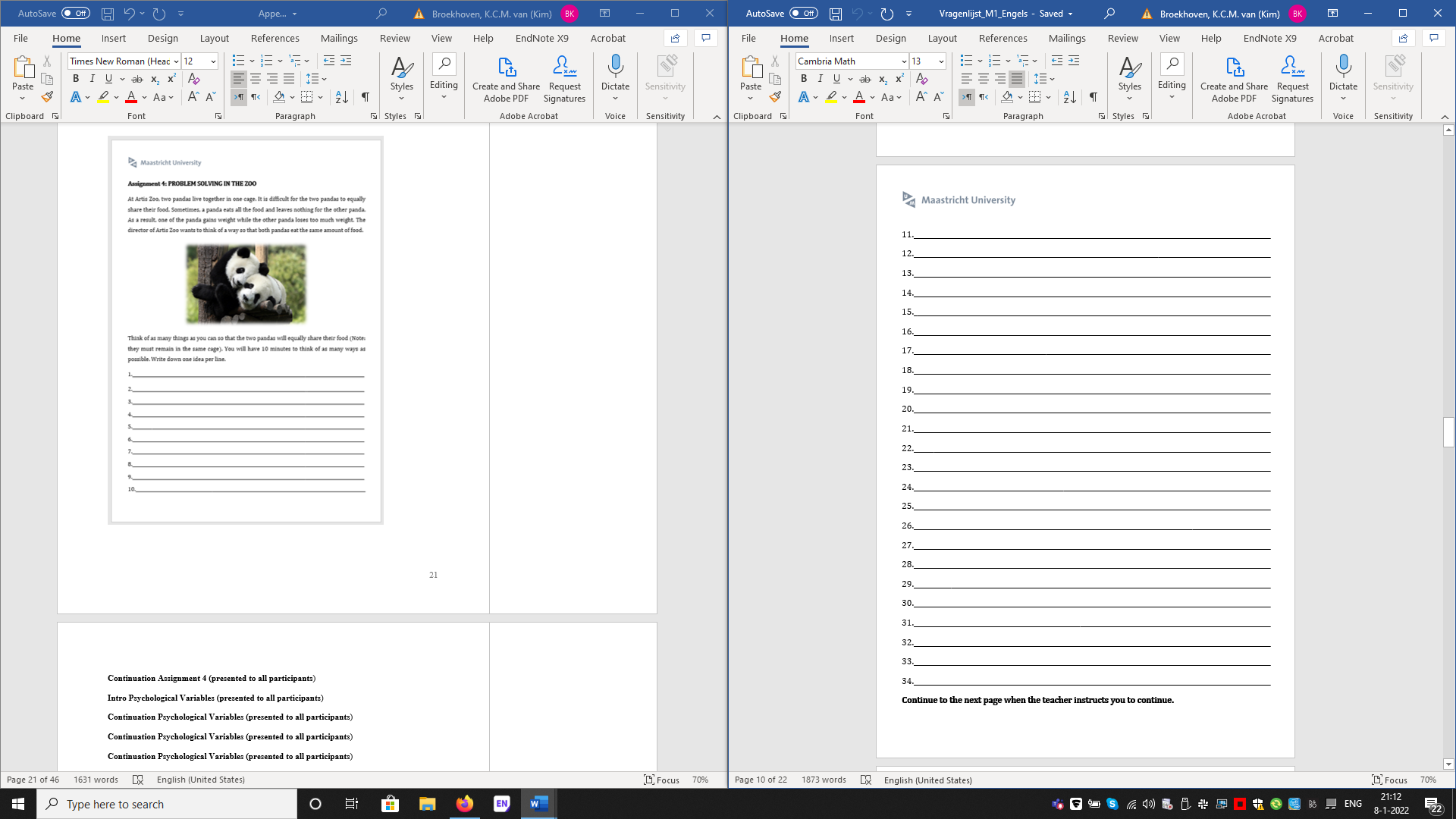


**Continuation Assignment 4 (presented to random half participants**)


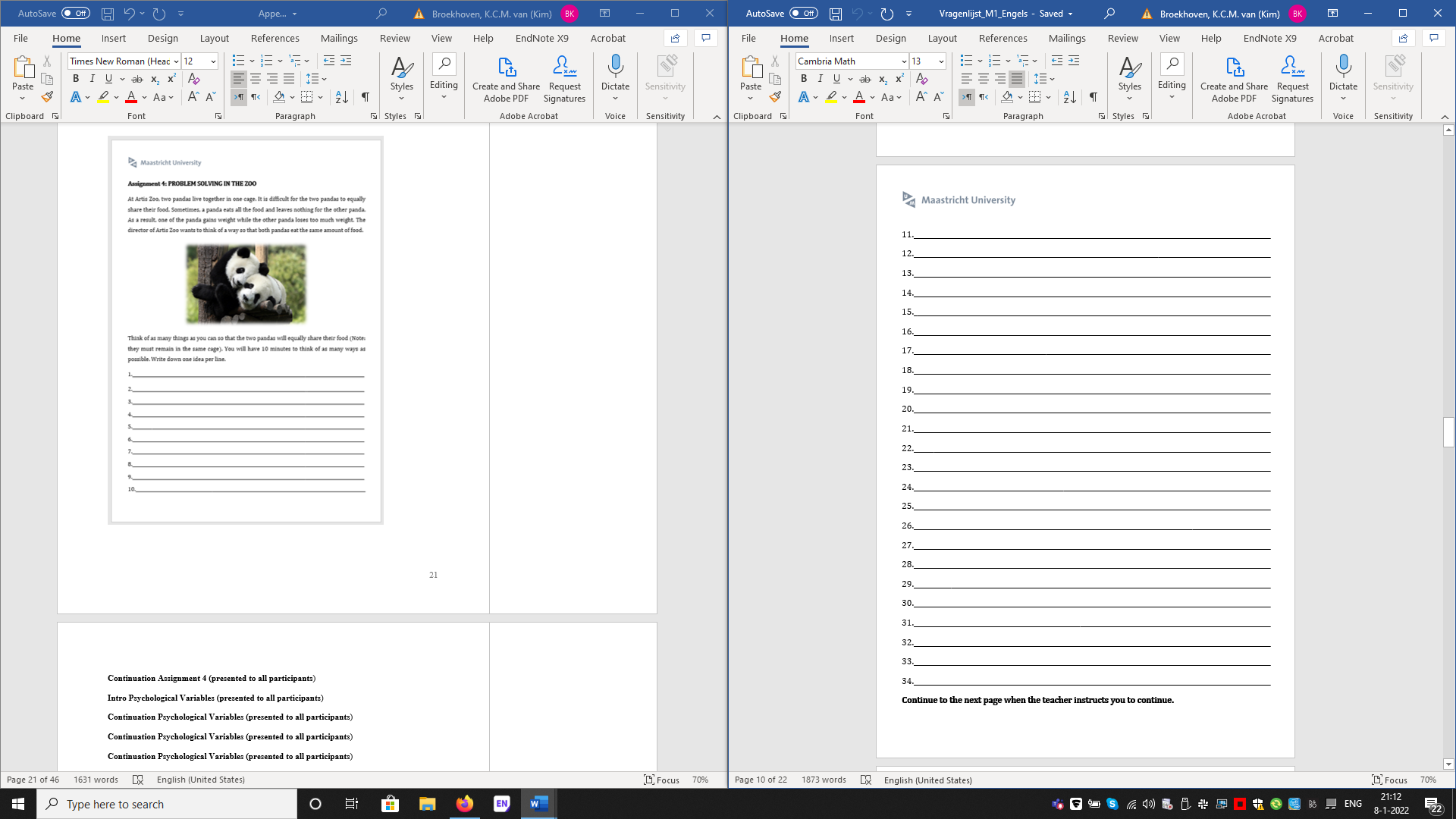


**Intro Psychological Variables (presented to all participants**)


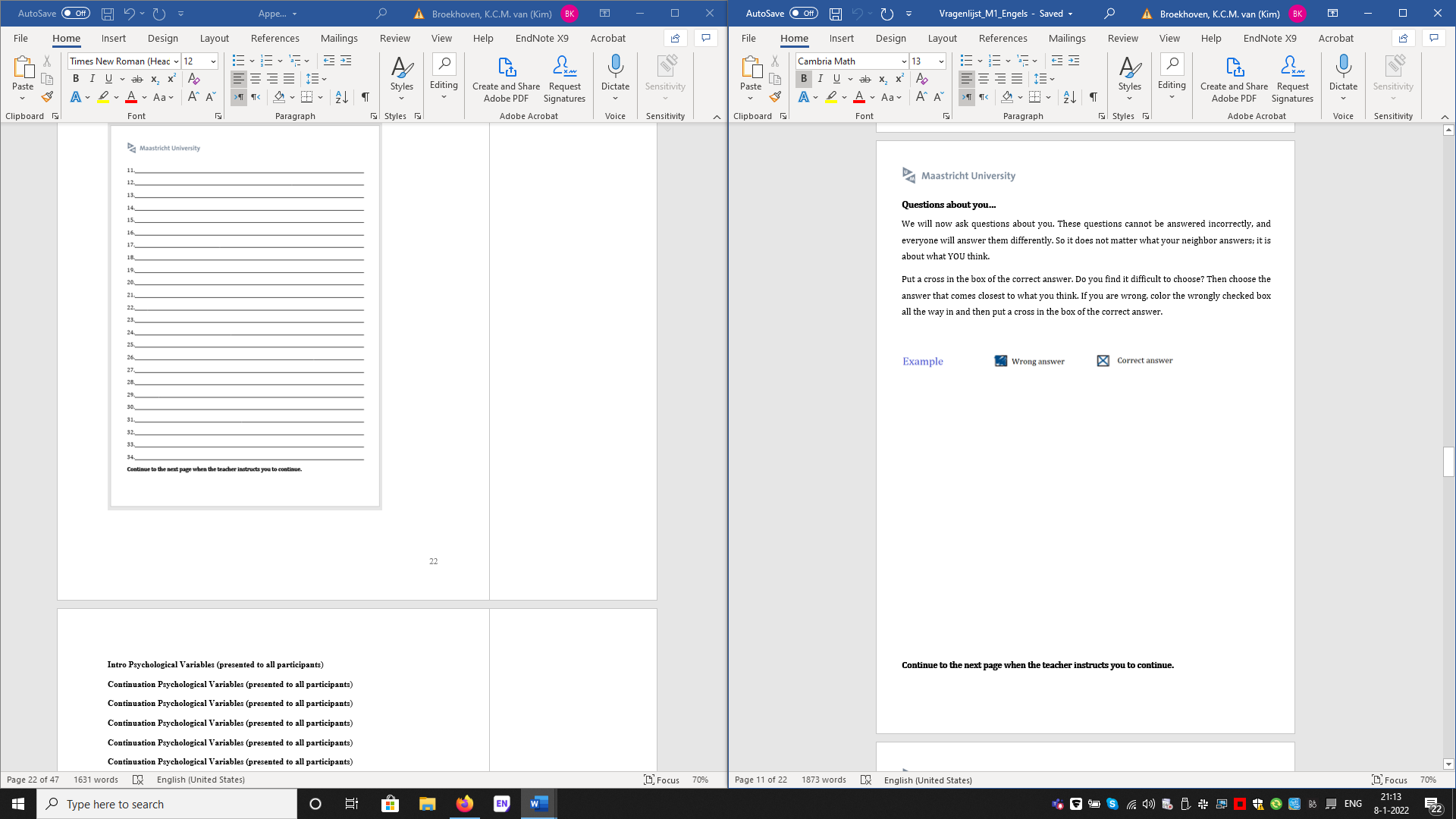


**Continuation Psychological Variables (presented to all participants**)


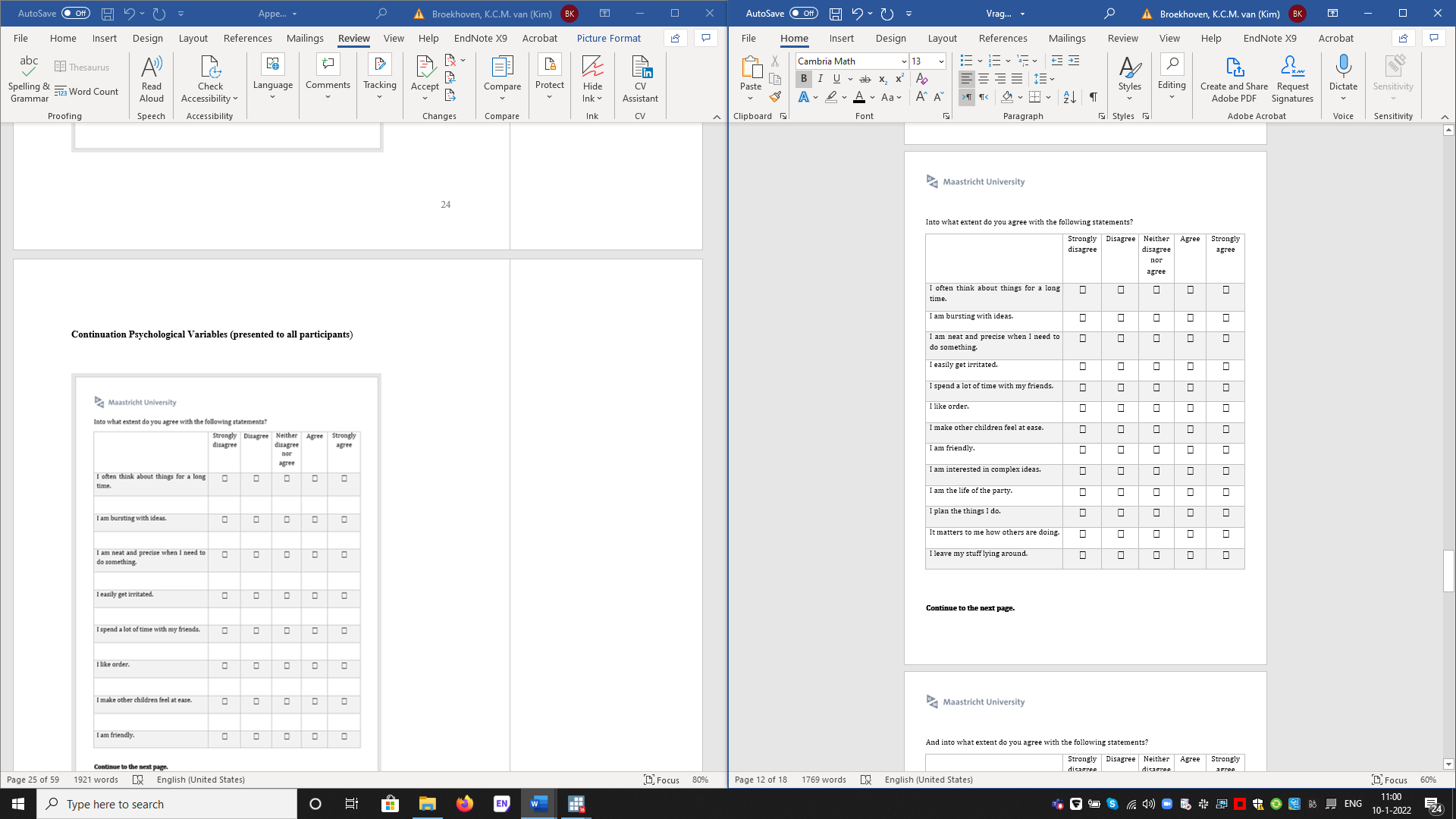


**Continuation Psychological Variables (presented to all participants**)


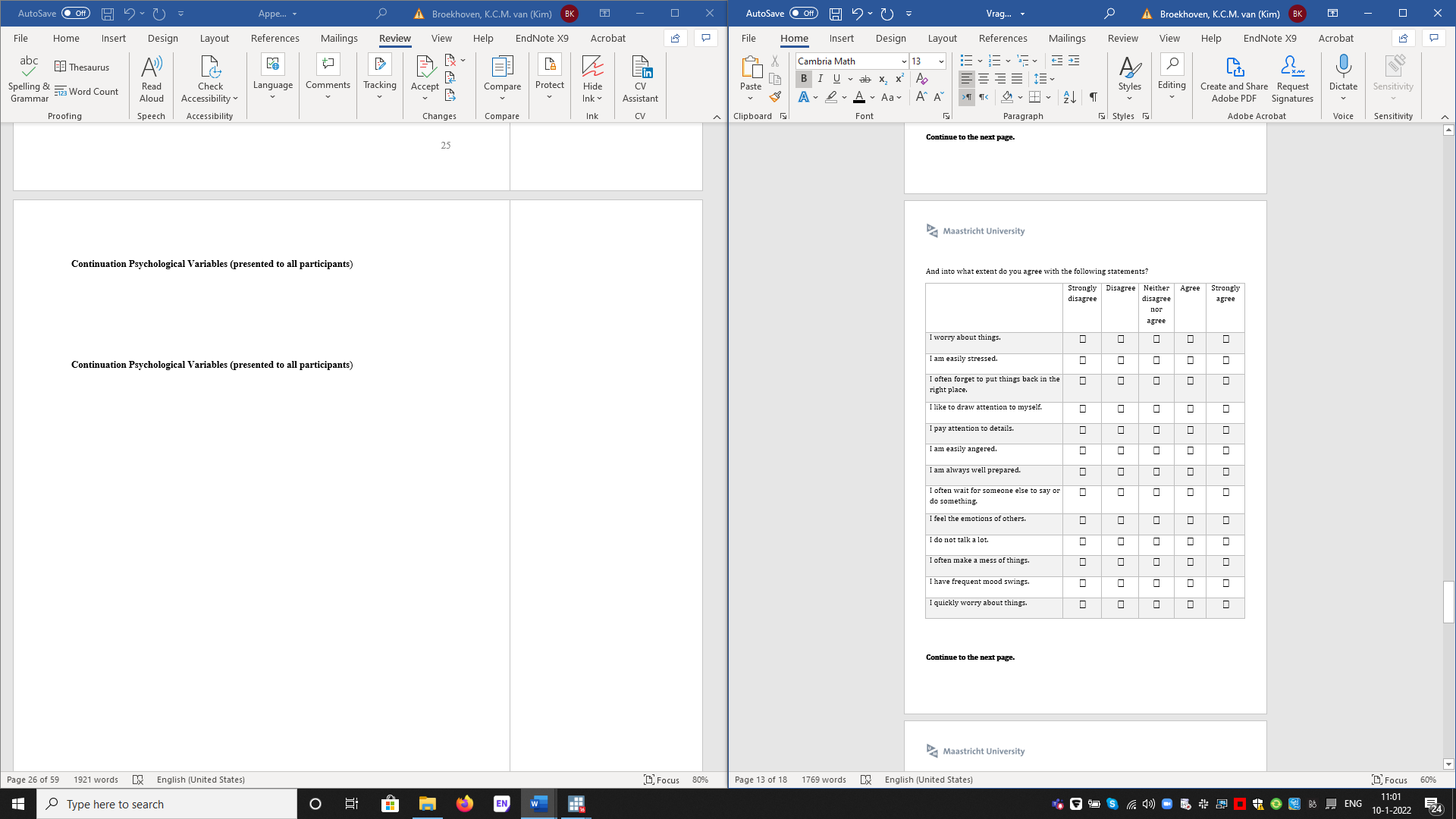


**Continuation Psychological Variables (presented to all participants**)


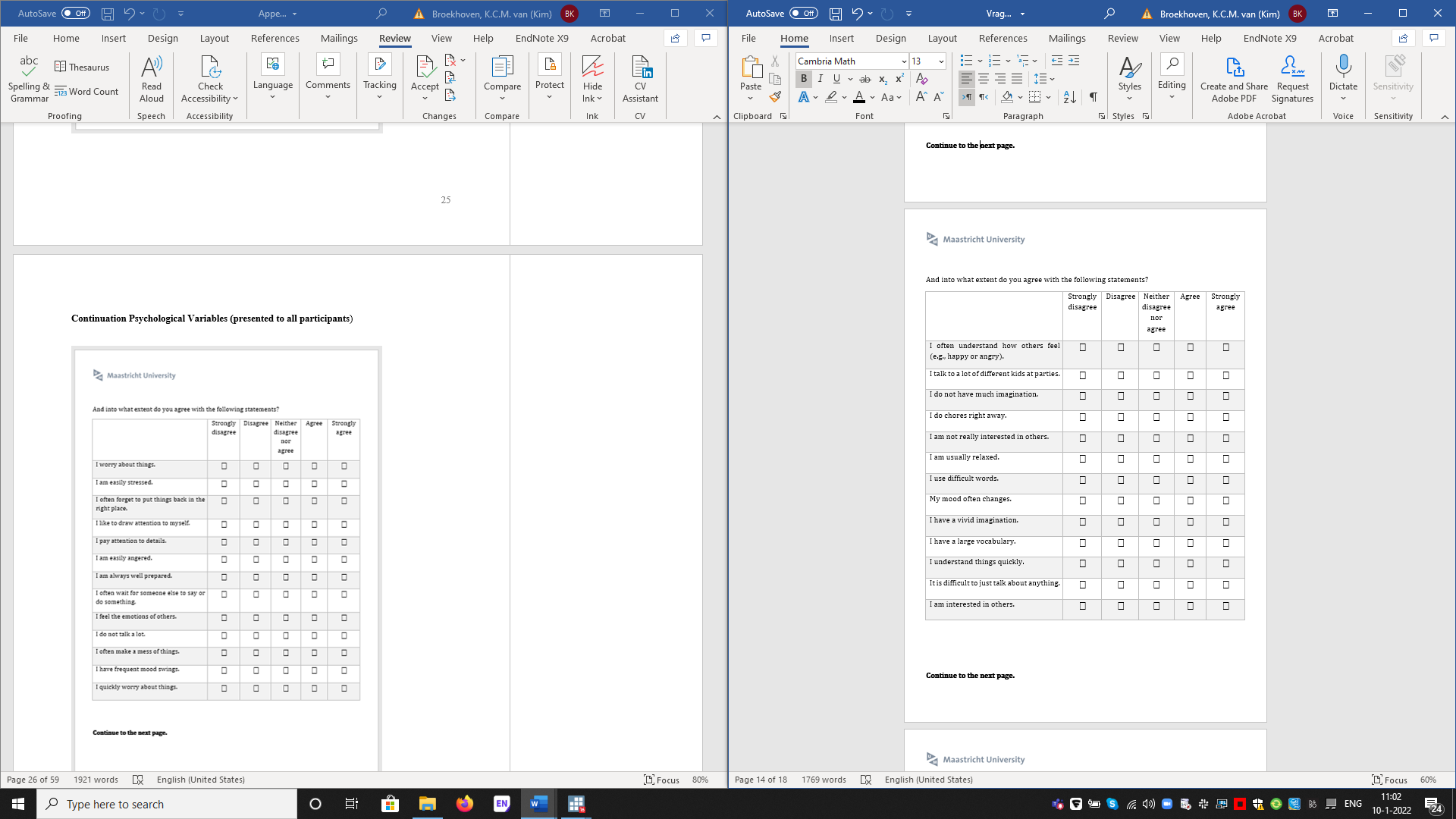


**Continuation Psychological Variables (presented to all participants**)


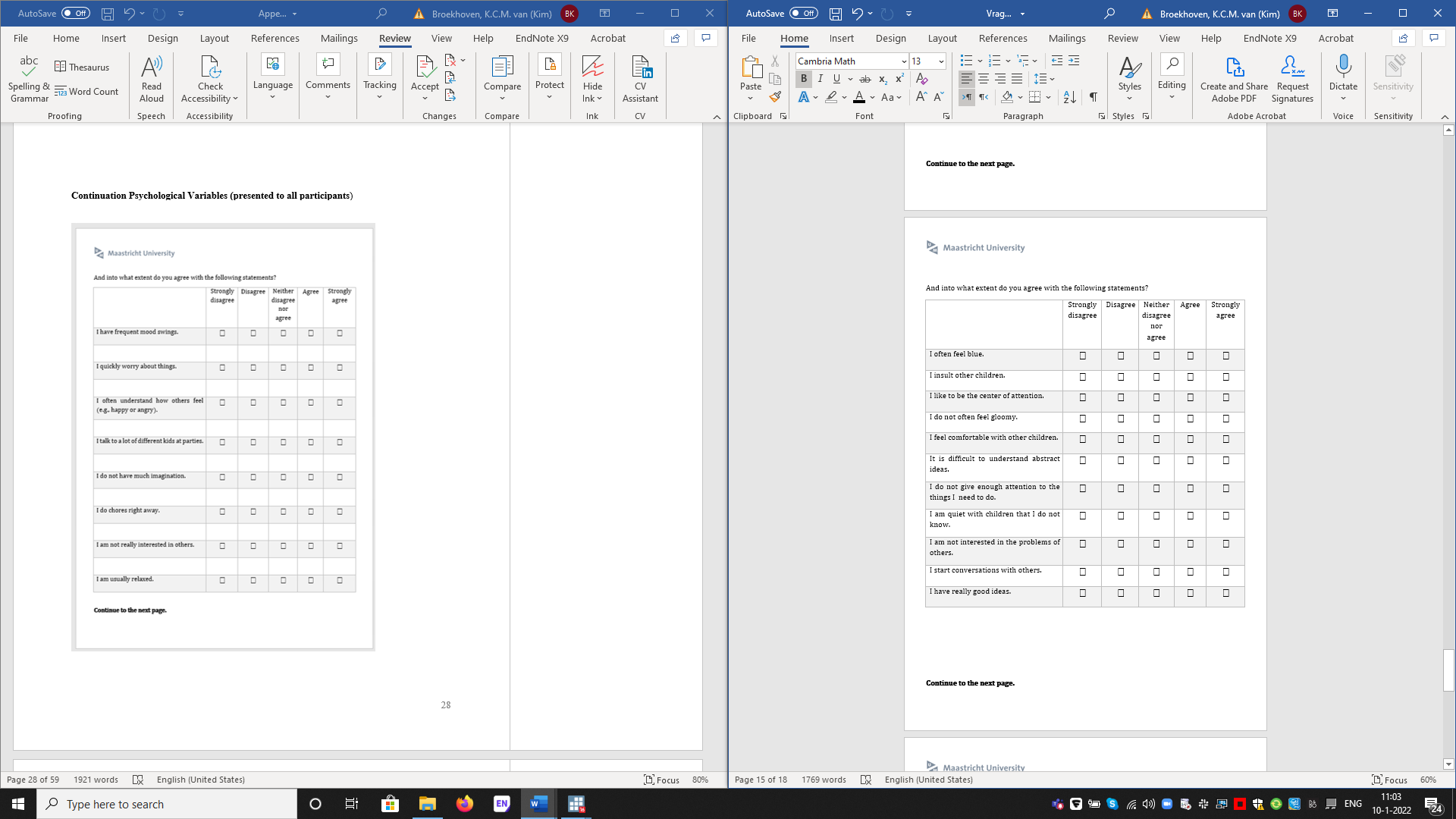


**Continuation Psychological Variables (presented to all participants**)


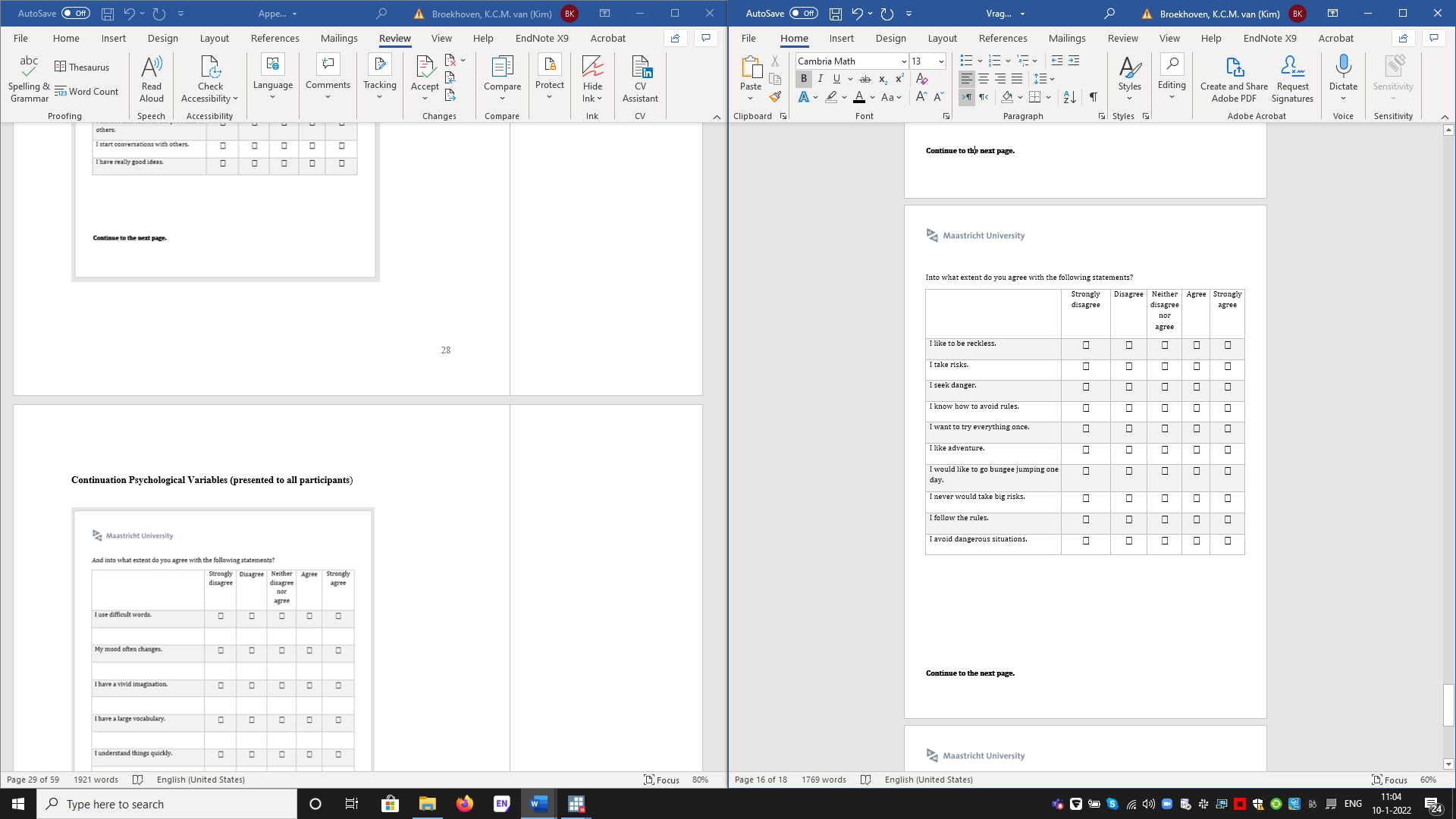


**End**


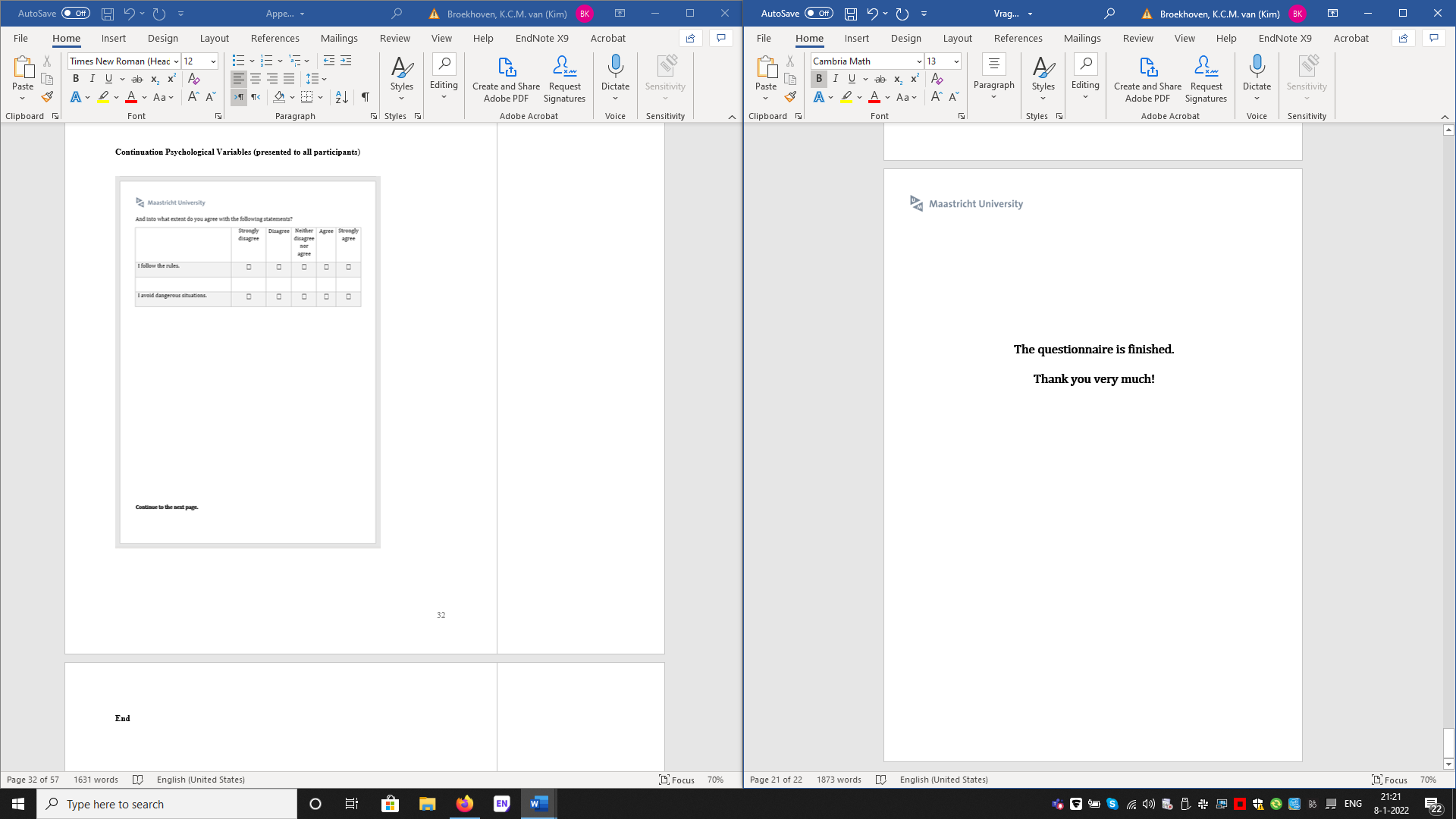


**S4 Appendix.** Examples of final products for the toy elephant and toys for monkeys in the zoo

This appendix contains photographs of the ideas that the children built, separately for the stuffed toy elephant and toys for monkeys in the zoo.


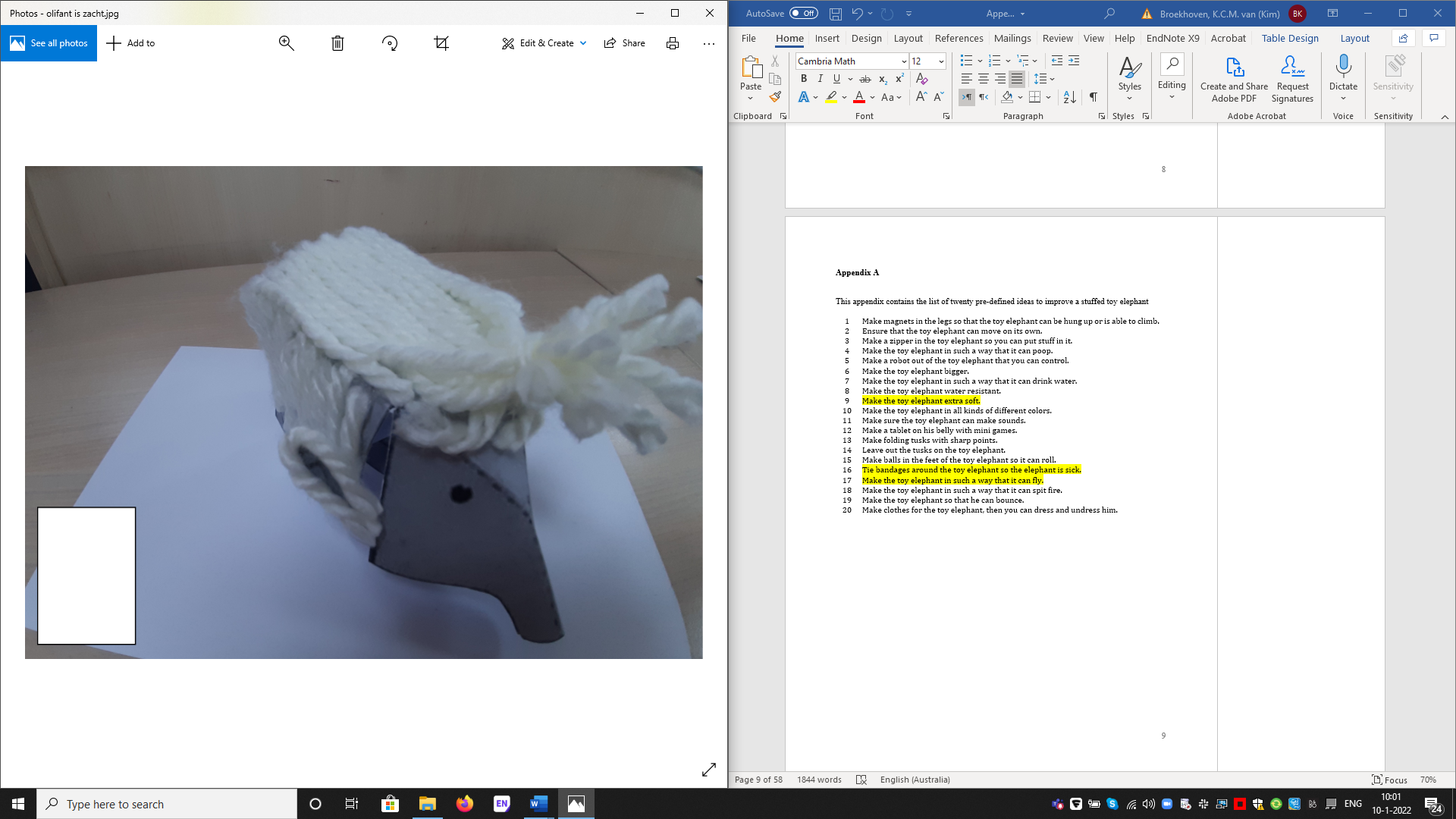

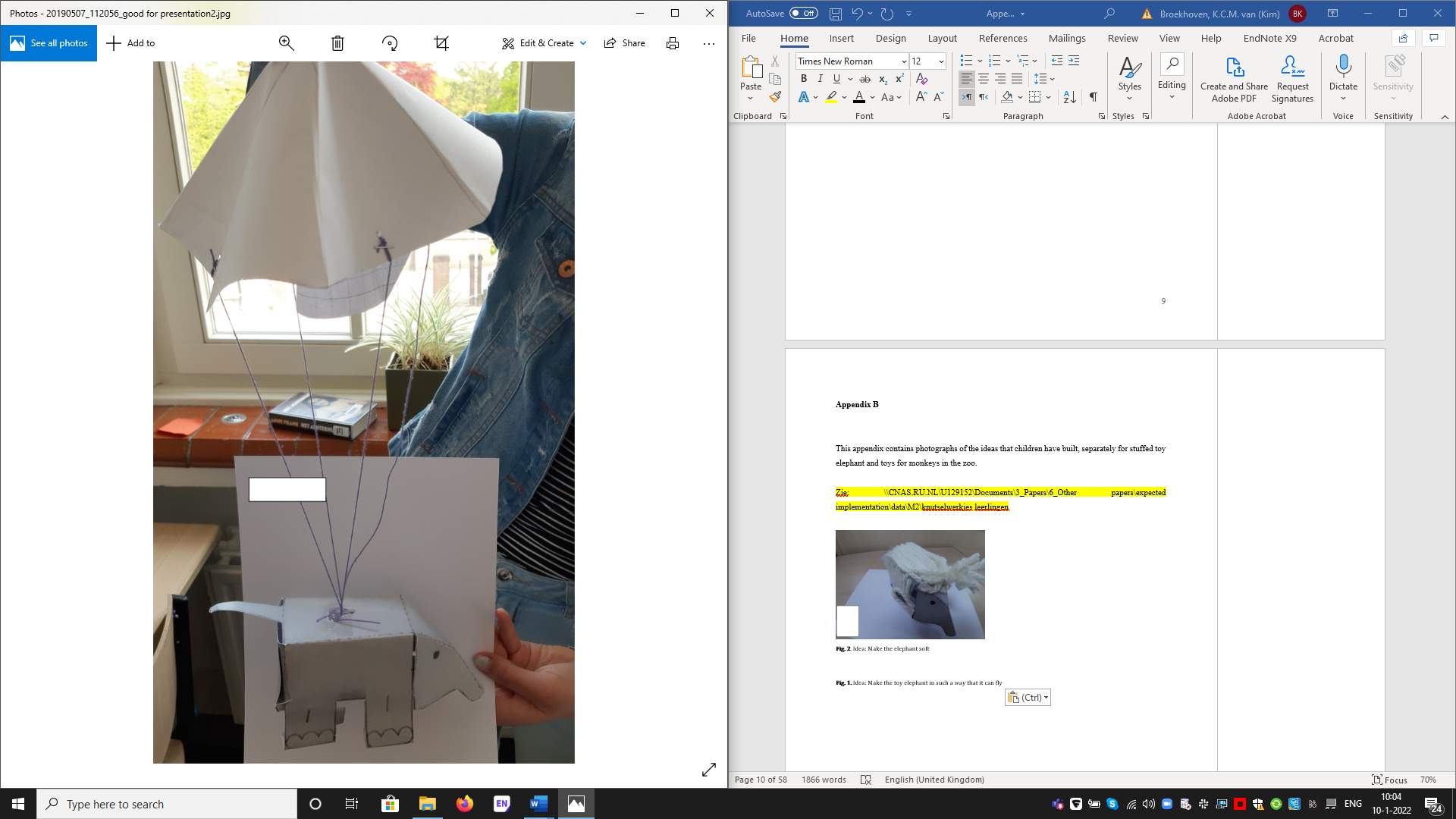


(A) (B)

**Fig. 1.** Two examples of final products for the toy elephant. Picture A presents the idea of “make the elephant soft,” and picture B presents the idea of “make the elephant in such a way that it can fly.”


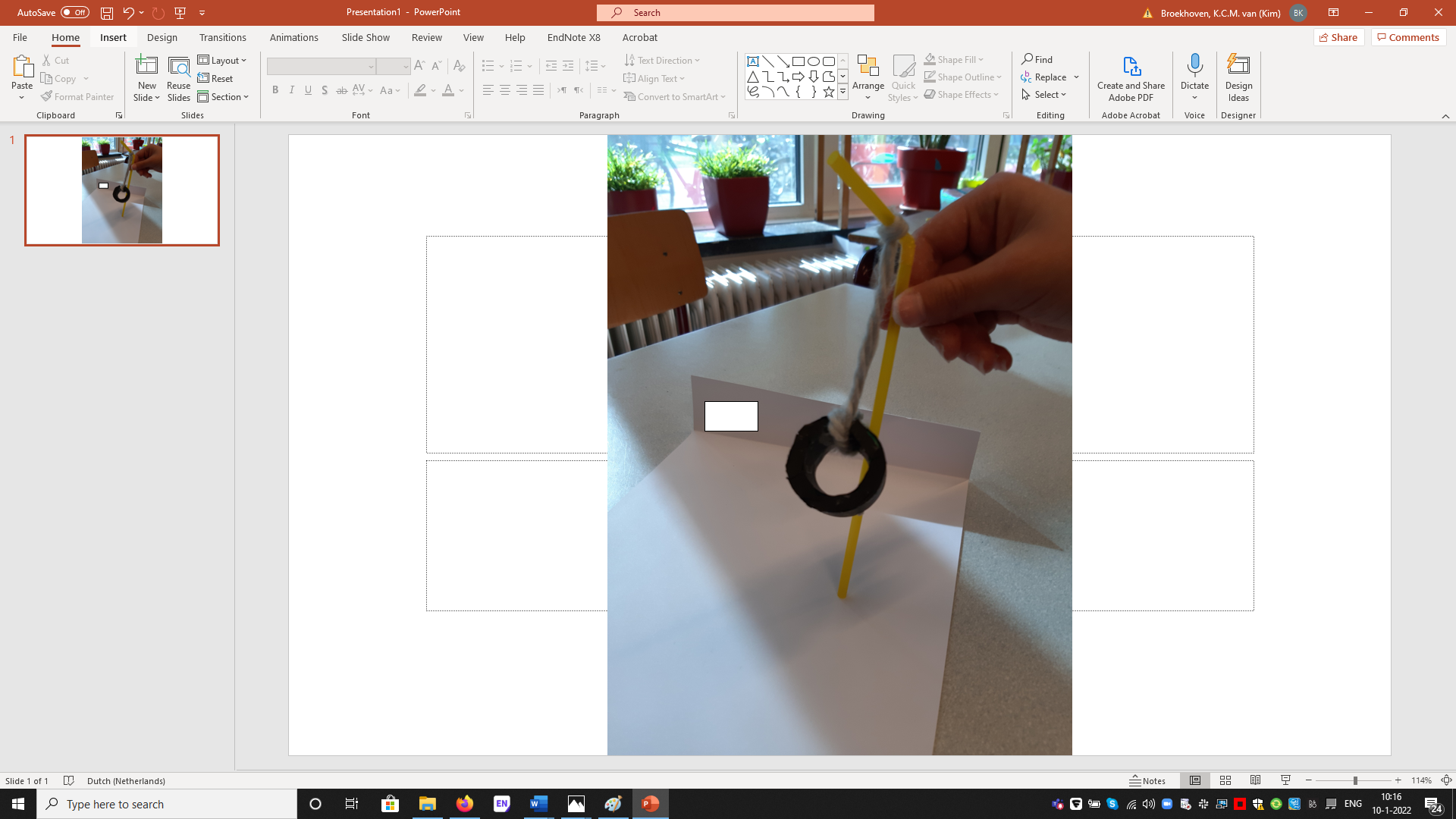

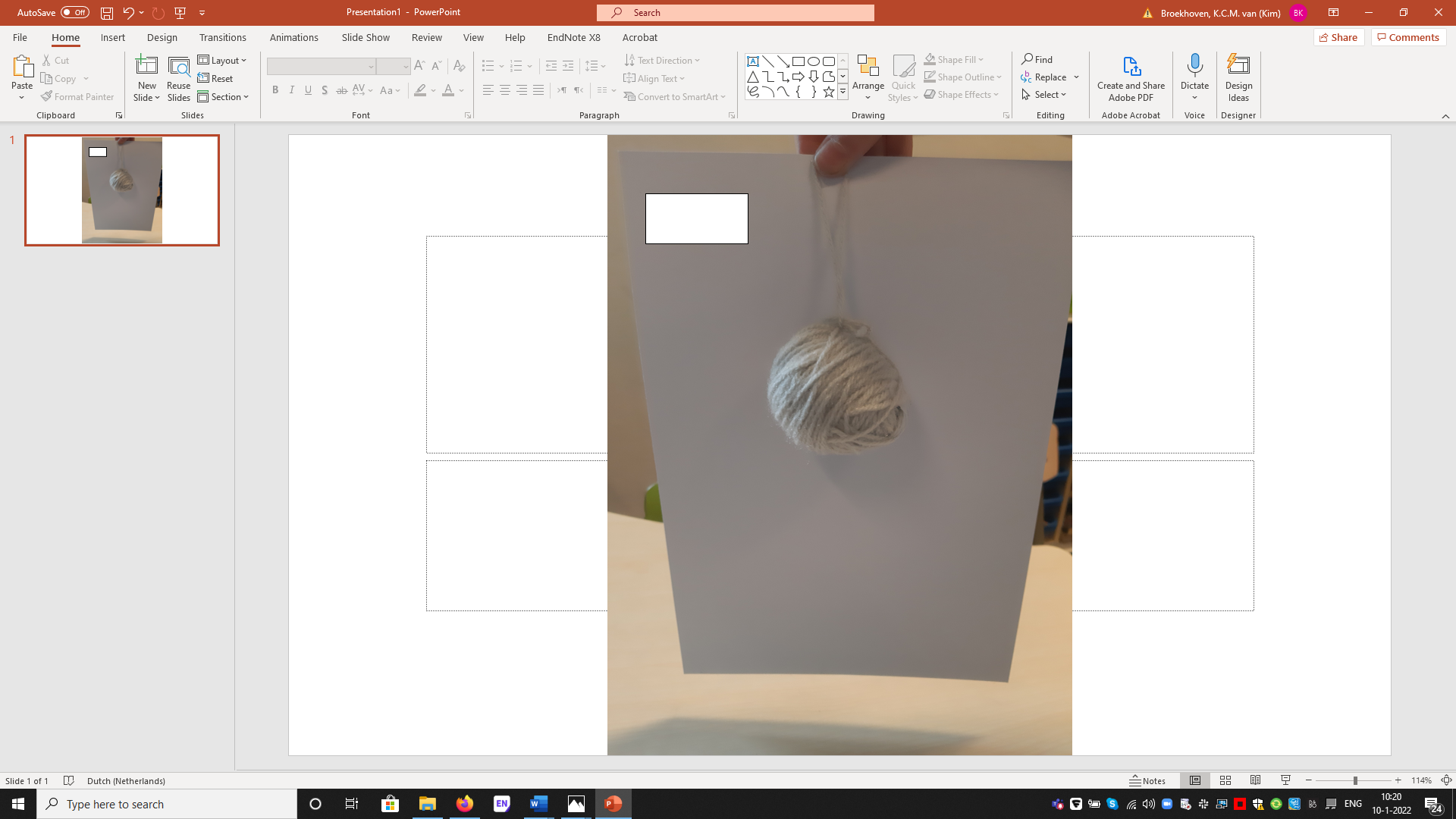


(A) (B)

**Fig. 2.** Two examples of final products for toys for monkeys in the zoo. Picture A presents the idea of “make a pendulum swing for monkeys,” and picture B presents the idea of “create a dangling ball toy for monkeys.”
