# Supplementary material for: Instructing children to construct ideas into products alters children’s creative idea selection in a randomized field experiment
Source: PLoS One. 2022 Aug 4;17(8):e0271621. doi: 10.1371/journal.pone.0271621 (PMC9352014; doi:10.1371/journal.pone.0271621)
Supplement: Attachment — Submitted filename: PLOS-24-03.pdf [file pone.0271621.s002.pdf]

Thank you for the opportunity to review the text entitled "Instructing children to construct ideas into products decreases creativity in a randomized field experiment." In the field experiment presented in this article, 403 children aged 10 to 13 were asked to select the most original ideas to make a toy more fun to play with. A random half of children was told that after the idea selection, they are expected to craft the chosen ideas—to transform them into tangible products. The other half was informed that they are going to engage in another task after idea selection (so they did not expect to construct anything based on the selection made). The analyses shown that children who anticipated implementing the chosen ideas tended to pick more feasible yet less original ideas as compared to their peers who did not expect crafting the ideas. Using conditional logit models, the Authors shown that the probability of choosing a feasible idea increased by 103% and that of choosing an original idea declined by 14% when expecting crafting the ideas. These results were consistent irrespective of children's demographic characteristics, their personality, and risk preference. Furthermore, the conclusions stayed the same with ideas' originality self-rated by children (prior to idea selection) as well as when expert judges scored the ideas for originality.

Overall, I enjoyed reading this paper very much. It is clearly written, thought-provoking, and brings important message for both creativity and education research and practice. The performed analyses are elegant and well-suited for the research questions stated. The technical aspects of the presented study are of high quality making this work a nice fit for PLOS ONE. I have mostly minor suggestions for the Authors' consideration that would make the paper even clearer and more understandable for the reader.

- The experimental manipulation definitely affected children's choices or contributed to their idea selection process. However, it does not seem accurate to say that it influenced the level of creativity of ideas. I am sure it was not the Authors' intention but several sentences suggest that due to intervention, creativity of ideas—and not children's choices—did change. That is a subtle difference but I think that the following sentences could be rephrased:
    - "(…)instructing children to construct original ideas into tangible and physical products may unintentionally decrease the creativity of the ideas children select."
    - "To investigate this, we tested whether instructing children to construct original ideas into tangible products affects the level of creativity of the ideas."
    - Also, from this perspective, the article's title seems to be imprecise and somewhat confusing.

  For comparison, I think the following expression describes what is actually posited in this study much more precisely: "(…)the prospect of implementation itself may affect idea selection, leading to the question of how the expected implementation of ideas affects children's idea selection."
- I understand what the Authors meant by "we conceptualize creativity as the level of creativity of the final selected idea (i.e., the originality and usefulness of an idea)" but it sounds somewhat tautological. I would say that in this study, creativity was understood as a set of specific characteristics of ideas that children selected, and these characteristics include originality and usefulness. To put it even more simply: creativity was conceptualized as the level of originality and usefulness of ideas.
- Considering expected idea implementation to be an example of extrinsic constraints really caught my attention. I think this is an interesting view yet it may require some explanation. One could argue that creative process is an action that results in an original and valuable product. Thus, from this perspective, idea implementation seems to be almost definitional part of creativity (or creative

process, to be more precise). Therefore, treating idea implementation as a kind of constraint is not very typical. I wonder whether the Authors could elaborate on that a bit more.

- What kind of judgments do the Authors refer to in the following sentence: "When making judgments, people often favor the more common ideas due to risk perception with creative ideas, as creative ideas are by definition uncertain, because they are often new and untested." Is it still a reference to Sorrentino's and Roney's work?
- "field settings allow a more realistic setting" → again, I know what was meant here, but I recommend rephrasing this part.
- "The children (49.9% girls, 50.1% boys)" → I think reporting the percentage for either one or another would work.
- Children's age range could be moved from *Procedure* section to *Participants* section.
- "To reduce the list of 63 ideas even further, a set of 20 ideas was selected to be presented to the children in the experiment (see Appendix C)." What were the criteria for this stage of selection?
- I think the subsection "Idea selection performance" could actually consist of two parts, namely: "Idea evaluation" and "Idea selection". Now it is blended and the reader might feel a bit confused about what was the selection all about. Actually, at present this subsection seems to describe the evaluation part of the study and not the selection. I understood the whole procedure correctly only when I looked at supplementary materials. I think making a distinction into evaluation and selection part of the study would make it clearer for the reader.
- I am probably missing something but I am not sure how the outcome variable in OLS regression computed was. I understand it was the average rating for the two selected ideas but did the Authors averaged only a given child's ratings for the two ideas selected? Or did they average the average scores from the whole sample that were given to the two selected ideas?
- I appreciate how the Authors made use of conditional logit model to draw even more precise and detailed conclusions.
- "We found that children who expected implementation selected less creative ideas—i.e., more feasible and less original (…)" → as creativity straddles both originality and usefulness (feasibility), it does not seem accurate to conclude that less creative ideas were selected. Instead, I think that the idea of originality-usefulness trade-off fits particularly well to this paper's narration, and could be emphasized throughout the article.
- "(…) degrees of feasibility and originality of their selected ideas were negatively correlated" → Would it be possible to provide specific estimates?
- "children inhibit themselves in selecting original ideas" → After all, isn't it quite an adaptive recalibration? Again, as creativity requires both originality and usefulness, maybe it is not that bad to make children be more pragmatic and realistic when it comes to choosing an idea that they want to implement? This is just an observation, but I wonder whether the Authors could share some opinion on that.
- "educators should focus not only on the end-product, but particularly on the decision-making process children go through in selecting creative ideas" → I absolutely agree!
- "We may also note that while this study investigated whether the expectation of idea implementation affects idea selection among primary school children, it did not investigate the underlying mechanisms why this happens (e.g., emotional aspects of idea selection)." → I appreciate this was acknowledged and mentioned. I would love to see this important line of work develop further in such a direction.
