# Supplementary material for: Instructing children to construct ideas into products alters children’s creative idea selection in a randomized field experiment
Source: PLoS One. 2022 Aug 4;17(8):e0271621. doi: 10.1371/journal.pone.0271621 (PMC9352014; doi:10.1371/journal.pone.0271621)
Supplement: Attachment — Submitted filename: Response to Reviewers.docx [file pone.0271621.s003.docx]

**Our responses to the reviewers’ feedback**

We would like to thank the reviewers for their valuable comments that helped us to strengthen our paper. We are happy to read that the reviewers consider the manuscript well-written (reviewer 1 and 2), well-suited and well-performed statistical analyses (reviewer 1), a good integration of a controlled experimental approach in an ecologically valid school setting (reviewer 2), and a highly relevant topic that brings important messages for both creativity and education research and practice (reviewer 1 and 2). Moreover, we are extremely delighted to read that it was a joy reading this paper, and that both reviewers recommend publication with minor revisions. Below, we have addressed their points for feedback per paper section, and our responses to this.

**General remarks**

**Reviewer 1 had the following general remarks:**

a. The experimental manipulation definitely affected children’s choices or contributed to their idea selection process. However, it does not seem accurate to say that it influenced the level of creativity of ideas. I am sure it was not the Authors’ intention but several sentences suggest that due to intervention, creativity of ideas—and not children’s choices—did change. That is a subtle difference but I think that the following sentences could be rephrased:
   a. “(…)instructing children to construct original ideas into tangible and physical products may unintentionally decrease the creativity of the ideas children select.”
   b. “To investigate this, we tested whether instructing children to construct original ideas into tangible products affects the level of creativity of the ideas.”
   c. Also, from this perspective, the article’s title seems to be imprecise and somewhat confusing.

For comparison, I think the following expression describes what is actually posited in this study much more precisely: “(…)the prospect of implementation itself may affect idea selection, leading to the question of how the expected implementation of ideas affects children’s idea selection.”

Response: We have rephrased the sentences (see title and page 1, 2, and 16):

*a. ‘(…)instructing children to construct original ideas into tangible and physical products may unintentionally change children’s choices for creative ideas.’*

*b. ‘To investigate this, we tested whether instructing children to construct original ideas into tangible products alters children’s creative idea selection.’*

*c. ‘Instructing children to construct ideas into products alters children’s creative idea selection in a randomized field experiment.’*

**Reviewer 2 had the following general remarks:**

b. There has been a lot of research on how implementation impacts ideation, and the outcomes of creative processes. Some key findings from this research should be integrated in the paper. Most notably, the kind of implementation that has been required from children in this experiment is a particular kind of implementation, which indeed would be expected to foster the opposite of creatively diverse and daring ideation. This form of implementation can be characterised as follows: (i) the children’s prototypes have a relatively high resolution, (ii) they require manual building skills, (iii) there is only one prototype construction round by the end of the process, (iv) there is no explicit use of multiple prototypes to try out diverse ideas including at least one “wild” approach. In all four regards, more creatively diverse and daring solutions would have been expected if the implementation process had been different.

   (i) A well-replicated finding in creativity and innovation research is that of Edelman & Currano (2011) in their Media-Models Framework: Prototypes with a high resolution/refinement/granularity (e.g. CAD drawings, or refined paper models) foster convergent thinking, and little changes are made to already existing concepts. By contrast, rough prototypes (e.g., rough sketches or improvised, non-detailed 3D depictions) foster divergent thinking and invite deviance from existing concepts. The two sample elephants depicted in the present experiment show a notably high amount of refinement.
   Ref:
   *Edelman, J., & Currano, R. (2011). Re-representation: Affordances of shared models in team-based design. In Design thinking (pp. 61-79). Springer, Berlin, Heidelberg.

   (ii) There is a huge range of methods to implement creative ideas, and research on the impact of different implementation strategies. Building tangible prototypes is known to be delicate, especially when this requires physical building skills and thereby draws attention away from the ideas at stake. Alternative approaches include storytelling, role-playing, drawing sketches, wizard-of-oz-prototyping, building critical-function-prototypes or designing news-of-the-future. Whenever a skill-intensive form of implementation is required from people, thorough training in the specific implementation method is recommended beforehand, because only with the necessary skill for expressing ideas in a particular medium people can be free to express any kind of an idea freely. The two sample elephants from this study required a notable amount of paperwork skill to be built like this.
   Refs:
   *McKim, R. H. (1972). Experiences in visual thinking. Wadsworth Publishing, Belmont.
   *d.school (2010) Bootcamp bootleg. [https://hpi.de/fileadmin/user_upload/fachgebiete/d-school/documents/01_GDTW-Files/bootcampbootleg2010.pdf](https://urldefense.com/v3/__https:/hpi.de/fileadmin/user_upload/fachgebiete/d-school/documents/01_GDTW-Files/bootcampbootleg2010.pdf__;!!HJOPV4FYYWzcc1jazlU!8lko8B4c0dtEVikSSBTj3__lLSV9MobhAHWhwmLlOhD_kd-OIEBOBBxrsOFzUvHp5rVSw8ynvUqYLkiGW6mKjQ$)
   *Meinel, C., Rhinow, H., & Köppen, E. (2013). Design Thinking Prototyping Cardset. Hasso Plattner Institut für Softwaresystemtechnik, Potsdam.
   *Sadler, J., Shluzas, L., Blikstein, P., & Katila, R. (2016). Building blocks of the maker movement: Modularity enhances creative confidence during prototyping. In Design Thinking Research (pp. 141-154). Springer, Cham.

(iii) Even when the time for implementing a creative idea remains the same, there is a huge difference between the implementation as a “one-shot-operation” versus a process of “building and testing, building and testing…”. Via repeated tests and prototype improvements, even implementations that start with little efficacy can be rendered high-performing over time. Therefore, the prospect of being able to test and improve designs throughout the implementation phase allows people to select new and daring ideas, because they can be rendered feasible prior to the final submission. By contrast, when the implementation is (expected to be) a one-time operation, this indeed offers little hope for improvements over time, so that well known and easy to implement concepts seem obviously preferable. In this particular study, no hint has been made to the possibility of tests and refinements in the implementation phase, thereby rendering a “play-safe strategy” a logically good choice for the production of somewhat effective prototypes.
Ref:
*Dow, S. & Klemmer, S. R. (2011). The efficacy of prototyping under time constraints. In Design thinking (pp. 111-128). Springer, Berlin, Heidelberg.

(iv) There can be a great difference between the instruction to implement an idea, versus the explicit instruction to implement varying ideas in parallel to probe a bandwidth of creative opportunities. In the latter case, there is also the possibility of instructing students to select at least one idea that seems well-feasible, and another idea that is highly original and would seem to yield very good effects if only it could be implemented (“dark horse” method). In this particular study, it seems to be the case that children were instructed to select (and implement?) two ideas. However, there was obviously no instruction to use this parallel prototyping opportunity in order to enhance more diverse thinking, including the implementation of at least one more extreme and daring idea.
Refs:
*Dow, S. P., Glassco, A., Kass, J., Schwarz, M., Schwartz, D. L. & Klemmer, S. R. (2010). Parallel prototyping leads to better design results, more divergence, and increased self-efficacy. Transactions on Computer-Human Interaction, 17 (4), 18:1-24.
*Dow, S., Fortuna, J., Schwartz, D., Altringer, B., Schwartz, D., & Klemmer, S. (2011, May). Prototyping dynamics: sharing multiple designs improves exploration, group rapport, and results. In Proceedings of the SIGCHI conference on human factors in computing systems (pp. 2807-2816).
*Dow, S. (2011). How prototyping practices affect design results. Interactions, 18(3), 54-59.
[https://www.designthinking-methods.com/en/4Prototypen/darkhorse.html](https://urldefense.com/v3/__https:/www.designthinking-methods.com/en/4Prototypen/darkhorse.html__;!!HJOPV4FYYWzcc1jazlU!8lko8B4c0dtEVikSSBTj3__lLSV9MobhAHWhwmLlOhD_kd-OIEBOBBxrsOFzUvHp5rVSw8ynvUqYLkgLxWiK0w$)

*Dow, S. & Klemmer, S. R. (2011). The efficacy of prototyping under time constraints. In Design thinking (pp. 111-128). Springer, Berlin, Heidelberg.

Response: We are really grateful for the reviewers’ detailed description of literature on prototyping, and we agree that we should integrate this in our manuscript. We have integrated this insightful knowledge into the manuscript (see page 20):

*‘Finally, it is possible that the creativity of the children’s final selected ideas was lowered, because children may be constrained by their own building capacity in their selection of original ideas (Sadler, et al., 2016). Children may wonder which idea they can actually build instead of selecting original ideas irrespective of their building skills. Further, several researchers have shown that multiple iterations and parallel prototyping contributes to an increase in both the quantity and creativity of prototypes produced, as it allows children to try out constructing multiple ideas in parallel into tangible products (e.g., Dow et al., 2011; Dow et al., 2010; Taranu, et al., in press). Therefore, instead of giving children only one opportunity to translate their idea into a tangible product, children may gain more experience by letting them repeatedly work on a prototype in parallel sessions to further refine their idea and this may boost the creativity of their idea.’*

c. It would be good to increase the conceptual clarity regarding the relationship of “originality” and “usefulness”, the two key dimensions characterizing creative products. It is not logically necessary that highly original ideas must be less useful / feasible than non-original ideas. In this sense, the Idea Assessment Probes have been constructed as ideas sets for testing purposes in creativity research. These sets include original and non-original ideas as well as useful/feasible and not useful/feasible ideas, with the two dimensions varying independently of each other. Even with these idea sets, empirical research has found that people concerned with idea implementation (in a managerial context) preferred non-original ideas. This was the case even though highly original ideas would have been as feasible. Therefore, the feasibility of implementation does not seem to be the only factor that drives people to shy away from original ideas in the idea selection phase.
   Ref:
   *von Thienen, J. P. A., Ney, S. & Meinel, C. (2019). Estimator socialization in design thinking: The dynamic process of learning how to judge creative work. In R. Beghetto and G. E. Corazza (eds.), Dynamic perspectives on creativity: New directions for theory, research and practice in education (pp. 67-99). Springer.

Response: We agree that feasibility of implementation is not the only factor that drives children away from original ideas in the idea selection phase, but the bias against originality may play a role as well. As described by the reviewer, the Idea Assessment Probes have indeed been constructed as ideas sets for testing purposes in creativity research. We attempted to have a balanced list of 20 ideas that varied in creativity, in a 2 (originality: low, high) by 2 (feasibility: low, high) as creative ideas have to be (a) original and (b) feasible. As can be seen below, this set includes original and non-original ideas as well as useful/feasible and not useful/feasible ideas.

|  | **Low feasibility** | |  | **High feasibility** | |
| --- | --- | --- | --- | --- | --- |
| **High originality** | - | *Make the toy elephant in such a way that it can drink water* |  |  |  |
|  | - | *Make the toy elephant in such a way that it can poop* |  |  |  |
|  | - | *Make a robot out of the toy elephant that you can control* |  | - | *Make magnets in the legs so that the toy elephant can be hung up or is able to climb* |
|  | - | *Make the toy elephant in such a way that it can fly* |  | - | *Make a zipper in the toy elephant so you can put stuff in it* |
|  | - | *Make the toy elephant in such a way that it can spit fire* |  |  |  |
|  | - | *Ensure that the toy elephant can move on its own* |  |  |  |
|  | - | *Make the toy elephant water resistant* |  |  |  |
|  | - | *Make balls in the feet of the toy elephant so it can roll* |  |  |  |
| **Low originality** |  |  |  | - | *Make the toy elephant in all kinds of different colors* |
|  |  |  |  | - | *Make the toy elephant bigger* |
|  | - | *Make sure the toy elephant can make sounds* |  | - | *Make clothes for the toy elephant, then you can dress and undress him* |
|  | - | *Make the toy elephant so that he can bounce* |  | - | *Make the toy elephant extra soft* |
|  |  |  |  | - | *Tie bandages around the toy elephant so the elephant is sick* |
|  |  |  |  | - | *Make a tablet on his belly with mini games* |
|  |  |  |  | - | *Leave out the tusks on the toy elephant* |
|  |  |  |  | - | *Make folding tusks with sharp points* |

However, the Idea Assessment Set also illustrates the difficulty of having to generate ideas that are both highly original and highly feasible. It includes original and non-original ideas as well as useful/feasible and not useful/feasible ideas, but it contains relatively more ideas that are highly feasible, but non-original and highly original, but not useful/feasible ideas. Hence, a negative correlation of -0.51 was obtained between idea originality and idea feasibility, which was statistically significant at a level of p ≤ 0.001 (in line with von Thienen, Ney, & Meinel, 2019). Inspired by the reviewers’ comments, we attempted to increase the conceptual clarity regarding the relationship of “originality” and “usefulness”, and we ourselves feel it is much clearer now (see page 16-17):

*‘We found that children inhibit themselves in selecting original ideas once there is an expectation of idea implementation. Hence, to move from creativity to innovation, this research shows that more focus may be put on the practicality of ideas. In a brainstorm between two people (i.e., dyad) about ways to improve a stuffed toy elephant, Glăveanu, Gillespie, & Karwowski (2019) have compared the practicality of ideas recorded on paper with ideas not recorded on paper, but only verbally expressed. In line with our findings, they found that dyads were more likely to write down practical ideas than original ideas. As such, it seems that the practicality of ideas becomes more important in the selection and implementation of ideas than in the generation of ideas. Furthermore, we found a trade-off between novelty and usefulness where children who expected implementation selected less original, but more feasible ideas than children who did not have an expectation of later implementing the selected ideas. According to the novelty-usefulness trade-off, highly original ideas are more likely to be judged as less feasible because they involve, by definition, a step into the unknown (Baer, 2012). Several scholars argue that these two criteria of creativity are often seen as incompatible and represent a fundamental tension or paradox (Frederiksen & Knudsen, 2017; Miron-Spektor & Beenen, 2015; Miron-Spektor, Gino, & Argote, 2011; Mueller, Melwani, & Goncalo, 2012; Nijstad, De Dreu, Rietzschel, & Baas, 2010; Zacher, Robinson, & Rosing, 2016). In line with von Thienen, Ney and Meinel (2019), we found a negative correlation of -0.51 between idea originality and idea feasibility. As such, the novelty-usefulness trade-off explains our finding that children select less original, but more feasible ideas once expecting implementation of those ideas. This implies that the expectation of idea implementation causes children to play it even safer with regard to practicality and to choose more feasible ideas rather than more original ones. In addition, our finding can also partly be explained by the bias against originality (Mueller et al., 2012). The most original ideas are often those that are radically different from existing solutions or practices, which often cause people to have ambivalent feelings towards novel ideas, because people often prefer the status quo or familiar ideas. Blair and Mumford (2007), for example, found that managers concerned with idea implementation prefer non-original ideas even when they are ideas that are both original as well as useful. We found that children without expected implementation selected more original ideas, they did not totally abandon feasible ideas (see Fig. 3).’*

**Both reviewer 1 and 2 made the following remark:**

d. It would be interesting to read the statistical correlation of idea "originality" and "usefulness" ratings by the children and the experts (reviewer #2). “(…) degrees of feasibility and originality of their selected ideas were negatively correlated” 🡪 Would it be possible to provide specific estimates? (reviewer #1).

Response: We agree with both reviewers that it is interesting and relevant for the reader to provide specific estimates.

|  | Feasibility | |
| --- | --- | --- |
| Originality | -0.357 ***  -0.512*** | Children  Experts |

As such, we added the correlation of idea “originality” and “feasibility” for experts in the manuscript (see page 17):

*‘In line with von Thienen, Ney and Meinel (2019), we found a negative correlation between originality and feasibility of 0.51 among experts, and 0.36 among children.*

e. It would be interesting to read if there might be a preference for unoriginal ideas that is even independent of feasibility considerations (e.g., a negative correlation of “originality” with “choice for implementation”, while partialising out the “feasibility” rating).

Response: We agree with the reviewer that it is an important question whether there is under the treatment conditions a preference for unoriginal ideas that is independent of feasibility considerations. This is our main reason to run the conditional logit model that simultaneously estimates the effects of children’s feasibility and originality ratings on their selection of ideas (see Model 3 from the conditional logit model). In this way, we partialize out the feasibility rating from the originality rating. Model 3 from Table 1 shows that the parameters of feasibility ratings (including interaction term with treatment) hardly change when controlling for originality. The interaction of the treatment with the originality rating reduces by about 1/3th. This means that to some extent students choose less original ideas just because more feasible ideas are on average less original, but that after partializing out the effects of feasibility students also choose less original ideas.

**Table 1.** Idea selection conditional on alternative ideas (odds ratios and Z-statistics)

|  | **Model 1** | |  | **Model 2** | |  | **Model 3** | |
| --- | --- | --- | --- | --- | --- | --- | --- | --- |
| Feasibility | 0.604 | *** |  |  |  |  | 0.621 | *** |
|  | (-11.05) |  |  |  |  |  | (-10.33) |  |
| Treatment*Feasibility | 2.073 | *** |  |  |  |  | 2.026 | *** |
|  | (12.29) |  |  |  |  |  | (11.78) |  |
| Originality |  |  |  | 1.296 | *** |  | 1.202 | *** |
|  |  |  |  | (5.94) |  |  | (4.13) |  |
| Treatment*Originality |  |  |  | 0.771 | *** |  | 0.859 | * |
|  |  |  |  | (-4.41) |  |  | (-2.52) |  |

*Notes*: Z-statistics are reported in parentheses to indicate statistical significance. Effects are interpreted as the probability of favoring idea *k* multiplied by a one-unit increase in that variable. Estimates greater than 1 are considered positive effects, while estimates smaller than 1 are considered negative effects. *** Statistical significance at the 0.10% level (Z-statistics > 3.10), ** Statistical significance at the 1% level (Z-statistics > 2.58), * Statistical significance at the 5% level (Z-statistics > 1.96).

Based on the reviewers’ comment, we have integrated the Table of the conditional logit model in the manuscript (see page 15). Further, we have rewritten and sharpened our rationale for the conditional logit model (see page 12):

*‘Since there is a negative correlation between feasibility and originality, a reduction of the originality of ideas in case of expected implementation might be the direct result of increased feasibility. To check whether children really reduce the originality of the idea, more than would just be expected based on this correlation, we ran a conditional logit model that simultaneously estimates the effects of children’s feasibility and originality ratings on their selection of ideas (39). In this way, we partialize out the feasibility rating from the originality rating. In brief, each child chooses two ideas of a set of 18 ideas to improve a stuffed toy elephant.’*

**1 Introduction**

**Reviewer 1 had the following remarks for the introduction:**

1) I understand what the Authors meant by “we conceptualize creativity as the level of creativity of the final selected idea (i.e., the originality and usefulness of an idea)” but it sounds somewhat tautological. I would say that in this study, creativity was understood as a set of specific characteristics of ideas that children selected, and these characteristics include originality and usefulness. To put it even more simply: creativity was conceptualized as the level of originality and usefulness of ideas.

Response: We agree with the reviewer that is sounds somewhat tautological, and have rephrased the sentence (see page 2):

*‘In this study, creativity was conceptualized as a set of specific characteristics of ideas that children selected, and these characteristics include originality and usefulness (10). Originality refers to the characteristic of an idea that is new and unusual, and usefulness refers to the characteristic of an idea that is potentially feasible or valuable (11).’*

2) Considering expected idea implementation to be an example of extrinsic constraints really caught my attention. I think this is an interesting view yet it may require some explanation. One could argue that creative process is an action that results in an original and valuable product. Thus, from this perspective, idea implementation seems to be almost definitional part of creativity (or creative process, to be more precise). Therefore, treating idea implementation as a kind of constraint is not very typical. I wonder whether the Authors could elaborate on that a bit more.

Response: We agree with the reviewer that the creative process is an action that potentially results in an original and valuable product. As such, idea implementation is indeed part of the creative process. However, many scholars focus either only on the generation of ideas, while others focus on the implementation of ideas. Furthermore, in practice, children are not always asked to immediately implement their newly generated ideas. They can also only be asked to think off novel and feasible ideas, without actual implementation. Hence, arguably, purely the expectation of children that they not only generate or select ideas, but also have to implement those ideas in practice can be seen as an extrinsic constraint. Furthermore, considering expected idea implementation as a form of extrinsic constraint stems from the literature on expected evaluation on creativity (14, 16). People intuitively evaluate their or others ideas after idea generation, and, therefore, idea evaluation is part of the creative process. Yet, this branch of literature argues that the fear of others’ evaluation impacts individuals’ creative performance: The expected evaluation of others may include pressure to attain a certain outcome or attempt to induce or coerce a person to act in specific way. This work similarly with expected implementation. Even though expected implementation stems from yourself, there will be others in the classroom seeing your attempt to implement ideas. Novel ideas are often risky and untested, and, therefore, a higher risk for children to fail in front of others. Based on the reviewers’ comment, we have rewritten this part (see page 3-4):

*‘Several findings in the social psychology literature suggest that extrinsic constraints, such as expected evaluation of ideas, hinder people’s creativity (e.g., 14-17). This may be so for several reasons. First, it has been argued that exerting external pressure and constraints may reduce the fun of performing a task, which in turn may reduce intrinsic motivation and, subsequently, creativity (15). This line of research has shown that people are more likely to produce creative work when they are intrinsically motivated, because they are free of extraneous concerns about contextual conditions and are able to concentrate their attention to the task itself (16). The expected implementation of ideas can also be seen as an extrinsic constraint, because when attempting to implement an original idea in practice, the teacher and other children witness your attempt, and this attempt may fail in front of them. As such, expected implementation may exert external pressure on children’s idea selection. Further, it has been found that people have a deep-seated desire to maintain a sense of certainty and to preserve the familiar (18). In idea selection, people often favor the more common ideas due to risk perception with creative ideas, as creative ideas are by definition uncertain, because they are often new and untested (19). According to the novelty-usefulness trade-off, creative ideas are often original, and the more original an idea is, the higher the risk perception whether an idea will work in practice (20), creating doubt as to whether the idea can be realized (12). As such, children may fear failure in building physical products out of original ideas. For these reasons, simply instructing children to select original ideas—without having to build their chosen idea into a tangible product—may already be a challenging task. We further theorize that this may become even more problematic if teachers additionally instruct children to build original ideas into tangible products, because this invites the question of whether their idea can actually be built in practice.’*

3) What kind of judgments do the Authors refer to in the following sentence: “When making judgments, people often favor the more common ideas due to risk perception with creative ideas, as creative ideas are by definition uncertain, because they are often new and untested.” Is it still a reference to Sorrentino’s and Roney’s work?

Response: We refer to the idea selection literature, and based on the reviewers’ comment, we have rewritten this sentence and added a reference (see page 4):

*‘In idea selection, people often favor the more common ideas due to risk perception with creative ideas, as creative ideas are by definition uncertain, because they are often new and untested (19).’*

4) “field settings allow a more realistic setting” 🡪 again, I know what was meant here, but I recommend rephrasing this part.

Response: We agree with the reviewer that this sentence needed rephrasing (see page 4):

*‘While laboratory experiments elicit the pressure of extrinsic constraints, they generally have a low ecological validity, because the experiment is done in an artificial environment. As such, field settings allow a more realistic setting where extrinsic constraints are elicited in the actual classroom.’*

**2 Methods**

**Reviewer 1 had the following remarks for the method:**

1) “The children (49.9% girls, 50.1% boys)” 🡪 I think reporting the percentage for either one or another would work.
2) Children’s age range could be moved from *Procedure* section to *Participants* section.

Response: We have left out the percentage of boys, and moved children’s age range from Procedure to the Participants section (see page 5):

*‘The children (49.9% girls) attended grade 6 (last grade of primary school) and were aged between 10 and 13 years (M = 11.6, SD = 0.48).’*

3) “To reduce the list of 63 ideas even further, a set of 20 ideas was selected to be presented to the children in the experiment (see Appendix C).” What were the criteria for this stage of selection?

Response: We agree with the reviewer that the criteria for the selection of 20 ideas out of a list of 63 ideas could be clarified. From the list of 63 ideas, we randomly selected 20 ideas for each participate to rate. In this list of 20 ideas, we attempted to have a balanced list of 20 ideas that varied in creativity, in a 2 (originality: low, high) by 2 (feasibility: low, high) matrix was used, as a creative ideas has to be (a) original and (b) feasible. As such, the list of 20 pre-defined ideas, include both original as well as non-original ideas, and feasible as well as unfeasible ideas. Based on the reviewers’ comments, we have rewritten this section (see page 8):

*‘To reduce the list of 63 ideas even further, a random set of 20 ideas was selected to be presented to the children in the experiment (see Appendix B). These 20 pre-defined ideas varied in creativity, in a 2 (originality: low, high) by 2 (feasibility: low, high), as a creative ideas has to be (a) original and (b) feasibility (10).’*

Furthermore, we have added this balanced list of 20 ideas in matrix in Appendix B:

|  | **Low feasibility** | |  | **High feasibility** | |
| --- | --- | --- | --- | --- | --- |
| **High originality** | - | *Make the toy elephant in such a way that it can drink water* |  |  |  |
|  | - | *Make the toy elephant in such a way that it can poop* |  |  |  |
|  | - | *Make a robot out of the toy elephant that you can control* |  | - | *Make magnets in the legs so that the toy elephant can be hung up or is able to climb* |
|  | - | *Make the toy elephant in such a way that it can fly* |  | - | *Make a zipper in the toy elephant so you can put stuff in it* |
|  | - | *Make the toy elephant in such a way that it can spit fire* |  |  |  |
|  | - | *Ensure that the toy elephant can move on its own* |  |  |  |
|  | - | *Make the toy elephant water resistant* |  |  |  |
|  | - | *Make balls in the feet of the toy elephant so it can roll* |  |  |  |
| **Low originality** |  |  |  | - | *Make the toy elephant in all kinds of different colors* |
|  |  |  |  | - | *Make the toy elephant bigger* |
|  | - | *Make sure the toy elephant can make sounds* |  | - | *Make clothes for the toy elephant, then you can dress and undress him* |
|  | - | *Make the toy elephant so that he can bounce* |  | - | *Make the toy elephant extra soft* |
|  |  |  |  | - | *Tie bandages around the toy elephant so the elephant is sick* |
|  |  |  |  | - | *Make a tablet on his belly with mini games* |
|  |  |  |  | - | *Leave out the tusks on the toy elephant* |
|  |  |  |  | - | *Make folding tusks with sharp points* |

4) I think the subsection “Idea selection performance” could actually consist of two parts, namely: “Idea evaluation” and “Idea selection”. Now it is blended and the reader might feel a bit confused about what was the selection all about. Actually, at present this subsection seems to describe the evaluation part of the study and not the selection. I understood the whole procedure correctly only when I looked at supplementary materials. I think making a distinction into evaluation and selection part of the study would make it clearer for the reader.

Response: We have rewritten this part of the article (measures), and have revised it by taking these comments of the reviewer into account. We also feel ourselves it is clearer now (see page 8-9):

*‘Idea evaluation task. From this list of 20 pre-defined ideas for the stuffed toy elephant, children were asked to evaluate each idea on their feasibility (with a 5-point rating scale). Similarly as in Charles and Runco (26), children were asked to select one of five faces: (1) frown = very difficult to craft this idea; (2) slight frown = difficult to craft this idea; (3) no expression = not difficult but also not easy to craft this idea; (4) slight smile = easy to craft this idea; (5) smile = very easy to craft this idea) that best showed what they thought of the idea. Next, the originality evaluation task was administered with a 10-point rating scale. The children were asked to estimate a hypothetical number of children (between 1 and 10) able to generate a given idea (26). Hence, the originality of an idea was operationalized as the degree to which children thought that a number of children, from 10 children in total, would have generated a similar idea. This measure is often used in creativity research to determine the originality of ideas (26-31), and the pilot study showed that children were better able to assess the originality of ideas in this way.*

*Idea selection task. After the evaluation task, children were asked to select the five most original ideas from the list of pre-defined ideas for the stuffed toy elephant. From these five ideas, they had to select the two most original ideas (original ideas were defined as ideas that children would rarely see). Based on children’s evaluation ratings, children’s idea selection performance is measured by children’s own-rated originality and feasibility level of each idea. Next to children’s own-rated feasibility and originality, we also have the average rating of the seven experts for feasibility and originality.’*

5) I am probably missing something but I am not sure how the outcome variable in OLS regression computed was. I understand it was the average rating for the two selected ideas but did the Authors averaged only a given child’s ratings for the two ideas selected? Or did they average the average scores from the whole sample that were given to the two selected ideas?

Response: The outcome variable in OLS regression was computed by taking the average of the feasibility and originality rating of the two ideas selected by the child (when regressing the feasibility and originality of each idea separately almost identical results are obtained). We have rephrased the sentence (see page 11):

*‘(…) we performed multivariate ordinary least squares regression with children’s average rating for the two selected ideas as outcome variables separately for feasibility and originality’.*

6) I appreciate how the Authors made use of conditional logit model to draw even more precise and detailed conclusions.

Response: We wish to thank the reviewer for this kind comment. We indeed felt that only an univariate ordinary least squares regression did not provide sufficient insight into children’s considerations in relation to feasibility and originality when taking a decision about the creativity of an idea. The conditional logit model allows us to simultaneously estimates the effects of children’s feasibility and originality ratings on their selection of ideas.

**Reviewer 2 had the following remarks for the method:**

7) The authors of the paper have compiled a great battery to assess control variables. However, in this regard the statistical analysis seems not very sensitive. It yields the highly surprising outcome that no control variable at all has a notable impact on the children’s choices of ideas, not even “risk preference” as measured with the Risk Taking 10-item scale from the Jackson Personality Inventory. The authors write: “Notably, the detrimental effect of instructing children to transform their ideas into tangible and physical products was consistent among children with different background characteristics (i.e., gender, age, ethnicity, socioeconomic status, prior level of achievement, school) and psychological characteristics (i.e., risk preference and personality traits). Hence, these findings are broadly applicable to children independent of demographic or psychological variables (S2).” Here, a more pinpointed statistical analysis would seem helpful.
   - For instance, what is the average originality of selected ideas in the 25% of children that are most risk-averse compared to the 25% who are most open to risk-taking? After all, later on the authors wish to conclude:

“This means that by focusing on end-products only, educators may run the risk of applauding the creativity of a small group of children who had the courage to try out original ideas, while at the same time failing to recognize the creative potential of a group of equally creative children who were perhaps too afraid to bring their ideas into practice.” This statement seems to imply that there IS a differential impact of instruction on students. Especially the more cautious students would choose feasible solutions, while the more daring students might still implement highly original ideas.

“Further, it is important for teachers to foster a psychologically safe classroom environment where children feel safe in playing and experimenting with ideas and materials, taking sensible risks, and making mistakes.“ Again, this passage suggests that there IS a differential impact of psychological attributes, such that students who feel safe (e.g., to fail) will be in a better position to develop their creativity, from initial ideation up to the implementation of final project outcomes.

Overall, especially in the context of education, it would be good to show the differential impact of psychological variables such as willingness to take risks on creative performance. After all, education could help to foster a mindset that helps students be creative, e.g. by being better able to cope with the uncertainty of trying out something that is so unique that it has not been tried by anyone before.

Response: We wish to thank the reviewer for the compliment that we have compiled a great battery to assess control variables. We have compiled this battery of control variables, because we believe in the importance of assessing whether the treatment effect is similar for children with different demographic and psychological characteristics. For this, we have conducted rigorous interaction-analyses with both demographic and psychological variables. We only found for conscientiousness a significant interaction effect. This means that more conscientious children tend to choose more feasible and less original ideas also when they are not in the treatment group, but their choice changes much less when they are assigned to the treatment. So both untreated and treated conscientious children show the behavior of others when they are treated. Girls and children from the lowest SES group choose more feasible ideas but do not have a significantly different treatment effect. Similar results are obtained when replacing the linear specification with a specification with two dummies for the bottom and top 25% of the students, as suggested by the reviewer. The results therefore show that there is not much heterogeneity in the treatment effects based on these observables, and we added this to the discussion (see page 17):

*‘These findings are broadly applicable to children independent of demographic or psychological characteristics. We only found an exception for the personality trait conscientiousness. The significant interaction effect illustrates that more conscientiousness children tend to choose more feasible ideas, also when they are not in the treatment group (S2). Yet, their choice changes much less when they are assigned to the treatment group. Hence, both untreated and treated conscientiousness children show the behavior of others when they are treated.’*

Furthermore, the reviewer is right that we create the suggestion in the discussion that there are heterogenous effects. We adjusted this in the discussion (see page 18-19):

*‘This means that by focusing on end-products only, educators may run the risk of applauding the creativity of a small group of children who succeeded into constructing original ideas into tangible products, while at the same time not recognizing the creative potential of a group of equally creative children who tried, but failed in constructing original ideas into physical products.’ (…) ‘For this, it is important for teachers to foster a psychologically safe classroom environment where* ***all*** *children feel safe in playing and experimenting with ideas and materials, taking sensible risks, and making mistakes. In such an environment, novel and unorthodox ideas are valued and failures are seen as a necessary and positive part of the learning process in support of creativity (61-63).’*

**3 Discussion**

**Reviewer 1 had the following remarks for the discussion:**

1) “We found that children who expected implementation selected less creative ideas—i.e., more feasible and less original (…)” 🡪 as creativity straddles both originality and usefulness (feasibility), it does not seem accurate to conclude that less creative ideas were selected. Instead, I think that the idea of originality-usefulness trade-off fits particularly well to this paper’s narration, and could be emphasized throughout the article.

Response: We agree with the reviewer, and we have rewritten part of our discussion. We first focus on our finding that children select less original ideas and link this to previous research. Then, we link our finding to the originality-usefulness trade-off (see page 16-17):

*‘We found that children inhibit themselves in selecting original ideas once there is an expectation of idea implementation. Hence, to move from creativity to innovation, this research shows that children may put more focus on the practicality of ideas. In a brainstorm between two people (i.e., dyad) about ways to improve a stuffed toy elephant, Glăveanu, Gillespie, & Karwowski (42) have compared the practicality of ideas recorded on paper with ideas not recorded on paper (but only verbally expressed). In line with our findings, they found that dyads were more likely to write down practical ideas than original ideas. As such, it seems that the practicality of ideas becomes more important in the selection and implementation of ideas than in the generation of ideas. (…) Furthermore, we found a trade-off between novelty and usefulness where children who expected implementation selected less original, but more feasible ideas than children who did not have an expectation of later implementing the selected ideas. According to the novelty-usefulness trade-off, highly original ideas are more likely to be judged as less feasible because they involve, by definition, a step into the unknown (e.g., 43-48). Several scholars argue that these two criteria of creativity are often seen as incompatible and represent a fundamental tension or paradox (e.g., 49-54). In line with von Thienen, Ney and Meinel (55), we found a negative correlation between originality and feasibility of 0.51 among experts, and 0.36 among children. As such, the novelty-usefulness trade-off explains our finding that children select less original, but more feasible ideas once expecting implementation of those ideas. This implies that the expectation of idea implementation causes children to play it even safer with regard to practicality and to choose more feasible ideas rather than more original ones.’*

2) “children inhibit themselves in selecting original ideas” 🡪 After all, isn’t it quite an adaptive recalibration? Again, as creativity requires both originality and usefulness, maybe it is not that bad to make children be more pragmatic and realistic when it comes to choosing an idea that they want to implement? This is just an observation, but I wonder whether the Authors could share some opinion on that.

Response: We agree that it is not bad to make children more pragmatic and realistic when it comes to choosing an idea that they want to implement. We acknowledge that creativity starts with the generation of creative ideas, and to move from creativity to innovation, more focus may be put on the practicality of ideas. However, the danger is that children unnecessarily abandon original ideas that are feasible as well, only because they are novel and deviate from status quo. This could lead to loss of potential ground-breaking ideas that we have not seen before or that may solve complex problem in new ways. Inspired by the reviewers’ comment, we have rewritten part of the discussion (see page 16):

*‘We found that children inhibit themselves in selecting original ideas once there is an expectation of idea implementation. Hence, to move from creativity to innovation, this research shows that children may put more focus on the practicality of ideas. In a brainstorm between two people (i.e., dyad) about ways to improve a stuffed toy elephant, Glăveanu, Gillespie, & Karwowski (42) have compared the practicality of ideas recorded on paper with ideas not recorded on paper (but only verbally expressed). In line with our findings, they found that dyads were more likely to write down practical ideas than original ideas. As such, it seems that the practicality of ideas becomes more important in the selection and implementation of ideas than in the generation of ideas.’*

3) “educators should focus not only on the end-product, but particularly on the decision-making process children go through in selecting creative ideas” 🡪 I absolutely agree!

Response: We are happy to hear that the reviewer agrees with one of our most important conclusions.

4) “We may also note that while this study investigated whether the expectation of idea implementation affects idea selection among primary school children, it did not investigate the underlying mechanisms why this happens (e.g., emotional aspects of idea selection).” 🡪 I appreciate this was acknowledged and mentioned. I would love to see this important line of work develop further in such a direction.

Response: We agree with the reviewer, and we are happy to announce that we are currently working on a follow-up study to shed more light on these underlying mechanism.

**Reviewer 2 had the following remarks for the discussion:**

5) In their paper, the authors need to be very careful in interpreting their results. This experiment has not tested the impact of students expecting to implement or even expecting to construct creative ideas in general terms. It has tested a very specific implementation procedure that aims at (i) refined prototypes, which (ii) require considerable building skills, (iii) constructed in a “one-time” implementation procedure, (iv) without using parallel designs as a means to foster divergent, creative thinking in the implementation phase. Educators should rather be recommended to delve deeper into the existing literature on how different implementation strategies impact creative thinking, rather than being warned about implementation steps as creating a bias against creativity in general terms.

Response: We agree with the reviewers’ comment, and we have rewritten the discussion (see page 20):

*‘Finally, it is possible that the creativity of the children’s final selected ideas was lowered, because children may be constrained by their own building capacity in their selection of original ideas (68). Children may wonder which idea they can actually build instead of selecting original ideas irrespective of their building skills. Further, several researchers have shown that multiple iterations and parallel prototyping contributes to an increase in both the quantity and creativity of prototypes produced, as it allows children to try out constructing multiple ideas in parallel into tangible products (e.g., 57, 60). Therefore, instead of giving children only one opportunity to translate their idea into a tangible product, children may gain more experience by letting them repeatedly work on a prototype in parallel sessions to further refine their idea and this may boost the creativity of their idea.’*

6) In their discussion of practical implications, the authors currently write: “In stimulating children’s creativity, it may be worthwhile for educators to […] guide children with highly original but seemingly unrealistic ideas to think of ways for the idea to be made feasible.” In this context, it can be important to note that this question does not require answers from scratch to be developed in a case-by-case approach, let alone by the students. There is strong empirical data on strategies how to render original ideas more effective in the implementation phase. This could be taught by educators in the first place. A highly effective approach to render original ideas more effective is iterative prototype testing, moving from rough prototypes (concept-based) to more refined prototypes over time.
7) Furthermore the authors posit: “In stimulating children’s creativity, it may be worthwhile for educators to support children in their intuitive judgments of original ideas […]”. In this regard, again there are strategies that educators can use to help students pursue original ideas. In particular, parallel prototyping with the explicit instruction to choose at least one daring, wild “dark horse” solution is an empirically substantiated method to ensure that highly original ideas do not get lost in the implementation phase.

Response: We agree with the reviewer that prototyping offers opportunities for children to render original ideas more effective. As such, we have rewritten the practical implications (see page 18-19):

*‘In stimulating children’s creativity, it may be worthwhile for educators to support children in their intuitive judgments of original ideas, in resisting peer pressures to conform, and guide children with highly original but seemingly unrealistic ideas to think of ways for the idea to be made feasible. There are several strategies that educators can use to render wild-sounding ideas into more useful or feasible ideas, and this may help children to pursue original ideas (e.g., 57-59). More specifically, wild-sounding ideas can be rendered more effective by means of parallel prototyping (57). For instance, educators could encourage children to imagine, try out constructing multiple ideas in parallel into tangible products. Another highly effective approach to render original ideas more effective is iterative prototype testing (57, 60). In iterative prototyping, educators could encourage children to test and refine their ideas multiple times, moving from non-refined prototypes – such as rough sketches – to refined prototypes – such as refined paper models or CAD drawing over time. Next to these strategies, educators could also explicitly instruct children to try out at least one daring, wild “dark horse” solution. This ‘Dark horse’ strategy calls for the exploration of wild ideas that may never otherwise be explored, and ensures that highly original ideas do not get lost in the implementation phase (59). For this, it is important for teachers to foster a psychologically safe classroom environment where all children feel safe in playing and experimenting with ideas and materials, taking sensible risks, and making mistakes. In such an environment, novel and unorthodox ideas are valued and failures are seen as a necessary and positive part of the learning process in support of creativity (61-63).’*

1) The paper discusses the impact of “conformist thinking” in children. For instance: “several studies show that children around this age begin a trend of increasingly conformist thinking that continues through high school (20-22). Accordingly, it can be expected that our findings may become even stronger among young adolescents.“ Here it is not clear why conformist thinking should lead to a bias against original ideas. After all, the role models with whom children/adolescents might want to conform could be teachers or peers who explicitly love wild and daring creative ideas. Maybe the logic of argumentation could be explained in more detail here. Alternatively, there might be a discussion regarding the importance of role models and the impact of educators as role models in this context.

Response: Inspired by the reviewers’ comment, we have expanded our line of reasoning. We feel ourselves it is much clearer now (*see page 19-20):*

*‘First, we investigated the effect of expected implementation on idea selection among children aged 10–13, because several studies show that children around this age begin a trend of increasingly conformist thinking that continues through high school (21-22). This manifests itself in the fact that children want to be as ‘normal’ as possible, and prefer to do everything the same as their peers. As a result, it is therefore often more important to them what children of the same age think of them and their behavior than their teachers or parents (64). Accordingly, it can be expected that our findings may become even stronger among young adolescents. However, it remains unclear how younger children or adults would perform in similar experimental conditions. Thus, while the present study provides a starting point in research on the effect of expected implementation on creativity, the question remains as to how expected construction of ideas into tangible and physical products affects creativity in younger and older age groups.’*
